# Assessing the mechanisms of cholesteryl ester transfer protein inhibitors

**DOI:** 10.1016/j.bbalip.2017.09.004

**Published:** 2017-09-12

**Authors:** Meng Zhang, Dongsheng Lei, Bo Peng, Mickey Yang, Lei Zhang, M. Art Charles, Kerry-Anne Rye, Ronald M. Krauss, Douglas G. Johns, Gang Ren

**Affiliations:** a Molecular Foundry, Lawrence Berkeley National Laboratory, Berkeley, CA 94720, USA; b Department of Applied Science & Technology, University of California, Berkeley, CA 94720, USA; c School of Medicine, University of California–San Francisco, San Francisco, CA 94110, USA; d School of Medical Sciences, Faculty of Medicine, University of New South Wales, Sydney, NSW 2052, Australia; e Children’s Hospital Oakland Research Institute, Oakland, CA 94609, USA; f Merck Research Laboratories, Rahway, NJ 07065, USA

**Keywords:** Anacetrapib, CETP, CETP bound to HDL, CETP inhibitor, Cholesteryl ester transfer protein, Dalcetrapib, Electron microscopy, HDL, LDL, VLDL, Torcetrapib

## Abstract

Cholesteryl ester transfer protein (CETP) inhibitors are a new class of therapeutics for dyslipidemia that simultaneously improve two major cardiovascular disease (CVD) risk factors: elevated low-density lipoprotein (LDL) cholesterol and decreased high-density lipoprotein (HDL) cholesterol. However, the detailed molecular mechanisms underlying their efficacy are poorly understood, as are any potential mechanistic differences among the drugs in this class. Herein, we used electron microscopy (EM) to investigate the effects of three of these agents (Torcetrapib, Dalcetrapib and Anacetrapib) on CETP structure, CETP-lipoprotein complex formation and CETP-mediated cholesteryl ester (CE) transfer. We found that although none of these inhibitors altered the structure of CETP or the conformation of CETP-lipoprotein binary complexes, all inhibitors, especially Torcetrapib and Anacetrapib, increased the binding ratios of the binary complexes (e.g., HDL-CETP and LDLCETP) and decreased the binding ratios of the HDL-CETP-LDL ternary complexes. The findings of more binary complexes and fewer ternary complexes reflect a new mechanism of inhibition: one distal end of CETP bound to the first lipoprotein would trigger a conformational change at the other distal end, thus resulting in a decreased binding ratio to the second lipoprotein and a degraded CE transfer rate among lipoproteins. Thus, we suggest a new inhibitor design that should decrease the formation of both binary and ternary complexes. Decreased concentrations of the binary complex may prevent the inhibitor was induced into cell by the tight binding of binary complexes during lipoprotein metabolism in the treatment of CVD.

## 1. Introduction

Elevated plasma low-density lipoprotein cholesterol (LDL-C) and decreased high-density lipoprotein cholesterol (HDL-C) levels are two major risk factors for cardiovascular disease (CVD) [1]. Drugs that decrease LDL-C levels (for example, statins) have consistently been shown to decrease the incidence of CVD. In contrast, drug-induced increases in HDL-C levels have not yet been clearly shown to decrease CVD events [2].

Cholesteryl ester transfer protein (CETP) plays a key role in the transfer of neutral lipids between HDL and LDL particles and contributes to the transfer of cholesteryl ester into atherogenic LDL particles. Previous genetic studies of a family with a well-established inherited CETP deficiency have revealed that mutations in CETP can lead to a splicing defect and are associated with elevated HDL-C levels [3,4]. To date, numerous CETP inhibitors have been identified and assessed in clinical trials. The CETP inhibitors that have been previously studied or are currently in phase III outcome studies include Torcetrapib [5], Dalcetrapib [6], Anacetrapib [7], Evacetrapib [8] and TA-8995 [9]. Despite the clinical interest in CETP inhibitors, their detailed mechanisms of action affecting CETP function and neutral lipid transfer remain poorly understood.

Human CETP is a plasma glycoprotein composed of 476 amino acids and has a molecular mass of ~53 kDa before post-translational modification (fully glycosylated CETP has a molecular weight of ~74 kDa) [10]. On the basis of its crystal structure, CETP has a banana-like shape with four structural components: an N-terminal β-barrel domain, a C-terminal β-barrel domain, a central β-sheet, and a C-terminal extension (a distorted amphipathic helix (i.e., helix X) involving Glu465-Ser476 at the C-terminus) [11].

Biochemical studies have revealed that CETP interacts with surface phospholipids of HDL particles via a hydrophilic/hydrophobic interaction [12,13]. Although a protein-lipid-involved binding system can be detected and analyzed [14], usually with co-sedimentation as-says [15] and micro-calorimetry [16], the particular domains of CETP involved in binding lipoproteins and the detailed binding mechanisms remain elusive. The difficulty in studying the mechanism of CETP lies in the heterogeneity of its lipoprotein substrates and in the softness and high flexibility of their complex three-dimensional (3D) structure [17–19]. These properties limit the application of the other experimental procedures. For example, gel shift studies are challenging for the separation of molecules with large variations in molecular mass (the molecular mass of LDL is ~10 times that of HDL and ~250 times that of CETP). Gel filtration is challenging for isolating soft molecules, especially the one containing lipids. Ultracentrifugal separation may cause detachment of CETP from the lipoprotein [20]. Fluorescence resonance energy transfer (FRET) studies are limited by difficulties in expressing the full-length apolipoprotein B 100 (an LDL-containing protein of ~500 kDa, among one of the largest proteins) and determining a method to produce the reconstituted LDL. Immunological quantification of lipoproteins may also be inaccurate because of different combinations of polypeptides and modifications of HDL [21,22]. Most importantly, the binding of CETP is not a stationary process. HDL can alter the shape and components along with time in an HDL-CETP mixture [23], thus causing difficulty in producing accurate quantitative measurements.

Electron microscopy (EM) has an advantage over traditional biochemistry assays in studying lipoproteins, because of the large variety of lipoprotein subclasses [24]. Our early EM studies have shown that CETP bridges HDL and LDL together, thereby forming a ternary complex [25] in which the N-terminal β-barrel domain inserts into the surface lipid monolayer of HDL. The observation of a ternary complex supports the “tunnel mechanism” of CETP for the transfer of neutral lipids between different lipoproteins. Our EM study has revealed that the binding between CETP and HDL is mediated by a protein-lipid interaction [26]. This protein-lipid interaction makes it possible for five or more CETPs to share one HDL substrate (more than the number of HDL-containing proteins). Recently, molecular dynamics (MD) simulations in a CETP study demonstrate that the N-terminal β-barrel domain is flexible [27,28] and can penetrate into the HDL surface, thereby facilitating the uptake of cholesteryl ester [29]. The latest all-atomic MD simulation shows that CEs can be transferred through the CETP tunnel under a series of driving forces [30]. A parallel study using coarse-grained MD simulation on a microsecond scale has also suggested that CETP possesses a high degree of conformational flexibility and can form a continuous tunnel traversing its long axis [28], through which CEs and triglycerides (TGs) can be directionally transferred in the absence of an additional driving force.

Although MD simulations have predicted several underlying CETP mechanisms in CE transfer [27,29–32], experimental mechanistic studies of CETP inhibition at the molecular level remain to be performed. Herein, we used EM techniques, including cryo-electron microscopy (cryo-EM) and optimized negative staining (OpNS), to investigate the effects of CETP inhibitors on the CETP-lipoprotein structure and their conformations under various incubation times.

## 2. Results

### Effects of CETP inhibitors (Torcetrapib, Anacetrapib and Dalcetrapib) on CETP structure

Cryo-EM is a commonly used method to study protein structures under near-native conditions because it prevents possible artifacts induced by fixatives and stains, such as lipid stacking and flatness. However, images of small proteins (< 100 kDa) generally are of very low contrast, thus making their visualization and 3D reconstruction a challenging process. Given that CETP is an approximately 53 kDa asymmetric molecule (~74 kDa for fully glycosylated CETP) that is too small for cryo-EM, optimized negative staining (OpNS) [33,34] was used to investigate how CETP inhibitors influence the CETP structure.

OpNS is a negative staining method that has been refined from conventional NS protocols [35] by using cryo-EM images of apolipo-protein E_4_ HDL as a control [33]. Notably, the OpNS protocol decreases the rouleaux artifact of lipoprotein particles [19,33]. OpNS has been validated through cryo-EM images of 84-base pair double-stranded DNA [36] and proteins with known structures, including GroEL and proteasomes [34]. The unique capability of OpNS to allow examination of small proteins has been documented with the 53 kDa cholesteryl ester transfer protein [25] and the IgG1 antibody and its peptide conjugates [37], which are challenging targets for cryo-EM imaging. For this reason, OpNS was chosen to examine the effects of CETP inhibitors on CETP structure.

**Fig. 1.**
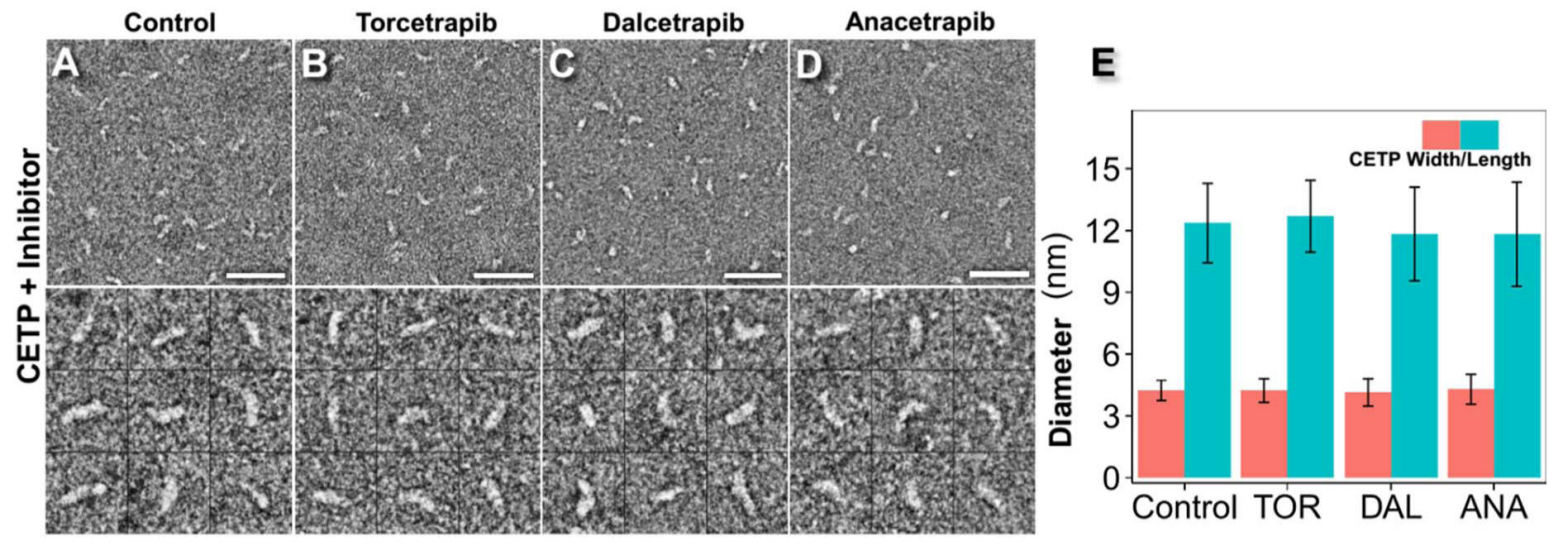
Effects of inhibitors on CETP structure by OpNS EM. A) Survey view of optimized negative-staining EM images (top panel) and representative particle images of CETP (bottom panel), B) CETP incubated with Torcetrapib, C) CETP incubated with Dalcetrapib, D) CETP incubated with Anacetrapib, each at 37 °C for up to 1 h. E) Statistical analysis of CETP dimensions before and after treatment with inhibitors. *p*-values of 0.13, 0.06 and 0.06 were obtained for length and 0.91, 0.24 and 0.51 for width after treatment with Torcetrapib, Dalcetrapib and Anacetrapib, respectively (Student’s *t*-test). Particle window size: A–D, 30 nm. Scale bars: 45 nm.

Recombinant human CETP harboring an N341Q [14] mutation at a glycosylation site to enhance production yield yet exhibiting identical behavior to wild-type CETP in lipid transfer assays [38] was incubated with each inhibitor at its maximal inhibitory concentration (approximately 10 μM) [14]. After 1 h of incubation, samples were prepared using OpNS and examined by EM. As a control, CETP was incubated with inhibitor buffer only. On the basis of a survey micrograph and representative particle images, CETP appeared to have a banana-like shape, similar to its crystal structure [11]. No obvious polymerization, aggregation or conformational changes were observed under any of the experimental conditions (Fig. 1A–D, Supplemental Fig. 1–4). In the control sample (Fig. 1A), CETP measured 12.4 ± 1.9 nm in length and 4.2 ± 0.5 nm in width. The dimension was similar to that in crystals (12.3 nm in length and 4.3 nm in width) [11] and in solution, as suggested by MD simulations [27]. After incubation with Torcetrapib (Fig. 1B), CETP measured 12.7 ± 1.7 nm in length and 4.2 ± 0.6 nm in width, dimensions similar to those of free CETP as well as to the crystal structure of CETP bound with Torcetrapib (12.3 nm in length and 4.3 nm in width) [39]. The similar dimensions of free CETP and Torcetrapib-bound CETP measured with our method are consistent with that measured from the crystal structure, thus suggesting that our method is reliable for examining the structure and conformational changes of CETP.

Incubations with Dalcetrapib (Fig. 1C) and Anacetrapib (Fig. 1D) resulted in CETP lengths of 11.8 ± 2.3 nm and 11.8 ± 2.5 nm, respectively, and widths of 4.1 ± 0.7 nm and 4.3 ± 0.7 nm, respectively. Although Dalcetrapib and Anacetrapib appeared to have slightly decreased the length of CETP by approximately 0.6–0.9 nm (approximately 6.8%) compared with the free CETP and Torcetrapib-bound CETP, the differences in length were significantly smaller than the measured standard derivation (2.3–2.5 nm, owing to the various CETP orientations when they land on the EM grid). Therefore, these differences were determined to be statistically insignificant (Fig. 1E), thus suggesting that the inhibitors do not induce significant conformational changes in CETP.

### Cryo-EM images and 3D reconstruction of the CETP-HDL_3_ complex

**Fig. 2.**
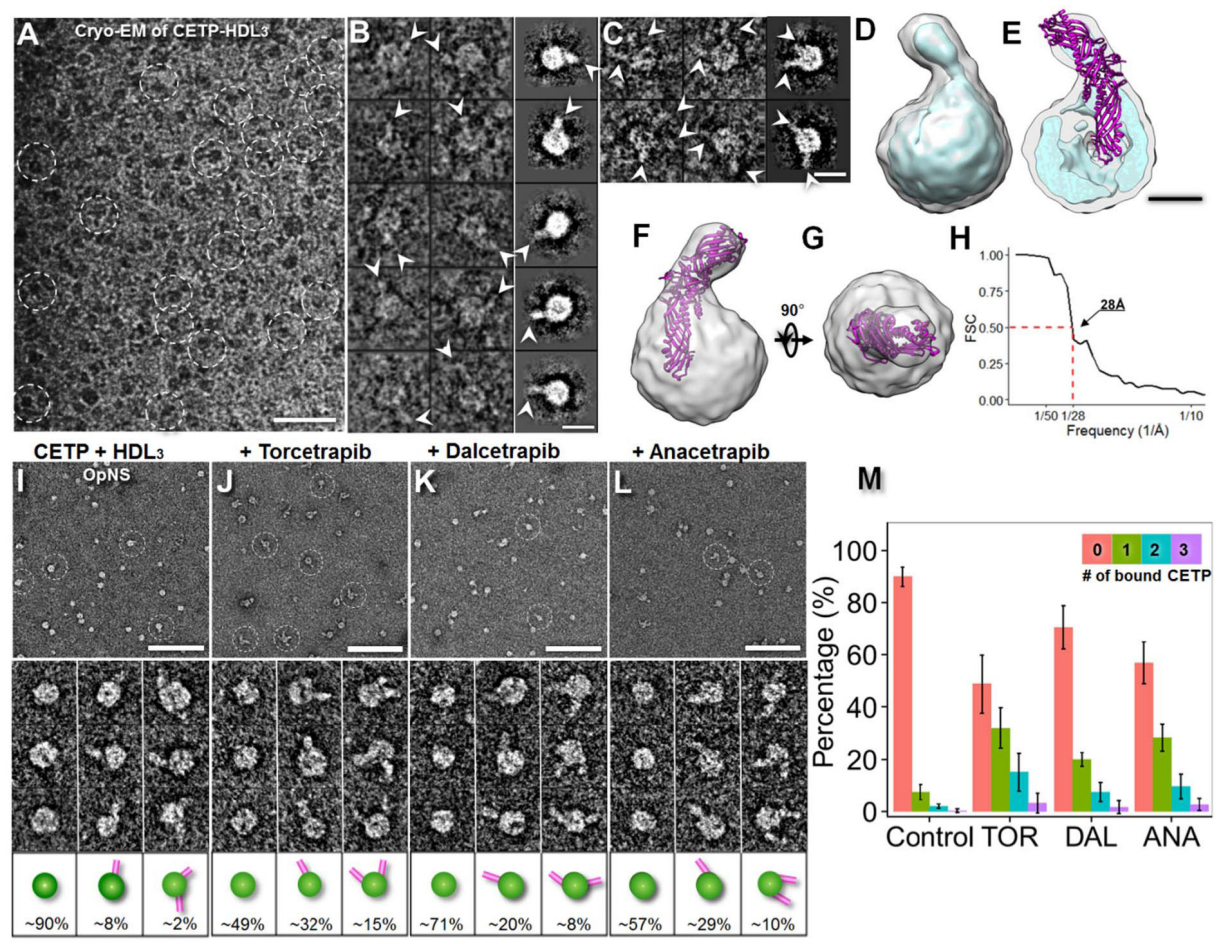
Effects of inhibitors on CETP bound to HDL, as determined by cryo-EM and OpNS EM. A) Cryo-EM survey view of the complexes of CETP bound with human plasma HDL_3_ embedded in vitreous ice (dashed circles). B) Representative cryo-EM images (contrast inverted, left column) and reference-free class averages (shown in the right column) of the complexes of one HDL_3_ bound to one CETP molecule and C) one HDL_3_ bound to two CETP molecules. D) Cryo-EM 3D density map of the CETP-HDL complex reconstructed by a single-particle 3D reconstruction method from a relatively homogenous population of particles (3200 complexes, approximately 13% of total particles) displayed in two contour levels (the gray contour level corresponds to the molecular volume of the complex, whereas the cyan contour level corresponds to approximately 37% of the molecular volume). E) Cutaway surface view showing that the spherical HDL has a diameter of approximately 97 Å with an approximately 20 Å thick high-density shell and an approximately 50 Å diameter inner low-density core. F) and G) Two perpendicular views of the CETP-HDL cryo-EM reconstruction showing the crystal structure of the docked CETP within the envelope of the EM density map. An approximately 55 Å-long portion of the CETP N-terminal penetrated or merged with the HDL surface. H) The FSC curve showing that the resolution of the cryo-EM single-particle 3D reconstruction is approximately 28 Å according to the 0.5 Fourier shell correlation criterion. I) OpNS EM survey images (top panel), representative particle images (middle panel) and the corresponding particle cartoons with their populations (bottom panel) of the samples of HDL_3_ incubated with CETP. The CETP-HDL complexes are indicated by white dashed circles. The sample was also repeated under co-incubation with J) Torcetrapib, K) Dalcetrapib or L) Anacetrapib. The percentages of HDL particles involved in binding with no CETP, binding with one CETP and binding with two CETPs are shown at the bottom of the corresponding cartoons. The percentage of HDL particles binding more than two CETPs is not shown. The percentage of HDL was calculated by dividing the total number of HDL + CETP binary complexes by the total number of HDL particles (including the particles forming into binary complexes). M) Histogram of the percentage of CETP-bound HDL over the entire HDL population. *p*-values of 2.20 × 10^−16^, 1.79 × 10^−7^ and 1.95 × 10^−14^ were obtained for Torcetrapib, Dalcetrapib and Anacetrapib, respectively, via Pearson’s chi-square test. Particle window size: I–L, 30 nm. Scale bars: A, 50 nm; B and C, 10 nm; E, 4 nm; I–L, 100 nm.

Given that the molecular weight of CETP-HDL_3_ is approximately 300 kDa, we used cryo-EM to examine its structure at −178 °C. Cryo-EM micrographs of CETP incubated with human plasma HDL_3_ at molar ratios of 3:1 embedded in vitreous ice contained spherical-, rodand garlic bulb-shaped particles (Fig. 2A). Selected images of the garlic bulb-shaped particles indicated that the complexes were composed of 8.9–11.5 nm spherical HDL_3_ with an approximately 8.1 nm rod-shaped protrusion, as viewed from an orientation showing the longest protrusion (the first two columns in Fig. 2B). Statistical analysis showed that 11.7% of HDL_3_ was bound to CETP, of which < 5% of the particles possessed more than one protrusion (first two columns in Fig. 2C). The conformation was clearly discernible from the selected reference-free class-averages (right column in Fig. 2B and C). Because HDL_3_ is heterogeneous in terms of its diameters and components and varies in its binding to CETP, a small subpopulation (approximately 13%; i.e., approximately 3200) with a relatively homogenous structure of CETPHDL_3_ complexes was selected from an original pool of over 24,000 complexes. The convergence of the structure from this subpopulation of particle images provided a statistically defined and robust density map displaying the most prominent and reliable structural features of CETPHDL_3_ (Fig. 2D).

A 3D density map of CETP-HDL_3_ at approximately 28 Å resolution (Fig. 2D and H) was reconstructed through a single-particle reconstruction protocol [18]. The map reveals a spherical HDL with a dimension of approximately 93 Å × 97 Å × 101 Å attached to a CETP protrusion with a dimension of approximately 25 Å × 25 Å × 70 Å (Fig. 2E). The CETP conformation and dimensions were similar to those obtained from the OpNS images of CETP bound to a 9.6 nm recombinant HDL (i.e., approximately 25 Å × 25 Å × 80 Å) [25]. By rigid-body docking of the crystal structure of CETP into the envelope of the cryo-EM density map at a contour level of 4.918, we found that CETP has a 55 Å length that penetrates or completely merges with the HDL surface (Fig. 2F and G). This length is approximately 10 Å deeper than that measured from the OpNS 3D reconstruction of CETP bound to recombinant HDL [25]. This difference may be due to variation in the organization and composition of fatty acids on the curved spherical HDL_3_ surface, as opposed to the planar organization of the phospholipids in discoidal reconstituted HDL, affecting interactions with CETP.

### OpNS-EM images of the CETP-HDL_3_ complex

By examining the same sample of CETP-HDL_3_ through OpNS EM (Fig. 2I), we observed features essentially identical to those identified using cryo-EM (Fig. 2A–C), including the penetration of CETP into the HDL surface. Although the OpNS indicated that the diameter of HDL_3_ is approximately 10% larger than that measured from cryo-EM images, the percentage of HDL_3_ bound to CETP differed from that measured from cryo-EM images by < 2% (9.9% vs. 11.7%). This difference in diameter measurement may have been caused by the sample flatness with OpNS, the dynamics of CETP binding on the HDL_3_ surface, or the low contrast cryo-EM images of CETP on the surface of heterogeneous HDL, which make classification and averaging a challenge.

Although the cryo-EM technique has the advantage of imaging samples under a near native buffer, this technique is challenging for directly capturing small molecules (< 150 kDa, such as 53 kDa CETP). Moreover, particles in the cryo-EM image may be influenced by the following processes: i) the protein with a larger hydrophobic surface is easier to remove by filter paper during blotting processing; ii) the supporting holey film has electrostatic properties that are different from those of the empty hole after glow-discharge, thus causing proteins with opposite charge are easier to be adsorbed by the film while particles have less charges present more into the hole area; and iii) the distribution of particle sizes may be regulated by the ice thickness within a hole, such that large particles are often pushed to the edge of the holes [17,26,40]. In the OpNS method, the excess sample solution was removed by touching the filter paper to the entire grid backside (opposite the carbon side) [33,34,41] without any direct interaction with the sample solution on the grid. Thus, the process had less influence on lipoproteins with heterogeneous size and different surface hydro-philicities. Moreover, given that the OpNS images i) exhibit the same 3D structural conformation of HDL_3_ bound to CETP as those obtained with cryo-EM; ii) reveal the same percentage of HDL_3_ bound to CETP as those from cryo-EM images; iii) have a much higher image contrast for the CETP portion than those obtained with cryo-EM; and iv) allow the examination of a huge number of samples, such as ~900 samples in this study, within an affordable time period, we used OpNS as the primary method to investigate the effects of inhibitors on the CETP-lipoprotein binary and ternary complexes in the following experiments.

### Effects of inhibitors on the conformation of the CETP-lipoprotein binary complex

To investigate the inhibitory effects of Torcetrapib, Dalcetrapib and Anacetrapib on binary interactions between CETP and the lipoproteins, we incubated each inhibitor (approximately 10 μM, for 1 h) with CETP first and then combined with one of the plasma lipoprotein subclass (i.e., HDL_3_, LDL or VLDL at molar ratios of 3:1, 9:1 and 9:1, respectively) at 37 °C for up to 1 min. As a control, the CETP/lipoprotein samples were incubated alone with buffer. All samples were prepared using OpNS and examined by EM.

Survey micrographs and representative particle images of samples in which HDL_3_ was incubated with CETP (Fig. 2I–L, Supplemental Fig. 5–8) showed that in all samples, rod-shaped CETP molecules had penetrated the spherical surfaces of the HDL_3_ molecules. These results are consistent with the cryo-EM results and results from previous studies [25], thus suggesting that the inhibitors did not cause appreciable conformational differences among the samples. The micrographs also showed that HDL was able to bind up to five CETP molecules. However, CETP was not observed to act as a bridge between HDLs. Statistical analyses indicated that the HDL particles had very similar diameters and shapes under all conditions, with diameters of 12.28 ± 1.91 nm (control), 12.05 ± 1.49 nm (Torcetrapib), 12.30 ± 1.79 nm (Dalcetrapib), and 12.27 ± 1.75 nm (Anacetrapib). The inhibitors also increased the percentages of CETP-bound HDL by 3- to 5-fold; 9.9% bound HDL was found in the control, which increased to 50.5% (Torcetrapib), 29.2% (Dalcetrapib) and 43.1% (Anacetrapib) (Fig. 2M) with significant *p*-values (Torcetrapib: 2.20 × 10^−16^, Dalcetrapib: 1.79 × 10^−7^ and Anacetrapib: 1.95 × 10^−14^) determined by Pearson’s chi-square test. These results suggest that the inhibitors significantly increased the binding affinity of CETP to HDL, a result consistent with the hypothesis that increased binding affinity might improve CETP inhibition based on biochemistry experiments [14].

Anacetrapib and Torcetrapib significantly increase the CETP/HDL binding ratio relative to Dalcetrapib [42,43]. The crystal structure of the CETP-Torcetrapib complex has been reported and it is likely that Anacetrapib and Torcetrapib have similar binding positions in the CETP pocket [32,42]. The binding position of Dalcetrapib, is unknown, but the fact that it has a distinct chemical structure and a much lower CETP binding affinity comparing with Anacetrapib and Torcetrapib, suggests that it may bind to a different site within the CETP molecule. MD simulations have shown that Torcetrapib and Anacetrapib increase the dynamics of both of CETP N- and C-terminal β barrial domains [44], which might facilitate the CETP insertion process, and thus explain the increased CETP binding affinity of both of these inhibitors.

The EM experiments described above suggest that the portion of CETP that merged into the HDL surface is approximately 45–55 Å in length, a result consistent with previous observations [25]. Two hypotheses may relate the mechanisms of the merging process of CETP with HDL surface lipids: i) a fusion mechanism in which the CETP β-barrel domain is completely unfolded and fused with the HDL surface lipid monolayer; and ii) a penetration mechanism in which the CETP β-barrel domain is completely inserted into the HDL surface lipid monolayer and extended to its central neutral lipid core, as previously proposed [25]. To evaluate the possibility of the fusion mechanism, we conducted the following calculation. By assuming that the 5-nm portion of CETP merged with the HDL surface via a large-scale conformational change, the original surface of this portion of CETP would occupy at least 30% of the HDL surface, on the basis of the equation, d×L/ (4×D) (where D is the HDL diameter, and L and d are the penetrated depth and diameter of CETP, respectively). Given that approximately half of the HDL surface is occupied by amphipathic apolipoproteins, no more than two CETPs should be observed on HDL surfaces. However, the above results, our previous experiments [25,26] and the recent report by Lauer et al. [45] all show that four or more CETPs can simultaneously bind to one HDL, thus suggesting that the fusion mechanism is less likely.

To evaluate the possibility of the penetration mechanism, we conducted another calculation. On the basis of the assumption that a CETP β-barrel domain completely inserted into the HDL surface without involving any conformational change, the β-barrel domain (5 nm in length and 2.5 nm in diameter) would increase the HDL diameter ~1.5% times; i.e., from ~10 nm HDL to ~10.15 nm, on the basis of the equation ${\text{D}}^{\prime }=\sqrt[{3}]{{\text{D}}^{3}+\frac{3}{2}{\text{Ld}}^{2}}$ (where D and *D*^*′*^ are the HDL diameter before and after CETP penetration, respectively, and L and d are the penetrated depth and diameter of CETP, respectively). Given that ~80% of binding HDLs bind only one CETP, the small change (< 2%) in HDL diameter is similar to the measured standard derivation of the HDL diameter, thus supporting the penetration hypothesis.

**Fig. 3.**
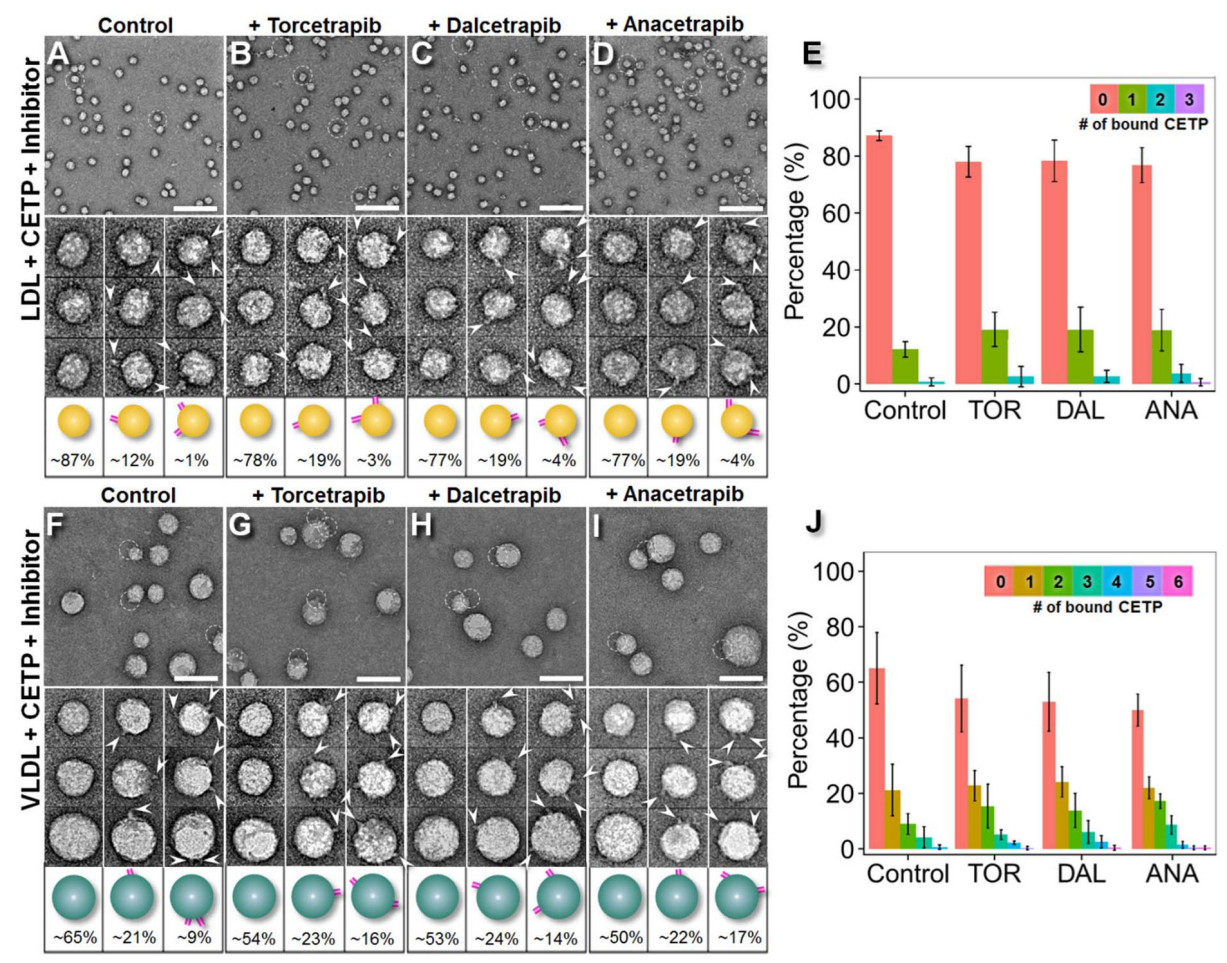
Effects of inhibitors on CETP bound to LDL/VLDL by OpNS EM. A) OpNS EM survey images (top panel), representative particle images (middle panel) and the corresponding particle cartoons with their populations (bottom panel) of human plasma LDL incubated with CETP and the sample after incubation with B) Torcetrapib, C) Dalcetrapib, or D) Anacetrapib at 37 °C. E) Histogram of the percentage of CETP-bound LDL over the entire LDL population (with corresponding *p*-values of 0.05, 0.04, and 0.02 for Torcetrapib, Dalcetrapib and Anacetrapib, respectively). The percentage of LDL was calculated by dividing the total number of LDL + CETP binary complexes by the total number of LDL particles (including the particles incorporated into binary complexes. F) OpNS survey images (top panel), representative particle images (middle panel) and the corresponding particle cartoons with their populations (bottom panel) of human plasma VLDL incubated with CETP and the sample after incubation with G) Torcetrapib, H) Dalcetrapib, or I) Anacetrapib at 37 °C. J) Histogram of the percentage of CETP-bound VLDL over the entire VLDL population (with corresponding *p*-values of 7.00 × 10^−3^, 6.69 × 10^−3^ and 4.01 × 10^−4^ for Torcetrapib, Dalcetrapib and Anacetrapib, respectively). Statistics were calculated with Pearson’s chi-square test. The percentages of LDL and VLDL particles involved in binding no CETP, one CETP and two or more CETPs are shown at the bottoms of the corresponding cartoons. The percentages of LDL and VLDL particles binding more than two CETPs are not shown. Particle window size: A–D, 45 nm; F–I, 60 nm. Scale bars: A–D, 140 nm; F–I, 70 nm.

Survey micrographs and representative particle images of samples in which LDL was incubated with CETP (Fig. 3A–D, Supplemental Fig. 9–12) showed LDL surfaces penetrated by rod-shaped CETP molecules in all samples. The conformations of the binary LDL-CETP complexes (Fig. 3A–D, bottom panel) were consistent with results from previous studies [25] and did not differ considerably among the samples. Approximately 17% of the observed LDLs were bound to one CETP molecule each, whereas approximately 3% were bound to additional CETPs. The LDL particles displayed very similar diameters and shapes even in the presence of the inhibitors, measuring 23.6 ± 1.3 nm (control), 24.8 ± 1.3 nm (Torcetrapib), 24.4 ± 1.4 nm (Dalcetrapib) and 23.5 ± 1.3 nm (Anacetrapib). However, the inhibitors nearly doubled the percentages of CETP-bound LDL, which were 12.9% (control), 21.8% (Torcetrapib), 22.6% (Dalcetrapib) and 23.4% (Anacetrapib) (Fig. 3E). The corresponding *p*-values were 0.05 (Torcetrapib), 0.04 (Dalcetrapib) and 0.02 (Anacetrapib) according to Pearson’s chi-square test. These results suggested that the inhibitors increased CETP binding affinity to LDL, although not to the same degree as HDL.

**Fig. 4.**
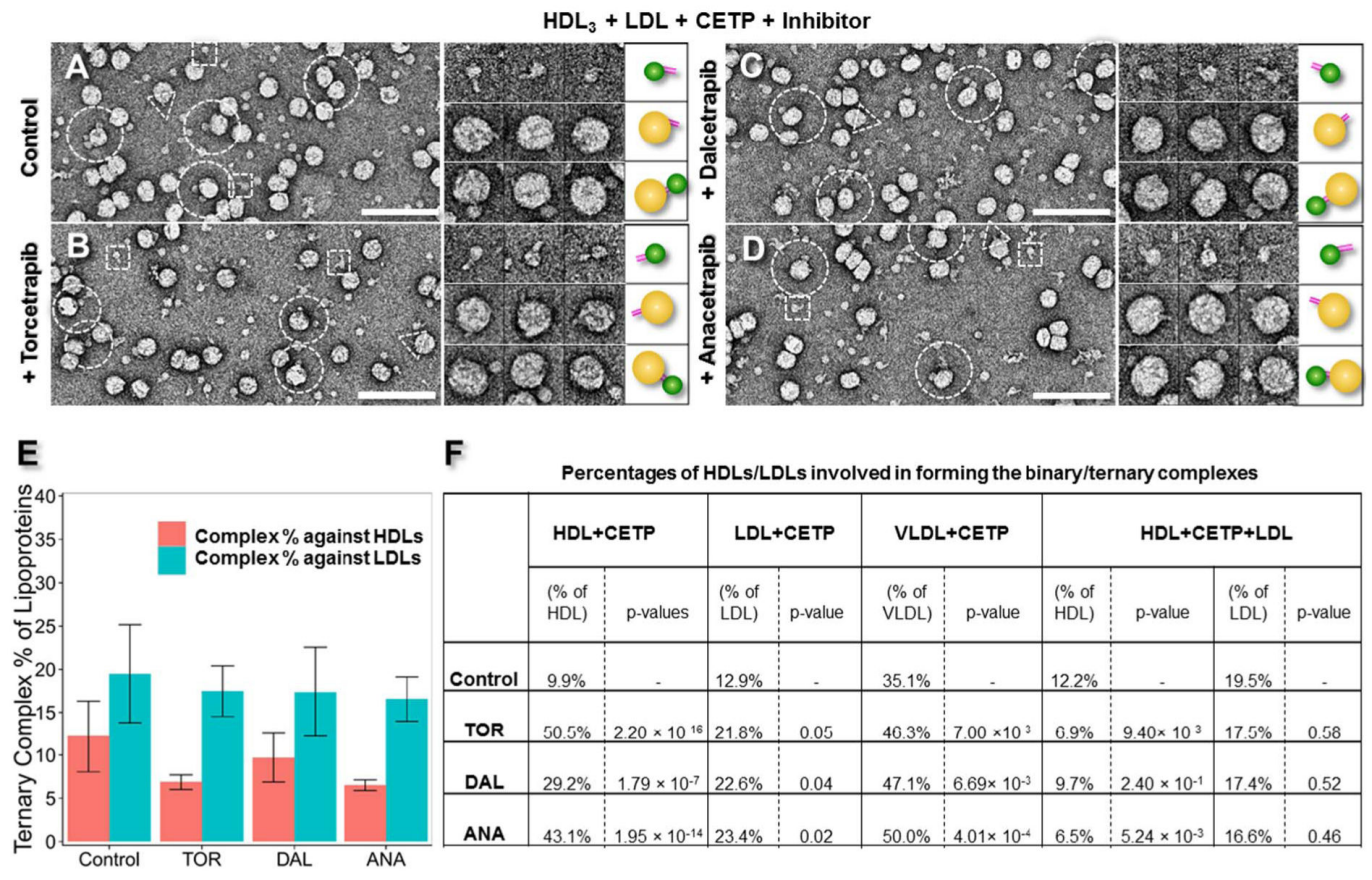
Effects of inhibitors on CETP in bridging HDL and LDL by OpNS EM. A) OpNS survey EM images (left panel), representative particle images (middle panel) and corresponding particle cartoons (right panel) of the CETP incubated with human plasma HDL_3_ and LDL simultaneously at 37 °C. The sample was also examined in the presence of B) Torcetrapib, C) Dalcetrapib, or D) Anacetrapib. In the survey views, the CETP-HDL_3_, CETP-LDL and HDL-CETP-LDL complexes are indicated by white dashed squares, triangles and circles, respectively. Images of the CETP-HDL_3_ complexes are shown in the top panels, the CETP-LDL complexes are shown in the middle panels and the LDL-CETP-HDL_3_ complexes are shown in the bottom panels. E) Statistical analyses of the percentages of LDL or HDL in an HDL-CETP-LDL ternary complex with corresponding *p*-values for LDL (Torcetrapib: 0.58; Dalcetrapib: 0.52 and Anacetrapib: 0.46) and for HDL (Torcetrapib: 9.40 × 10^−3^; Dalcetrapib: 2.40 × 10^−1^ and Anacetrapib: 5.24 × 10^−3^). The statistical analysis was conducted with Pearson’s chi-square test. F) A collection of all statistics of the binary and ternary complex ratios formed after CETP inhibitor treatment. The percentage of bound HDL was calculated by dividing the total number of HDL + CETP + LDL ternary complexes by the total number of HDL particles (including the particles in binary and ternary complexes). The percentage of bound LDL was calculated by dividing the total number of HDL + CETP + LDL ternary complexes by the total number of LDL particles (including the particles in binary and ternary complexes). Particle window size: A–D, 48 nm. All scale bars: 80 nm.

Survey OpNS micrographs and representative particle images of the samples of VLDL incubated with CETP (Fig. 3F–I, Supplemental Fig. 13–16) show VLDL surfaces penetrated with rod-shaped CETP molecules for all binding conditions. The observed conformations of the binary VLDL-CETP complexes (bottom panels in Fig. 3F–I) were consistent with results from previous studies [25] and exhibited no appreciable differences among the samples. Approximately 20% of the observed VLDLs interacted with one CETP molecule, and approximately 30% were bound to two or more CETPs (Fig. 3G–I). In contract, most LDL bound to one CETP, however, very small amount of LDL was also observed bound to two and more CETPs. This variation of CETP molecule numbers per LDL particle may due to the lipoproteins being isolated in the 1.006–1.069 g/mL density range, which includes intermediate density lipoproteins (IDLs), VLDL remnants. The VLDL particles had very similar mean diameters and shapes under all conditions, measuring 38.28 ± 7.12 nm (control), 37.9 ± 7.0 nm (Torcetrapib), 38.7 ± 5.7 nm (Dalcetrapib) and 38.6 ± 6.3 nm (Anacetrapib). However, the inhibitors increased the percentage of VLDL particles that bound CETP from 1/3 to nearly 1/2; the new values were 35.1% (control), 46.3%, (Torcetrapib), 47.0% (Dalcetrapib) and 50.0% (Anacetrapib) (Fig. 3J), with significant *p*-values (Torcetrapib: 7.00 × 10^−3^; Dalcetrapib: 6.69 × 10^−3^ and Anacetrapib: 4.01 × 10^−4^) determined with Pearson’s chi-square test. In comparing to that the inhibitors increased the CETP binding to HDL by ~20–40% and to LDL by ~9–11% (Fig. 4F), the inhibitors increased CETP binding to VLDL for ~11–15% (from 35.1% to 46.3–50.0%), which is lesser extent than to HDL but greater than to LDL. An hypothesis of that the inhibitors increased more the binding affinity of CETP to VLDL than to LDL is that VLDL naturally as a source in the hetero-exchange between CE and TG [46] serves an additional function in TG transfer, which may cause more CETPs to bind to VLDL than LDL.

Notably, in the above statistical analyses, the CETP binding number was calculated by counting the observed protrusions extending from the edge of lipoprotein spheres. A true representation of all the CETP-lipoprotein interactions should include those CETPs that interact on the top and bottom of the lipoprotein sphere, especially for large lipoprotein particles. In a previous study of CETP binding to liposomes [26], we have calculated the probability (℘) (i.e., the ratio of the CETP visible area on a sphere vs. the sphere overall area) as a function of the diameter of the sphere (*d*) and the length of CETP protrusion (*l*): $℘=\cos\left[sin^{-1}\left(\frac{d}{d+2l}\right)\right]$ under the assumption that there is no preferred orientation for CETP in binding a substrate of carbon film. Under this calculation (with a CETP protrusion length of 8 nm), a true representation of the CETP binding number to a 10–30 nm diameter lipoprotein particle should be increased by 9–32% compared with the observed value. However, because the amount of CETP binding to lipoprotein is naturally low (with few lipoproteins attached to more than three CETPs) (Fig. 2M, 3E and J), adjustment with this probability did not result in a noticeable difference.

### Effects of CETP inhibitors on the ternary complex conformation of CETP bound to HDL and LDL

To investigate the effects of the inhibitors on the HDL-CETP-LDL ternary complex conformation, we pre-treated CETP with each inhibitor (approximately 10 μM, for 1 h) before co-incubating with HDL_3_ and LDL at a molar ratio of 9:3:1 at 37 °C for up to 1 min. As a control, a sample of inhibitor buffer co-incubated with CETP, HDL_3_ and LDL was also prepared. All samples were prepared using OpNS and examined by EM.

Survey micrographs and representative particle images (left panels in Fig. 4A–D and Supplemental Fig. 17–20) show rod-shaped CETPs bridging spherical HDL_3_ particles (small diameter) to spherical LDL particles (large diameter), thus leading to the formation of ternary complexes in all of the above samples. The conformations of the ternary complexes (left two panels in Fig. 4A–D, left panels in Supplemental Fig. 17–20) were consistent with results from previous studies [25], and no noticeable conformational differences were observed between the samples, regardless of whether the inhibitors were present. The statistical analysis of the percentage of the ternary complexes relative to the total LDLs (including the free LDLs and the LDLs in binary and ternary complexes) (Fig. 4E) showed little difference among the different inhibitors; i.e., 19.5% for the control, 17.5% for Torcetrapib (*p*-value of 0.58), 17.4% for Dalcetrapib (*p*-value of 0.52) and 16.6% for Anacetrapib (*p*-value of 0.46) (Fig. 4F). However, the percentage of the ternary complexes relative to total HDL (including free HDL and HDL in binary and ternary complexes) did show a difference for each inhibitor; i.e., 12.2% for control, 6.9% for Torcetrapib (*p*-value of 9.40 × 10^−3^), 9.7% for Dalcetrapib (*p*-value of 2.40 × 10^−1^) and 6.51% for Anacetrapib (*p*-value of 5.24 × 10^−3^) (Fig. 4F). All *p*-values were calculated relative to the control by using Pearson’s chi-square test. The inhibitors slightly decreased LDL binding (10–15% decrease) when HDL was present and markedly decreased HDL binding (20–47% decrease) when LDL was present. The decreased binding to both HDL and LDL may offer mechanistic insights into how the inhibitors decrease CE transfer between particles.

### Effects of inhibitors on CETP binding affinities to lipoproteins in the ternary complexes

CETP binding affinities were modified by the presence of LDL and HDL. By comparing the control samples from the above experiments on the binary and ternary CETP complexes (Fig. 4F), we observed that approximately 23% more HDL bound to CETP when LDL was present (increasing from 9.9% to 12.2%). Similarly, approximately 50% more LDL bound to CETP when HDL was present (increasing from 12.9% to 19.5%). These results demonstrated that the binding affinity at one distal end of CETP can influence the binding events at the opposite distal end, thus suggesting that the binding affinities of the two distal ends of CETP are not independent of each other.

CETP binding affinities were also modified by the presence of CETP inhibitors. The above experiments on binary CETP complexes showed that the presence of inhibitors increased the HDL binding percentage by up to 5-fold and increased the LDL binding percentage by up to 2-fold (Fig. 4F). However, the inhibitors did not increase the CETP binding affinity to a lipoprotein when one end of CETP already bound to a lipoprotein; in contrast, the presence of the inhibitors actually decreased the binding affinity. For example, when LDL was present, Torcetrapib decreased HDL binding to CETP by approximately 43% (from 12.2% to 6.9%). Similarly, when HDL was present, Torcetrapib decreased LDL binding to CETP by approximately 10% (from 19.5% to 17.5%); Dalcetrapib decreased HDL binding by approximately 20% (from 12.2% to 9.7%) and decreased LDL binding by approximately 11% (from 19.5% to 17.4%); and Anacetrapib decreased HDL binding by approximately 47% (from 12.2% to 6.5%) and decreased LDL binding by approximately 15% (from 19.5% to 16.6%) (Fig. 4F).

### CETP inhibitors decrease CE transfer from HDL to LDL

**Fig. 5.**
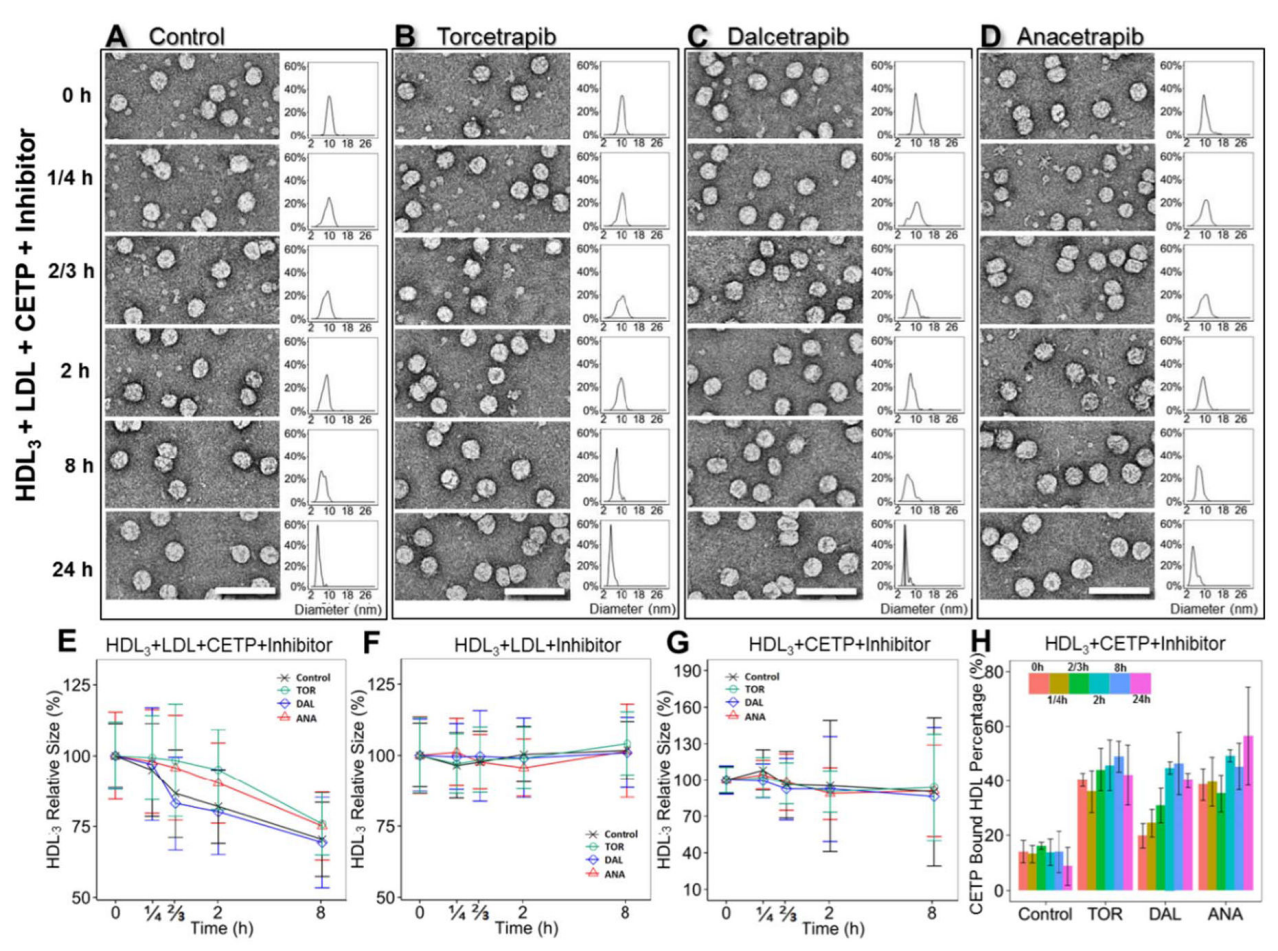
Effects of inhibitors on the CE transfer rate between plasma HDL_3_ and LDL, as shown by OpNS EM. Comparison of CETP lipid transfer activity and binding affinity during the incubation of human plasma HDL_3,_ LDL and CETP with or without inhibitors for 0 min, 15 min, 40 min, 2 h, 8 h and 24 h at 37 °C. OpNS EM images of the samples at representative incubation times are presented in the top left columns for A) the control group, B) Torcetrapib, C) Dalcetrapib, and D) Anacetrapib. The corresponding HDL size distributions are shown in top right columns. Quantitative diameter analysis of the HDL particles at 0 min, 15 min, 40 min, 2 h and 8 h for samples of E) a mixture of HDL, LDL, CETP and inhibitors; F) a mixture of HDL, LDL and inhibitors; and G) a mixture of HDL, CETP and inhibitors are shown in the bottom panel. A total of 300–500 HDL_3_ particles were assessed for each category. The particle diameters were measured on the basis of the geometric mean of two diameters: the longest diameter and its perpendicular diameter. Samples treated with control buffer, Torcetrapib, Dalcetrapib and Anacetrapib are represented by black, green, blue and orange lines, respectively. H) Histogram of the percentage of CETP-bound HDL against incubation times in the sample of HDL and CETP with inhibitors. Different incubation time periods are represented by different colors. All scale bars: 75 nm. The error bars in E, F, G and H are standard deviations.

To investigate how the CETP inhibitors affect CETP-mediated transfer of CE from HDL to LDL, the above samples (in which the molar ratio of CETP, HDL_3_ and LDL was 9:3:1) were incubated with or without inhibitors (approximately 10 μM) for 0 min, 15 min, 40 min, 2 h, 8 h and 24 h at 37 °C (Fig. 5A–D, Supplemental Fig. 21–24). The changes in HDL diameter reflected the transfer of CE from HDL to LDL. Additionally, as controls, samples of HDL_3_ incubated with LDL but without CETP (Supplemental Fig. 25–28) and samples of HDL_3_ incubated with CETP but without LDL (Supplemental Fig. 29–32) were prepared and examined under the conditions of incubation with or without inhibitors for the above time periods.

Prior to the study, these control experiments were performed to exclude the possibility that changes in HDL diameter were caused by CETP inhibitors directly inducing CE transfer between HDL and LDL. In this case, HDL_3_ and LDL at a molar ratio of 3:1 were incubated at the different time points stated above with or without inhibitors at physiological temperatures. There were no significant changes in the diameters of the HDL particles (all within approximately 10%) at any of the time points, regardless of whether the inhibitors were included in the incubations (Fig. 5F, Supplemental Fig. 25–28), thus suggesting that lipid transfer between HDL and LDL is not mediated by CETP inhibitors.

We additionally sought to exclude the possibility that CETP-associated HDL_3_ fusion and subsequent remodeling of the fusion product [23,47–49] might lead to a decrease in HDL particle size. To examine these scenarios, HDL_3_ and CETP at a molar ratio of 1:3 were incubated with or without inhibitors (Fig. 5G, Supplemental Fig. 29–32). Statistical analysis showed that the average HDL diameters remained similar (all within approximately 10% of one another) even after 24 h of incubation. However, the standard deviation of the particle diameter gradually increased. The sample without inhibitor had the largest standard deviation, whereas the samples with Torcetrapib and Anacetrapib had the smallest standard deviations (Fig. 5G). After 24 h of incubation, the particles with a major peak 6 nm in diameter and a minor peak at 50 nm were observed (Supplemental Fig. 29). In the presence of the CETP inhibitors, the processes of particle size distribution polarization were significantly slow down (Supplemental Fig. 30–32). It is unclear how Torcetrapib, but not anacetrapib or dalcetrapib, slow downed the process.

Statistical analysis showed that the presence of inhibitor increased the percentage of HDL particles bound to CETP over time, with Dalcetrapib causing the most rapid increase (Fig. 5H). This rapid increase may correlate with the time-dependence of the effects induced by Dalcetrapib [14]. Nevertheless, the increased binding percentage did not alter the average diameter of HDL.

Analyzing changes in HDL diameters in the ternary complexes after treatments with different inhibitors yielded the following observations: i) The average diameter of HDL remained similar (within approximately 5%) during the first 15 min (1/4 h) of incubation, regardless of which inhibitor was present. ii) After 40 min (2/3 h) of incubation, the average HDL diameter decreased by approximately 17% in the control and Dalcetrapib-incubated samples, whereas no changes were observed (within approximately 5%) with the Torcetrapib- and Anacetrapib-incubated samples. iii) After 2 h of incubation, the average HDL diameter decreased by approximately 21% in the control and Dalcetrapib-incubated samples, whereas it showed only a slight decrease (approximately 10%) with the other two inhibitors (Fig. 5A–E). iv) After 8 h of incubation, the average HDL diameter decreased by approximately 30% in the control and Dalcetrapib-incubated samples and by approximately 25% in the samples incubated with Torcetrapib and Anacetrapib (Fig. 5A–E). After 24 h of incubation, in conjunction with the HDL_3_ particles shrinking in size, HDL_3_ became barely visible in the micro-graphs (right panels in Fig. 5A–D), thereby preventing statistical analysis. The above results suggest another possible mechanism for inhibitor effects via reducing the CETP binding affinity to HDL and LDL when both lipoproteins are present. Notably, Torcetrapib and Anacetrapib had relatively higher efficacy in preventing CE transfer than Dalcetrapib within 2 h of incubation. However, after 8 h, all HDL diameters decreased to values similar to those observed when the inhibitors were absent. These time-dependent efficiencies suggest that the inhibitors may bind reversibly to CETP (Fig. 5E).

Our EM studies show that Dalcetrapib has a weaker binding affinity for HDL and a lower CETP inhibition efficiency than Torcetrapib and Anacetrapib. This is consistent with what has been observed using traditional biochemical approaches [14] in which the same CETP inhibitors were incubated with HDL and LDL under comparable conditions to those of the present study. The results from the study of Ranalletta et al. established that, i) Anacetrapib and Torcetrapib inhibit CETP-mediated CE and TG transfers with similar potencies, which is similar to our EM result shown in Fig. 5; ii) Dalcetrapib is a significantly less potent CETP inhibitor than Anacetrapib or Torcetrapib, which is consistent with our EM data in Fig. 5; and iii) all of the CETP inhibitors induced tight binding of CETP to HDL, which leads to inhibition of CETP activity [14], which is also consistent with our EM data in Fig. 4F. However, the study of Ranalletta et al. was not designed to determine how CETP interacts with HDL and LDL at the single molecule level, or to ascertain whether CETP inhibition induces conformational changes in the CETP molecule that regulate its interaction with lipoproteins. Our EM images showed that the inhibitors do not change the conformation of CETP or its interaction with HDL; rather, our results show that they reduce the ratio of ternary complex formation, demonstrating a new mechanism of CETP inhibition.

## 3. Discussion

### CETP mechanisms in CE transfer

**Fig. 6.**
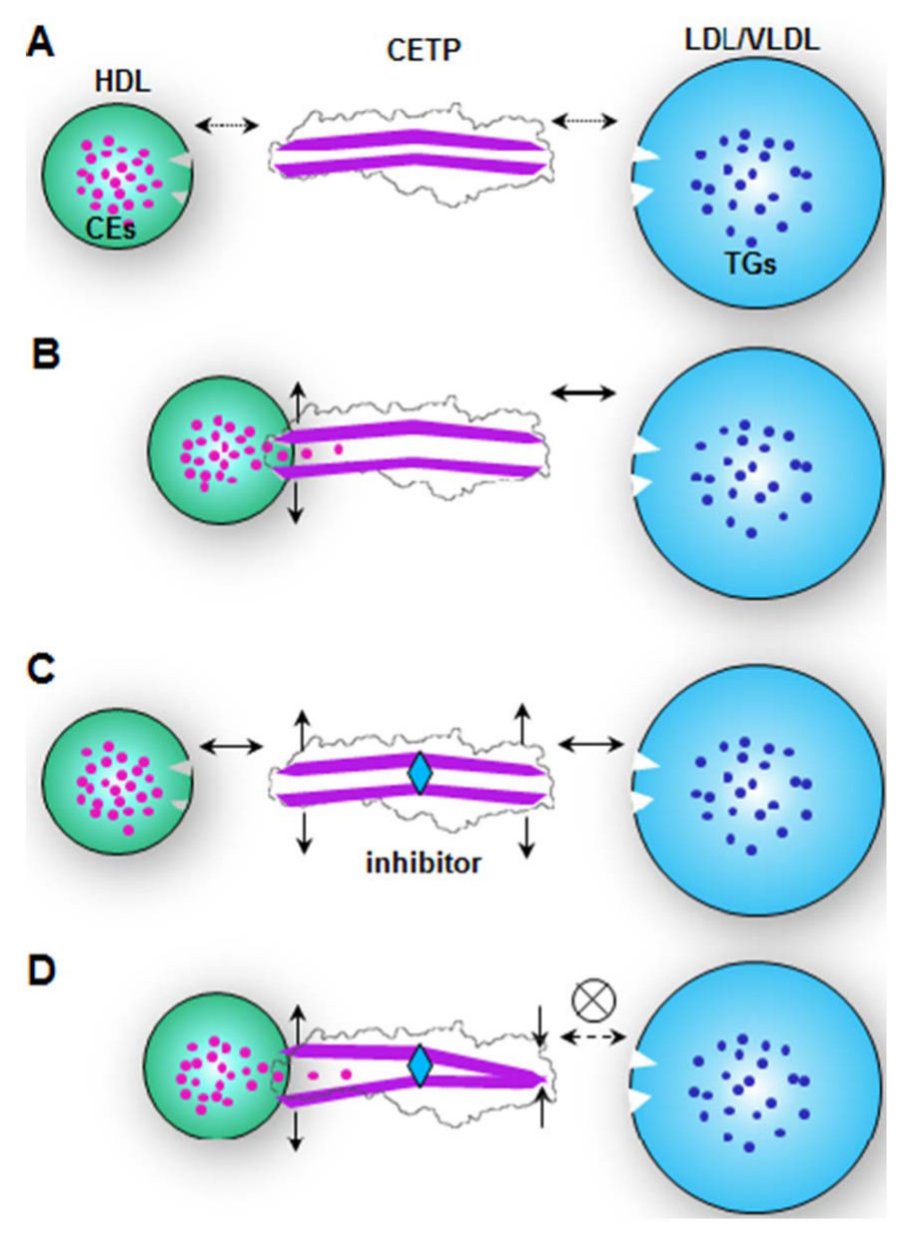
A hypothesis for the CETP inhibitor mechanism. A) Lipoproteins (including HDL, LDL and VLDL) have intermediate binding affinities to the CETP when CETP is in a near “closed” confirmation in solution. B) While one distal end of CETP interacts with one class of lipoproteins, such as HDL, the CEs of HDL are then taken up into CETP, and they produce a conformational change, increasing the binding affinity to other classes of lipoproteins, such as LDL or VLDL. C) The CETP inhibitor bound to the middle portion of the CETP triggers conformational changes at both distal ends, thereby increasing their binding affinities to both classes of lipoproteins. D) However, after neutral lipids, such as CEs, are taken up into one distal end, a conformational change is triggered at the opposite end thus decreasing the binding affinity to other classes of lipoproteins.

The statistical analyses of ternary complexes showed that the CETP binding on HDL and LDL increased by ~20% and 50% respectively when CETP was bound to two species of lipoproteins simultaneously (Fig. 4F, control row). This phenomenon suggests that either one of the CETP distal ends bound to a lipoprotein triggers a conformational change on the other distal end, thus resulting in an enhanced binding affinity to other species of lipoproteins (Fig. 6A–C), which increase CE transfer activity. The correlation of two distal ends on CETP has rarely been studied previously, possibly because the crystal structure of CETP with or without inhibitors does not show a significant conformational change [11,39]. However, given that the same hole-crystals were used for both structures and the crystal lattice may constrain the conformational change of the structures, a local conformational change of CETP is still possible. Recent all-atom and coarse-grained MD simulations showed a high degree of conformational flexibility of the protein in solution [28,50], especially in the β-barrel domains [27,28]. This conformational flexibility may increase the length of the center cavity in forming a channel [27,28] through which the central containing CEs and TGs can be transferred directionally [28]. The merging activity of CETP distal ends into a lipid layer produces distinct differences from CETP in solution [51]. The N-terminal β-barrel domain may even open a pore to allow uptake of the CE molecule from the HDL core [29].

Considering that VLDL particles are more labile and easier to be damaged than LDL during sample preparation and incubation, they contaminate the background and make identifying and measuring CETPs and HDLs difficult [41]. Thus, we only incubate the CETP with VLDL for a short time (such as 1 min). The EM images showed that CETP penetrates into the VLDL surface and mediates CE transfer via a tunnel mechanism, which is consistent with our early report [41]. TG transfer was not investigated in that study. However, as more CETP molecules can bind to VLDL than to LDL, the binding mechanism of CETP to the two lipoproteins is probably different. This is consistent with cryo-EM IPET 3D reconstructions of VLDL alone and VLDL-antibody complexes in which VLDL has a polyhedral surface, which is distinct from LDL [17]. However, a detailed investigation of this point is beyond the scope of the current project.

Our observation of ternary complexes of HDL-CETP-LDL favors the “tunnel mechanism” [25,52]. However, some other studies have yielded different opinions about the CETP mechanism. García-González et al. have discovered that small peptides derived from CETP cause a mixture of phosphatidylcholine/CE aggregates forming ~6 nm micelle-like particles. As a result, authors suggested that similar mechanism of CE transfer from HDL to LDL can be adopted by the full length CETP [15]. In our previous [25] and current study, we neither observe those 6 nm micelle-like small particles after incubating CETP and HDL, nor explains how those protein-free micelle-like particles could function in sensing, targeting, and binding to LDL/VLDL, for directionally delivering their contained CEs. Notably, a recent EM study by Lauer et al. has also reported that CE transfer does not require a ternary tunnel complex with CETP, on the basis of negative evidence; i.e., the absence of observation of the existence of the binary complexes of CETP-LDL or ternary complexes of HDL-CETP-LDL [54]. However, this study also did not consider how CEs are transferred from HDL to LDL in the absence of CETP interaction with LDL nor show any new observed transportation media, such as the micelle-like particles as predicted by García-González et al. [15]. Our previous and current results show the existence of the binary complex of CETP-LDL, and early biochemistry experiments have also suggested the CETP-interaction with LDL. For example, Morton et al. have shown that LDL and VLDL-Sepharose columns release 50% of CETP by 45 min and 15 min, respectively [55]. The slower release speed of CETP from LDL compared with VLDL suggests an interaction between CETP and LDL [55].

### Insights into the CETP inhibitor mechanism

An interesting result from our study is the direct observation of significant inhibitors induced increment of the CETP binding to HDL (from ~9.9% to ~50.5%), which is in agreement with results from an early study [56]. Same as HDL, inhibitors also clearly increased CETP binding to LDL (from ~12.9% to ~21.8%). Unexpectedly, this increased CETP binding to each class of lipoproteins (HDL or LDL, in forming binary complexes) did not contribute to increasing the CETP binding to both classes of lipoproteins (forming ternary complexes) but instead decreased ternary complex formation (Fig. 4F). This is an interesting effect of inhibitors since it reversed a possible allosteric effect that either one of the CETP distal ends bound to a lipoprotein may trigger a conformational change at the other distal end. Based on these EM result statistical analysis, a hypothesis is proposed to explain the effect of inhibitor in CETP binding. In brief, the inhibition mechanism may be described as a “seesaw” model (Fig. 6). The natural substrate binding on one side of the CETP while initiating lipid transfer can cause a corresponding change on the other side. The CETP seesaw is in a “balanced state”, where both sides of binding are enhanced. When inhibitor is introduced into the CETP tunnel without lipoprotein substrate binding, the CETP is again in an enhanced “balanced state” as inhibitor might play a role mimicking the lipid transfer state. However if both constraints were added (Fig. 6D), the seesaw become “tilted” causing a decreased CETP binding on the other side.

Recent MD simulations indirectly support this hypothesis through the observation that hydrophobic interactions between the CETP core tunnel residues and inhibitors increase the plasticity of CETP [50], especially in its N-terminal β-barrel domain distal end [32] and C-terminal β-barrel domain distal end [42]. However, the hypothesis remains to be validated in the future by other orthogonal techniques, such as surface plasmon resonance analysis of binding interactions, fluorescence resonance energy transfer microscopy imaging, high resolution fluorescence labelled imaging, or even using high resolution cryo-EM equipped with a direct detector to directly observe the proposed conformational change. Despite of the hypothesis, the findings that inhibitors decreased the formation of ternary complexes; i.e., HDL-CETPLDL, provide additional knowledge beyond the general understanding of the mechanism of CETP inhibitors; i.e., inhibitors block the central cavity of CETP and thereby prevent CE transfer [11,14].

### Inhibitor efficiencies

Our experiments revealed the existence of the ternary complex by using human plasma HDL_3_, CETP and LDL; these results were consistent with findings from studies using recombinant apo-AI HDL [25]. We found that the inhibitors decreased the tendency of CETP to form a ternary complex. Torcetrapib and Anacetrapib had a similar degree of efficiency, which was higher than that of Dalcetrapib, in decreasing the ratio of the CETP ternary complex (Fig. 4F). These efficiencies appeared to be consistent with their corresponding degrees of lipid-altering efficacies observed in large clinical trials. Clinical statistics show that Torcetrapib significantly increases HDL-C (~70%) and decreases LDL-C (~25%) [5]. Anacetrapib showed a similar or higher efficiency than Torcetrapib; Anacetrapib boosted HDL-C by approximately 138% and diminished LDL-C by ~36% [7,57]. Dalcetrapib showed the least efficiency, raising HDL-C by only ~30% and modestly decreasing LDL-C by ~6% [58]. The consistency of inhibitor efficiencies between our experiments and these larger clinical results suggests that our approach may be used as a low-cost and high-efficiency tool to evaluate an inhibitor before clinical trials.

### A potential effect of current inhibitors

Although Torcetrapib showed a higher efficiency of CETP inhibition, large clinical trials have been halted because of side effects on blood pressure and/or electrolyte imbalance [5,59]. In contrast, the Dalcetrapib clinical trial was ceased because of a lack of therapeutic efficacy. We suspect that the side effects of Torcetrapib may be related to the significant increase in CETP-HDL binary complexes (increased 5-fold). The tight binding between HDLCETP may result in Torcetrapib interfering in the normal metabolism of HDL. A relatively low binding rate, such as the 29.2% for Dalcetrapib and the 43.1% for Anacetrapib, may decrease this risk and prevent potential side effects. Earlier studies have shown that Torcetrapib and Anacetrapib cause more CETP molecules to bind to HDL than does Dalcetrapib [14]; these tightly bound inhibitor-CETPs can be taken up by cells during the HDL endocytosis process [60]. Anacetrapib has no reported side effects, and has recently been reported to reduce major cardiovascular events. Dalcetrapib has a lower efficiency with decreased HDL-CETP-LDL ternary complex formation (Fig. 4F), but it still increases HDL-C levels by ~30% without obvious side effects [58]. The lack of therapeutic efficacy of CETP inhibitors in clinical trials suggests an elevated HDL-C level in isolation may not result in atherprotection. However, it is worthy to investigate whether the development of new next-generation CETP inhibitors that decrease the rate of binary as well as ternary complexes between HDL, LDL and CETP, as opposed the current inhibitors that only inhibit ternary complex formation, is more efficacious in terms of atheroprotection. Nevertheless, we propose a new next-generation CETP inhibitor that should decrease the rate of formation of ternary complexes of HDL-CETP-LDL to prevent the HDL-C was transferred to LDL-C, while also decreasing the formation of binary complexes of CETP-HDL and CETP-LDL to avoid the inhibitor caused side fact via inhibitor involved in the regular HDL or LDL metabolism.

In summary, we believe that our EM approach may aid in examination of the mechanism and efficiency of inhibitors at the molecular level to treat CVD.

## 4. Experimental procedures

### Protein and lipoprotein isolation

The recombinant human CETP mutant N341Q (approximately 53 kDa) was expressed and purified as previously described [14]. This version of CETP has a mutation at a single glycosylation site to achieve better yields during protein purification and a more uniform glycosylation pattern. The mutated CETP behaves identically to WT CETP in lipid transfer assays [38]. The concentration of the purified CETP was approximately 2.3 mg/ml by absorbance at 280 nm. Native plasma HDL_3_ was isolated from fresh human plasma through ultracentrifugation as previously described [61]; it contained 4.28 mg/ml protein, 2.39 mg/ml CE and 1.03 mg/ml TG. LDL (d = 1.006–1.069 g/ml, apoB 64.9 mg/dL) and VLDL (d < 1.006 g/ml, apoB 24.5 mg/dL) were isolated in the Krauss laboratory by sequential flotation of plasma from fasting, healthy male volunteers and further purified by ultracentrifugation [62]. Torcetrapib, Dalcetrapib and Anacetrapib were synthesized and prepared by the Merck Medicinal Chemistry Department (Rahway, NJ) with > 99% purity, as determined by high-performance liquid chromatography-mass spectrometry (HPLC-MS) and nuclear magnetic resonance (NMR) spectroscopy.

### Cryo-EM specimen preparation and data collection

CETP and plasma HDL_3_ were incubated at a molar ratio of 3:1 in their original buffer for 5 min at 37 °C. After dilution of the incubation solution 5 times with Dulbecco’s phosphate buffered saline (DPBS), an aliquot (approximately 3 μl diluted solution) was applied to a glow-discharged holey carbon film coated 200-mesh copper grid (Cu-200HC, Pacific Grid-Tech, San Francisco, CA) for 5 s. After being blotted with filter paper (Whatman® qualitative filter paper, Grade 1, Maidstone, UK) on one side for 3 s, the samples were then flash-frozen in liquid ethane under conditions of 100% humidity at 8 °C with a Leica rapid-plunging device. Cryo-EM micrographs of the sample were acquired under a defocus of < 2 μm with a Gatan Ultrascan high-sensitivity 4 K × 4 K CCD camera operated at 80 K magnification by the Zeiss Libra 120 TEM (each pixel of the micrograph corresponded to 2.4 Å in the specimens).

### Three-dimensional reconstruction of the HDL-CETP complex

The defocus of each micrograph was determined by fitting the contrast transfer function (CTF) parameters with its power spectrum by using *ctffind3* in the FREALIGN software package [63]. The phase of each micrograph was corrected by a Wiener filter with the SPIDER software package [64]. First, 24,000 isolated CETP-HDL complex particles from the cryo-EM images were initially selected and windowed as 192 × 192 pixel images using the *e2boxer.py* program in EMAN2 [65]. Then, a subpopulation of approximately 3200 particles with homogeneous HDL size and binding CETPs were selected for structure analysis. Approximately 200 class averages were generated by reference-free class averages computed using *refind2d.py* in EMAN [66]. To prevent bias from a starting model in 3D reconstruction and refinement, a featureless model with a smooth solid cylinder (length approximately 75 Å, diameter approximately 30 Å) attached to a featureless solid sphere (diameter 100 Å) was used as the initial starting model, as a generally used strategy for 3D refinement [67]. In the first four rounds of refinement, only very-low-resolution particle information was used, and iterative refinement was used for convergence. Then, CTF amplitude and phase corrections, finer angular sampling and solvent flattening via masking were sequentially applied for iteration to convergence. According to the 0.5 Fourier shell correlation criterion [68], the final resolution of the asymmetric reconstruction of the CETP-HDL complex was 28 Å (Fig. 2H).

### Negative stained EM specimen preparation

Specimens were prepared for EM with a previously described optimized negative staining (OpNS) protocol [34], which effectively minimizes the formation of rouleaux artifacts from lipoproteins [19,41]. In brief, CETP (2.3 mg/ml) was pre-incubated with Anacetrapib, Dalcetrapib, Torcetrapib or drug solvent buffer (as a control) separately at 37 °C for 1 h; each drug was used at an approximately 100 μM concentration. To examine the interaction of CETP with different lipoproteins under different drug treatment conditions, the above pre-incubation solutions were then incubated for 1 min with HDL_3_ at a molar ratio of approximately 3:1 (CETP:HDL_3_), with LDL/VLDL at a molar ratio of approximately 9:1 (CETP:LDL/VLDL) or with an HDL_3_-LDL mixture at a molar ratio of approximately 9:3:1 (CETP:HDL_3_:LDL) at 37 °C. The final drug concentrations were all approximately 10 μM in the final incubation solution for a maximal inhibitory effect [14]. To study lipid transfer among lipoproteins under different CETP inhibitor treatment conditions, portions of the above samples, as well as of additional control samples (including HDL-LDL-inhibitors, HDL-CETP-LDL-inhibitors and HDL-CETP-inhibitors), were incubated at 37 °C for 0 min, 15 min, 40 min, 2 h, 8 h, and 24 h before preparation for EM.

The EM specimens were prepared by following our optimized negative-staining protocol (OpNS) for examining lipoproteins. In brief, approximately 3 μl of each sample was diluted 100-fold with Dulbecco’s phosphate-buffered saline (DPBS) and was quickly placed on a thin, carbon-coated 200 mesh copper grid (CF200-Cu-SP, thin carbon film from Electron Microscopy Science) that had been glow-discharged. After 1 min, excess solution was blotted with filter paper. The sample was then washed rapidly with water and stained (1% uranyl formate, UF) [33,34,41]. After being air-dried under nitrogen, the specimens were further dried at room temperature overnight prior to use.

### Electron microscopy data acquisition and image preprocessing

Micrographs were acquired under a defocus of approximately 0.6 μm and a magnification of 80 k on a Gatan UltraScan 4 K × 4 K CCD attached to a Zeiss Libra 120 Plus transmission electron microscope (Carl Zeiss NTS GmbH, Oberkochen, Germany), which was operated under high tension at 120 kV using 20 eV energy filtering. Each micrograph pixel corresponded to 1.48 Å. A total of 4–8 micro-graphs were acquired for each condition. The contrast transfer function (CTF) of each micrograph was determined and then corrected using the phase-flip option included in *ctfit* (EMAN software package) [66]. All isolated particles in a micrograph were windowed using the *boxer* software by EMAN. Gaussian low-pass filters were applied to these particle images selected and windowed from each incubation condition before statistical analyses.

### Statistical analyses of CETP binding to lipoprotein particles

To harvest a sufficient number of isolated lipoprotein/CETP particles for statistical analysis, 4–5 images (containing 300–500 particles) were collected from each sample at the times stated above. For each lipoprotein particle, the number of bound CETPs was counted by accumulating the number of observed rod-shaped protrusions on the edge of the spherical lipoprotein. This number varied slightly depending on inclusion of the undetectable CETPs that were located behind and in front of the lipo-protein particles. As we have previously calculated [26], the probability (℘) (i.e., the ratio of the CETP visible area vs. the overall sphere area) is $℘=\cos\left[sin^{-1}\left(\frac{d}{d+2l}\right)\right]$, where *d* is the lipoprotein diameter, and *l* is the CETP protrusion length.

The particle diameter was determined by measuring diameters in two orthogonal directions, as previously described [19]. In brief, the geometric mean of the perpendicular diameters was used to represent the particle diameter. Histograms of the particle diameters were generated with 0.5 nm sampling steps. Each histogram was fitted with a 6th degree polynomial function in *R* software for data analysis. Pearson’s chi-square test with Yates’ continuity correction was used to assess differences between the datasets. Statistical significance was defined as *p* < 0.05.

## Supplementary Material

supporting

## Abbreviations

CETP: cholesteryl ester transfer protein
CE: cholesterol ester
EM: electron microscopy
cryo: EM cryo-electron microscopy
HDL: high-density lipoprotein
LDL: low-density lipoprotein
OpNS: optimized negative-staining
VLDL: very low-density lipoprotein

## References

[1] Barter PJ, Brewer HB Jr., Chapman MJ, Hennekens CH, Rader DJ, Tall AR, Cholesteryl ester transfer protein: a novel target for raising HDL and inhibiting atherosclerosis, Arterioscler. Thromb. Vasc. Biol 23 (2003) 160–167. 12588754 10.1161/01.atv.0000054658.91146.64

[2] Vergeer M, Holleboom AG, Kastelein JJ, Kuivenhoven JA, The HDL hypothesis: does high-density lipoprotein protect from atherosclerosis? J. Lipid Res 51 (2010) 2058–2073. 20371550 10.1194/jlr.R001610 PMC2903818

[3] Inazu A, Brown ML, Hesler CB, Agellon LB, Koizumi J, Takata K, Maruhama Y, Mabuchi H, Tall AR, Increased high-density lipoprotein levels caused by a common cholesteryl-ester transfer protein gene mutation, N. Engl. J. Med 323 (1990) 1234–1238. 2215607 10.1056/NEJM199011013231803

[4] Brown ML, Inazu A, Hesler CB, Agellon LB, Mann C, Whitlock ME, Marcel YL, Milne RW, Koizumi J, Mabuchi H, , Molecular basis of lipid transfer protein deficiency in a family with increased high-density lipoproteins, Nature 342 (1989) 448–451. 2586614 10.1038/342448a0

[5] Barter PJ, Caulfield M, Eriksson M, Grundy SM, Kastelein JJ, Komajda M, Lopez-Sendon J, Mosca L, Tardif JC, Waters DD, Shear CL, Revkin JH, Buhr KA, Fisher MR, Tall AR, Brewer B, Investigators I, Effects of torcetrapib in patients at high risk for coronary events, N. Engl. J. Med 357 (2007) 2109–2122. 17984165 10.1056/NEJMoa0706628

[6] Derks M, Anzures-Cabrera J, Turnbull L, Phelan M, Safety, tolerability and pharmacokinetics of dalcetrapib following single and multiple ascending doses in healthy subjects: a randomized, double-blind, placebo-controlled, phase I study, Clin. Drug Investig. 31 (2011) 325–335. 10.1007/BF03256931 21366361

[7] Gotto AM Jr., Cannon CP, Li XS, Vaidya S, Kher U, Brinton EA, Davidson M, Moon JE, Shah S, Dansky HM, Mitchel Y, Barter P, D. Investigators, Evaluation of lipids, drug concentration, and safety parameters following cessation of treatment with the cholesteryl ester transfer protein inhibitor anacetrapib in patients with or at high risk for coronary heart disease, Am. J. Cardiol 113 (2014) 76–83. 24188894 10.1016/j.amjcard.2013.08.041

[8] Friedrich S, Kastelein JJ, James D, Waterhouse T, Nissen SE, Nicholls SJ, Krueger KA, The pharmacokinetics and pharmacokinetic/pharmacodynamic relationships of evacetrapib administered as monotherapy or in combination with statins, CPT Pharmacometrics Syst. Pharmacol 3 (2014) e94. 24452615 10.1038/psp.2013.70 PMC3910017

[9] Hovingh GK, Kastelein JJ, van Deventer SJ, Round P, Ford J, Saleheen D, Rader DJ, Brewer HB, Barter PJ, Cholesterol ester transfer protein inhibition by TA-8995 in patients with mild dyslipidaemia (TULIP): a randomised, double-blind, placebo-controlled phase 2 trial, Lancet 386 (2015) 452–460. 26047975 10.1016/S0140-6736(15)60158-1

[10] Drayna D, Jarnagin AS, McLean J, Henzel W, Kohr W, Fielding C, Lawn R, Cloning and sequencing of human cholesteryl ester transfer protein cDNA, Nature 327 (1987) 632–634. 3600759 10.1038/327632a0

[11] Qiu X, Mistry A, Ammirati MJ, Chrunyk BA, Clark RW, Cong Y, Culp JS, Danley DE, Freeman TB, Geoghegan KF, Griffor MC, Hawrylik SJ, Hayward CM, Hensley P, Hoth LR, Karam GA, Lira ME, Lloyd DB, McGrath KM, Stutzman-Engwall KJ, Subashi AK, Subashi TA, Thompson JF, Wang IK, Zhao H, Seddon AP, Crystal structure of cholesteryl ester transfer protein reveals a long tunnel and four bound lipid molecules, Nat. Struct. Mol. Biol 14 (2007) 106–113. 17237796 10.1038/nsmb1197

[12] Bolanos-Garcia VM, Soriano-Garcia M, Mas-Oliva J, CETP and exchangeable apoproteins: common features in lipid binding activity, Mol. Cell. Biochem 175 (1997) 1–10. 9350027 10.1023/a:1006887729274

[13] Xicohtencatl-Cortes J, Castillo R, Mas-Oliva J, In search of new structural states of exchangeable apolipoproteins, Biochem. Biophys. Res. Commun 324 (2004) 467–470. 15474451 10.1016/j.bbrc.2004.09.045

[14] Ranalletta M, Bierilo KK, Chen Y, Milot D, Chen Q, Tung E, Houde C, Elowe NH, Garcia-Calvo M, Porter G, Eveland S, Frantz-Wattley B, Kavana M, Addona G, Sinclair P, Sparrow C, O’Neill EA, Koblan KS, Sitlani A, Hubbard B, Fisher TS, Biochemical characterization of cholesteryl ester transfer protein inhibitors, J. Lipid Res 51 (2010) 2739–2752. 20458119 10.1194/jlr.M007468 PMC2918456

[15] Garcia-Gonzalez V, Gutierrez-Quintanar N, Mendoza-Espinosa P, Brocos P, Pineiro A, Mas-Oliva J, Key structural arrangements at the C-terminus domain of CETP suggest a potential mechanism for lipid-transfer activity, J. Struct. Biol 186 (2014) 19–27. 24530617 10.1016/j.jsb.2014.02.002

[16] Kono M, Okumura Y, Tanaka M, Nguyen D, Dhanasekaran P, Lund-Katz S, Phillips MC, Saito H, Conformational flexibility of the N-terminal domain of apolipoprotein a-I bound to spherical lipid particles, Biochemistry 47 (2008) 11340–11347. 18831538 10.1021/bi801503r PMC2667695

[17] Yu Y, Kuang YL, Lei D, Zhai X, Zhang M, Krauss RM, Ren G, Polyhedral 3D structure of human plasma very low density lipoproteins by individual particle cryo-electron tomography1, J. Lipid Res 57 (2016) 1879–1888. 27538822 10.1194/jlr.M070375 PMC5036368

[18] Ren G, Rudenko G, Ludtke SJ, Deisenhofer J, Chiu W, Pownall HJ, Model of human low-density lipoprotein and bound receptor based on cryoEM, Proc. Natl. Acad. Sci. U. S. A 107 (2010) 1059–1064. 20080547 10.1073/pnas.0908004107 PMC2798884

[19] Zhang L, Song J, Cavigiolio G, Ishida BY, Zhang S, Kane JP, Weisgraber KH, Oda MN, Rye KA, Pownall HJ, Ren G, Morphology and structure of lipoproteins revealed by an optimized negative-staining protocol of electron microscopy, J. Lipid Res 52 (2011) 175–184. 20978167 10.1194/jlr.D010959 PMC2999936

[20] Barter PJ, Jones ME, Kinetic studies of the transfer of esterified cholesterol between human plasma low and high density lipoproteins, J. Lipid Res 21 (1980) 238–249. 7373163

[21] Ginsburg BE, Zetterstrom R, High density lipoprotein concentrations in newborn infants, Acta Paediatr. Scand 66 (1977) 39–41. 188304 10.1111/j.1651-2227.1977.tb07804.x

[22] Ferretti G, Bacchetti T, Negre-Salvayre A, Salvayre R, Dousset N, Curatola G, Structural modifications of HDL and functional consequences, Atherosclerosis 184 (2006) 1–7. 16157342 10.1016/j.atherosclerosis.2005.08.008

[23] Lagrost L, Gambert P, Dangremont V, Athias A, Lallemant C, Role of cholesteryl ester transfer protein (CETP) in the HDL conversion process as evidenced by using anti-CETP monoclonal antibodies, J. Lipid Res 31 (1990) 1569–1575. 2246610

[24] Lund-Katz S, Liu L, Thuahnai ST, Phillips MC, High density lipoprotein structure, Front. Biosci 8 (2003) d1044–1054. 12700101 10.2741/1077

[25] Zhang L, Yan F, Zhang S, Lei D, Charles MA, Cavigiolio G, Oda M, Krauss RM, Weisgraber KH, Rye KA, Pownall HJ, Qiu X, Ren G, Structural basis of transfer between lipoproteins by cholesteryl ester transfer protein, Nat. Chem. Biol 8 (2012) 342–349. 22344176 10.1038/nchembio.796 PMC3792710

[26] Zhang M, Charles R, Tong H, Zhang L, Patel M, Wang F, Rames MJ, Ren A, Rye KA, Qiu X, Johns DG, Charles MA, Ren G, HDL surface lipids mediate CETP binding as revealed by electron microscopy and molecular dynamics simulation, Sci Rep 5 (2015) 8741. 25737239 10.1038/srep08741 PMC4348656

[27] Lei D, Zhang X, Jiang S, Cai Z, Rames MJ, Zhang L, Ren G, Zhang S, Structural features of cholesteryl ester transfer protein: a molecular dynamics simulation study, Proteins 81 (2013) 415–425. 23042613 10.1002/prot.24200 PMC3557553

[28] Chirasani VR, Revanasiddappa PD, Senapati S, Structural plasticity of cholesteryl ester transfer protein assists the lipid transfer activity, J. Biol. Chem 291 (2016) 19462–19473. 27445332 10.1074/jbc.M116.744623 PMC5016684

[29] Cilpa-Karhu G, Jauhiainen M, Riekkola ML, Atomistic MD simulation reveals the mechanism by which CETP penetrates into HDL enabling lipid transfer from HDL to CETP, J. Lipid Res 56 (2015) 98–108. 25424006 10.1194/jlr.M054288 PMC4274075

[30] Lei D, Rames M, Zhang X, Zhang L, Zhang S, Ren G, Insights into the tunnel mechanism of cholesteryl ester transfer protein through all-atom molecular dynamics simulations, J. Biol. Chem 291 (2016) 11. 27143480 10.1074/jbc.M116.715565 PMC4933163

[31] Koivuniemi A, Vuorela T, Kovanen PT, Vattulainen I, Hyvonen MT, Lipid exchange mechanism of the cholesteryl ester transfer protein clarified by atomistic and coarse-grained simulations, PLoS Comput. Biol 8 (2012) e1002299. 22253581 10.1371/journal.pcbi.1002299 PMC3257282

[32] Aijanen T, Koivuniemi A, Javanainen M, Rissanen S, Rog T, Vattulainen I, How anacetrapib inhibits the activity of the cholesteryl ester transfer protein? Perspective through atomistic simulations, PLoS Comput. Biol 10 (2014) e1003987. 25412509 10.1371/journal.pcbi.1003987 PMC4238956

[33] Zhang L, Song J, Newhouse Y, Zhang S, Weisgraber KH, Ren G, An optimized negative-staining protocol of electron microscopy for apoE4 POPC lipoprotein, J. Lipid Res 51 (2010) 1228–1236. 19965615 10.1194/jlr.D002493 PMC2853450

[34] Rames M, Yu Y, Ren G, Optimized negative staining: a high-throughput protocol for examining small and asymmetric protein structure by electron microscopy, J. Vis. Exp 90 (2014) e510871–15. 10.3791/51087 PMC4710468 25145703

[35] Ohi M, Li Y, Cheng Y, Walz T, Negative staining and image classification -powerful tools in modern electron microscopy, Biol. Proced. Online 6 (2004) 23–34. 15103397 10.1251/bpo70 PMC389902

[36] Zhang L, Lei D, Smith JM, Zhang M, Tong H, Zhang X, Lu Z, Liu J, Alivisatos AP, Ren G, Three-dimensional structural dynamics and fluctuations of DNA-nanogold conjugates by individual-particle electron tomography, Nat. Commun 7 (2016) 11083. 27025159 10.1038/ncomms11083 PMC4820932

[37] Tong H, Zhang L, Kaspar A, Rames MJ, Huang L, Woodnutt G, Ren G, Peptide-conjugation induced conformational changes in human IgG1 observed by optimized negative-staining and individual-particle electron tomography, Sci Rep 3 (2013) 1089. 23346347 10.1038/srep01089 PMC3549606

[38] Stevenson SC, Wang S, Deng L, Tall AR, Human plasma cholesteryl ester transfer protein consists of a mixture of two forms reflecting variable glycosylation at asparagine 341, Biochemistry 32 (1993) 5121–5126. 8494888 10.1021/bi00070a021

[39] Liu S, Mistry A, Reynolds JM, Lloyd DB, Griffor MC, Perry DA, Ruggeri RB, Clark RW, Qiu X, Crystal structures of cholesteryl ester transfer protein in complex with inhibitors, J. Biol. Chem 287 (2012) 37321–37329. 22961980 10.1074/jbc.M112.380063 PMC3481329

[40] van Antwerpen R, La Belle M, Navratilova E, Krauss RM, Structural heterogeneity of apoB-containing serum lipoproteins visualized using cryo-electron microscopy, J. Lipid Res 40 (1999) 1827–1836. 10508202

[41] Zhang L, Tong H, Garewal M, Ren G, Optimized negative-staining electron microscopy for lipoprotein studies, Biochim. Biophys. Acta 1830 (2013) 2150–2159. 23032862 10.1016/j.bbagen.2012.09.016 PMC3508368

[42] Jamalan M, Zeinali M, Ghaffari MA, A molecular dynamics investigation on the inhibition mechanism of cholesteryl ester transfer protein by Anacetrapib, Med. Chem. Res 25 (2016) 62–69.

[43] Barter P, Rye KA, Cholesteryl ester transfer protein inhibition to reduce cardiovascular risk: where are we now? Trends Pharmacol. Sci 32 (2011) 694–699. 22088767 10.1016/j.tips.2011.07.004

[44] Chirasani VR, Sankar R, Senapati S, Mechanism of inhibition of cholesteryl ester transfer protein by small molecule inhibitors, J. Phys. Chem. B 120 (2016) 8254–8263. 27111423 10.1021/acs.jpcb.6b01928

[45] Lauer ME, Graff-Meyer A, Rufer AC, Maugeais C, von der Mark E, Matile H, D’Arcy B, Magg C, Ringler P, Müller SA, Scherer S, Dernick G, Thoma R, Hennig M, Niesor EJ, Stahlberg H, Cholesteryl ester transfer between lipoproteins does not require a ternary tunnel complex with the cholesteryl ester transfer protein, J. Struct. Biol 194 (2016) 191–198. 26876146 10.1016/j.jsb.2016.02.016

[46] Ko KW, Ohnishi T, Yokoyama S, Triglyceride transfer is required for net cholesteryl ester transfer between lipoproteins in plasma by lipid transfer protein. Evidence for a hetero-exchange transfer mechanism demonstrated by using novel monoclonal antibodies, J. Biol. Chem 269 (1994) 28206–28213. 7961758

[47] Cavigiolio G, Shao B, Geier EG, Ren G, Heinecke JW, Oda MN, The interplay between size, morphology, stability, and functionality of high-density lipoprotein subclasses, Biochemistry 47 (2008) 4770–4779. 18366184 10.1021/bi7023354 PMC2902722

[48] Liang HQ, Rye KA, Barter PJ, Dissociation of lipid-free apolipoprotein A-I from high density lipoproteins, J. Lipid Res 35 (1994) 1187–1199. 7964180

[49] Rye KA, Hime NJ, Barter PJ, Evidence that cholesteryl ester transfer protein-mediated reductions in reconstituted high density lipoprotein size involve particle fusion, J. Biol. Chem 272 (1997) 3953–3960. 9020099 10.1074/jbc.272.7.3953

[50] Chirasani VR, Sankar R, Senapati S, Mechanism of inhibition of cholesteryl ester transfer protein by small molecule inhibitors, J. Phys. Chem. B 120 (2016) 8254–8263. 27111423 10.1021/acs.jpcb.6b01928

[51] Lei DS, Rames M, Zhang X, Zhang L, Zhang SL, Ren G, Insights into the tunnel mechanism of cholesteryl ester transfer protein through all-atom molecular dynamics simulations, J. Biol. Chem 291 (2016) 14034–14044. 27143480 10.1074/jbc.M116.715565 PMC4933163

[52] Tall AR, Plasma cholesteryl ester transfer protein, J. Lipid Res. 34 (1993) 1255–1274. 8409761

[54] Lauer ME, Graff-Meyer A, Rufer AC, Maugeais C, von der Mark E, Matile H, D’Arcy B, Magg C, Ringler P, Muller SA, Scherer S, Dernick G, Thoma R, Hennig M, Niesor EJ, Stahlberg H, Cholesteryl ester transfer between lipoproteins does not require a ternary tunnel complex with CETP, J. Struct. Biol 194 (2) (2016) 191–198 (5). 26876146 10.1016/j.jsb.2016.02.016

[55] Morton RE, Binding of plasma-derived lipid transfer protein to lipoprotein substrates. The role of binding in the lipid transfer process, J. Biol. Chem 260 (1985) 12593–12599. 4044601

[56] Clark RW, Ruggeri RB, Cunningham D, Bamberger MJ, Description of the torcetrapib series of cholesteryl ester transfer protein inhibitors, including mechanism of action, J. Lipid Res. 47 (2006) 537–552. 16326978 10.1194/jlr.M500349-JLR200

[57] Krishna R, Anderson MS, Bergman AJ, Jin B, Fallon M, Cote J, Rosko K, Chavez-Eng C, Lutz R, Bloomfield DM, Gutierrez M, Doherty J, Bieberdorf F, Chodakewitz J, Gottesdiener KM, Wagner JA, Effect of the cholesteryl ester transfer protein inhibitor, anacetrapib, on lipoproteins in patients with dyslipidaemia and on 24-h ambulatory blood pressure in healthy individuals: two double-blind, randomised placebo-controlled phase I studies, Lancet 370 (2007) 1907–1914. 18068514 10.1016/S0140-6736(07)61813-3

[58] Stein EA, Stroes ES, Steiner G, Buckley BM, Capponi AM, Burgess T, Niesor EJ, Kallend D, Kastelein JJ, Safety and tolerability of dalcetrapib, Am. J. Cardiol 104 (2009) 82–91. 19576325 10.1016/j.amjcard.2009.02.061

[59] Funder JW, The off-target effects of torcetrapib, Endocrinology 150 (2009) 2024–2026. 19383878 10.1210/en.2009-0136

[60] Rohrl C, Stangl H, HDL endocytosis and resecretion, Biochim. Biophys. Acta 1831 (2013) 1626–1633. 23939397 10.1016/j.bbalip.2013.07.014 PMC3795453

[61] Han S, Flattery AM, McLaren D, Raubertas R, Lee SH, Mendoza V, Rosa R, Geoghagen N, Castro-Perez JM, Roddy TP, Forrest G, Johns D, Hubbard BK, Li J, Comparison of lipoprotein separation and lipid analysis methodologies for human and cynomolgus monkey plasma samples, J. Cardiovasc. Transl. Res 5 (2012) 75–83. 22194019 10.1007/s12265-011-9340-9

[62] Caulfield MP, Li S, Lee G, Blanche PJ, Salameh WA, Benner WH, Reitz RE, Krauss RM, Direct determination of lipoprotein particle sizes and concentrations by ion mobility analysis, Clin. Chem 54 (2008) 1307–1316. 18515257 10.1373/clinchem.2007.100586

[63] Grigorieff N, FREALIGN: high-resolution refinement of single particle structures, J. Struct. Biol 157 (2007) 117–125. 16828314 10.1016/j.jsb.2006.05.004

[64] Frank J, Radermacher M, Penczek P, Zhu J, Li YH, Ladjadj M, Leith A, SPIDER and WEB: processing and visualization of images in 3D electron microscopy and related fields, J. Struct. Biol 116 (1996) 190–199. 8742743 10.1006/jsbi.1996.0030

[65] Tang G, Peng L, Baldwin PR, Mann DS, Jiang W, Rees I, Ludtke SJ, EMAN2: An extensible image processing suite for electron microscopy, J. Struct. Biol 157 (2007) 38–46. 16859925 10.1016/j.jsb.2006.05.009

[66] Ludtke SJ, Baldwin PR, Chiu W, EMAN: semiautomated software for high-resolution single-particle reconstructions, J. Struct. Biol 128 (1999) 82–97. 10600563 10.1006/jsbi.1999.4174

[67] Galkin VE, Orlova A, Cherepanova O, Lebart MC, Egelman EH, High-resolution cryo-EM structure of the F-actin-fimbrin/plastin ABD2 complex, Proc. Natl. Acad. Sci. U. S. A 105 (2008) 1494–1498. 18234857 10.1073/pnas.0708667105 PMC2234172

[68] Bottcher B, Wynne SA, Crowther RA, Determination of the fold of the core protein of hepatitis B virus by electron cryomicroscopy, Nature 386 (1997) 88–91. 9052786 10.1038/386088a0

