# Supplementary material for: Assessing the mechanisms of cholesteryl ester transfer protein inhibitors
Source: Biochim Biophys Acta Mol Cell Biol Lipids. Author manuscript; Available in PMC 2018 Nov 16. (PMC6239860; doi:10.1016/j.bbalip.2017.09.004)
Supplement: supporting [file NIHMS993341-supplement-supporting.pdf]

SUPPLEMENTAL FIGURE LEGENDS

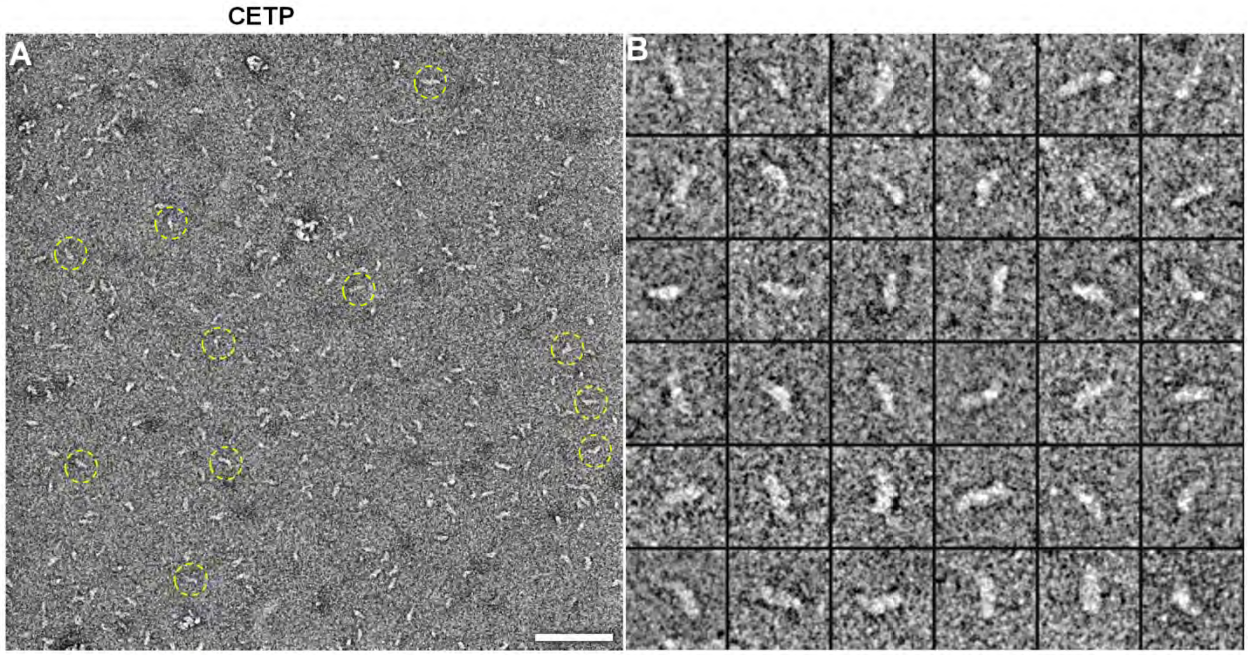

Supplemental Figure 1**. Structure of CETP, as shown by optimized negative-staining (OpNS) EM.** A) Survey view of OpNS EM image of CETPs. The CETP particles are marked by yellow circles. B) Thirty-six representative images of CETP particles. Scale bar: 80 nm, particle window size: 30 nm.

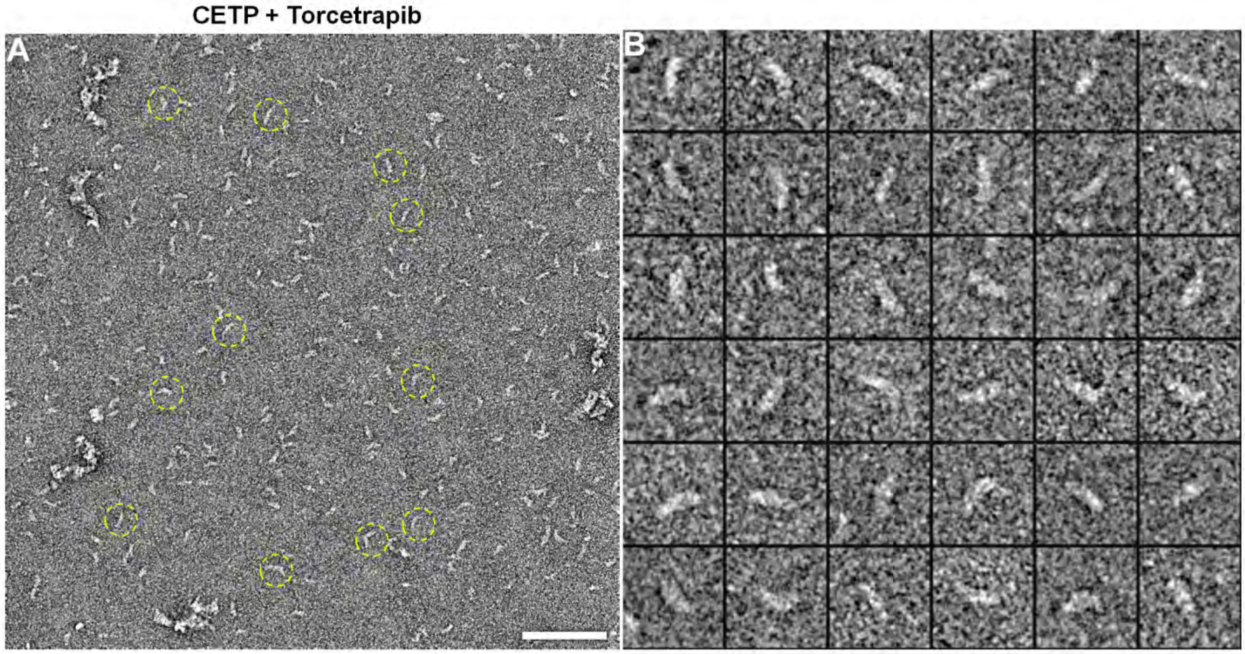

Supplemental Figure 2**. Structure of CETP incubated with Torcetrapib, as shown by OpNS EM.** A) Survey view of OpNS EM image of CETP incubated with Torcetrapib at 37°C for up to 1 minute. The CETP particles are marked by yellow circles. B) Thirty-six representative images of CETP particles. Scale bar: 80

nm, particle window size: 30 nm.

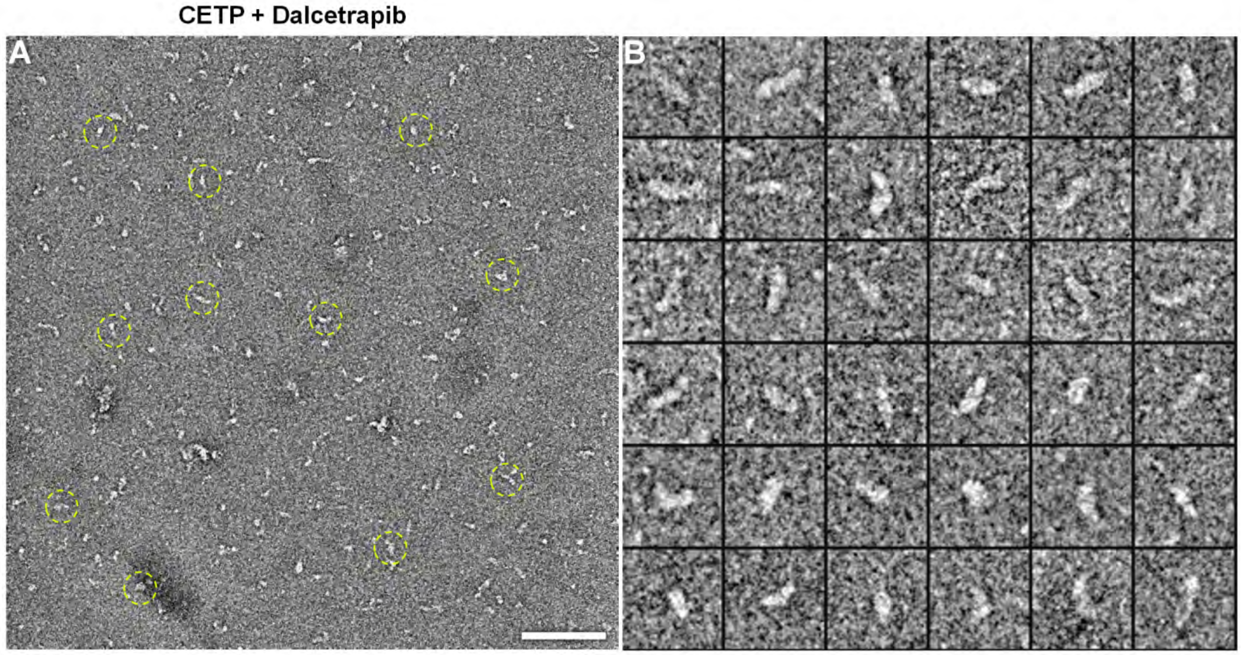

Supplemental Figure 3**. Structure of CETP incubated with Dalcetrapib, as shown by OpNS EM.** A) Survey view of OpNS EM image of CETP incubated with Dalcetrapib at 37°C for up to 1 minute. The CETP particles are marked by yellow circles. B) Thirty-six representative images of CETP particles. Scale bar: 80 nm, particle window size: 30 nm.

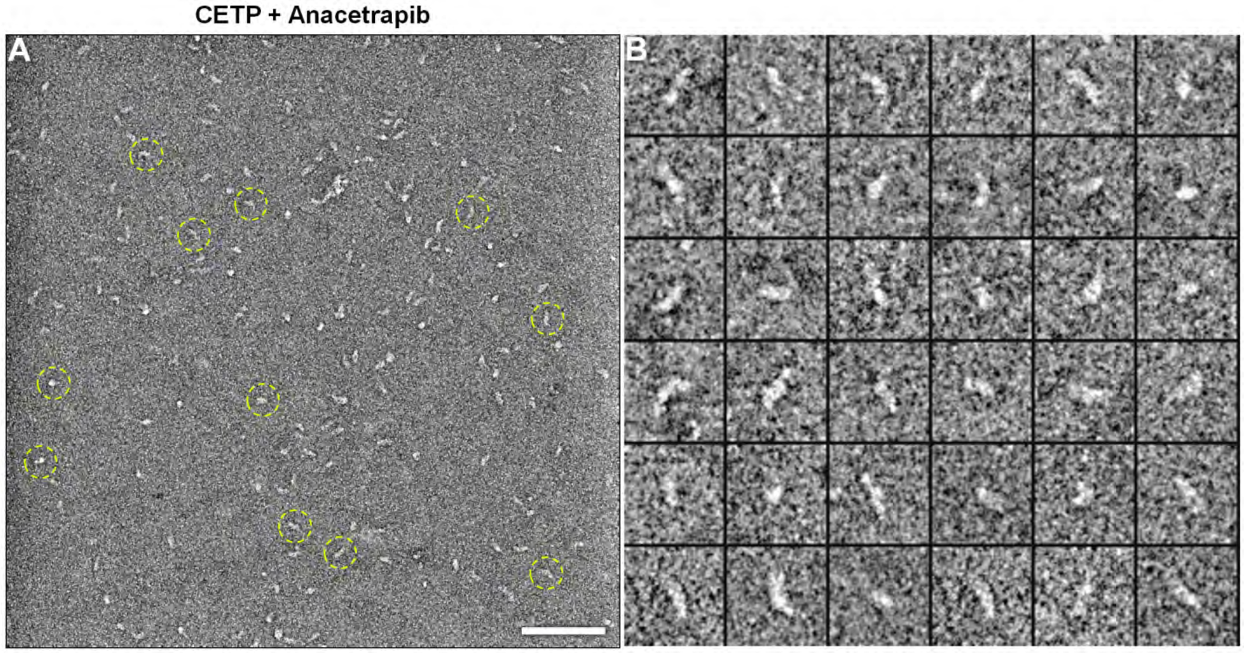

Supplemental Figure 4**. Structure of CETP incubated with Anacetrapib, as shown by OpNS EM.** A) Survey view of OpNS EM image of CETP incubated with Anacetrapib at 37°C for up to 1 minute. The CETP particles are marked by yellow circles. B) Thirty-six representative images of CETP particles. Scale bar: 80 nm, particle window size: 30 nm.

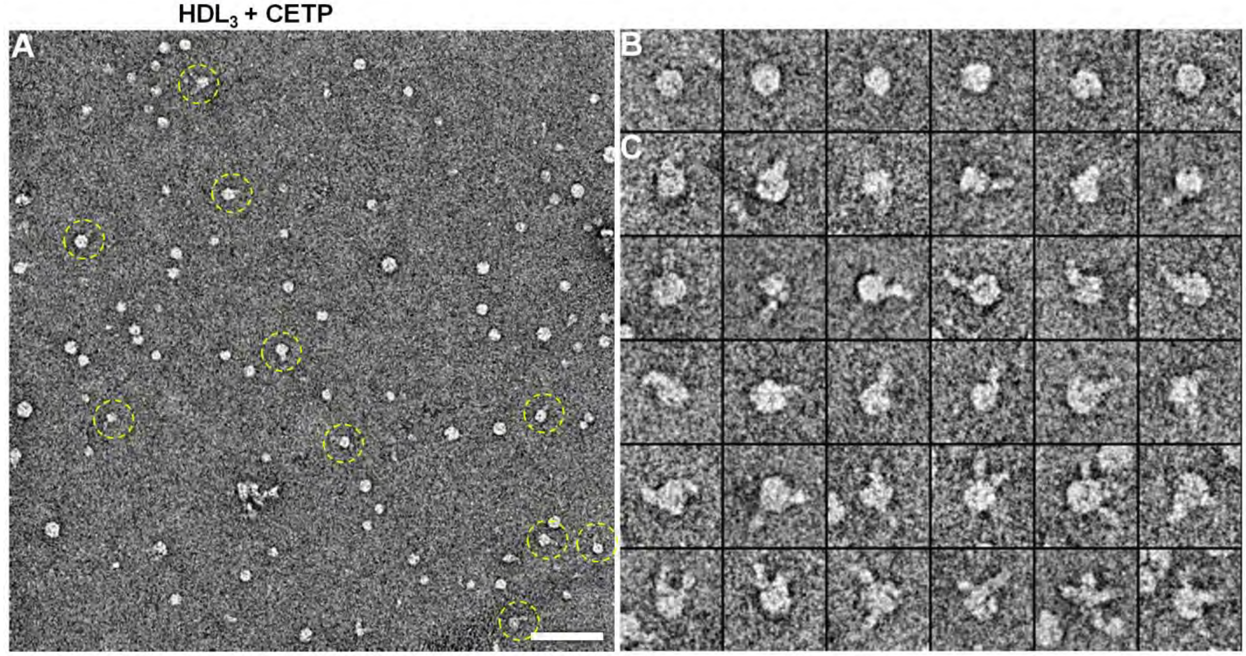

Supplemental Figure 5**. Structure of CETP bound to human plasma HDL_3, as shown by OpNS EM.** A**)** Survey OpNS EM image of human plasma HDL_3 incubated with CETP at 37°C. The complexes of HDL_3 bound to CETP are marked in yellow dashed circles. B) Six representative images of HDL_3 alone, and C) 30 representative images of the complexes of HDL_3 bound to one or more CETPs. Particle window size: 30 nm. Scale bar: 70 nm.

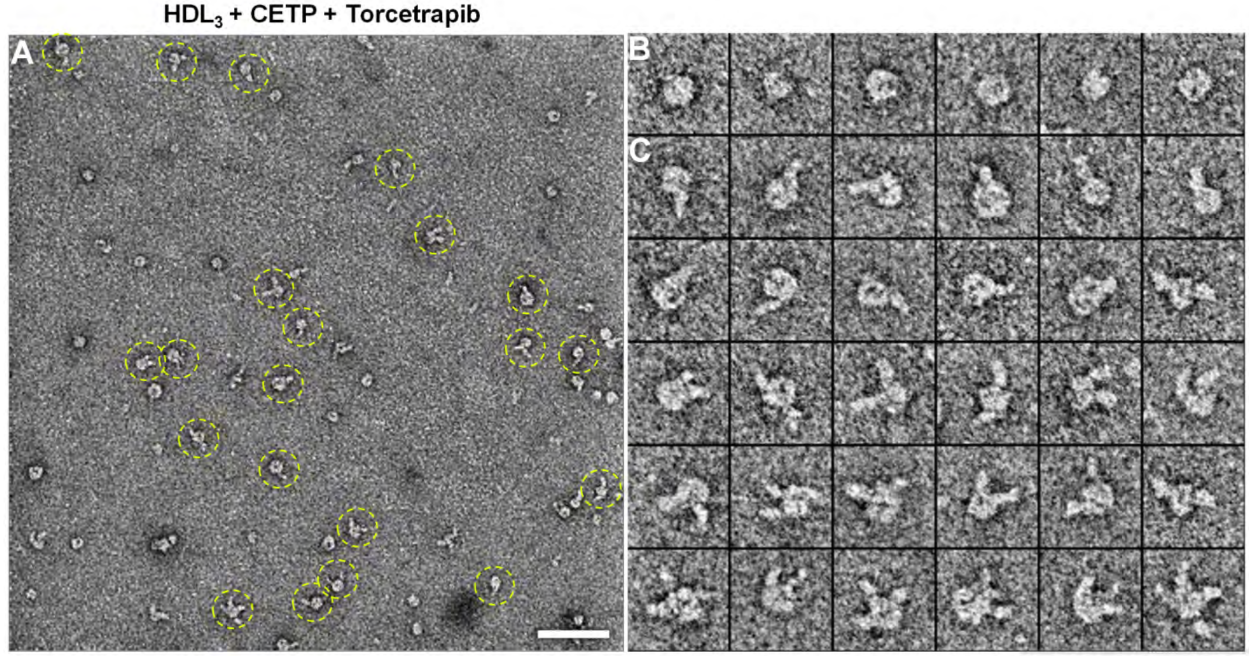

Supplemental Figure 6**. Effects of Torcetrapib on the structure of CETP bound to HDL, as shown by OpNS EM.** A**)** Survey OpNS EM image of human plasma HDL_3 incubated with CETP and Torcetrapib at 37°C. The complexes of HDL_3 bound to CETP are marked in yellow dashed circles. B) Six representative

particle images of HDL_3 alone, and C) 30 representative images of the complexes of HDL_3 bound to one or more CETPs. Particle window size: 30 nm. Scale bar: 70 nm.

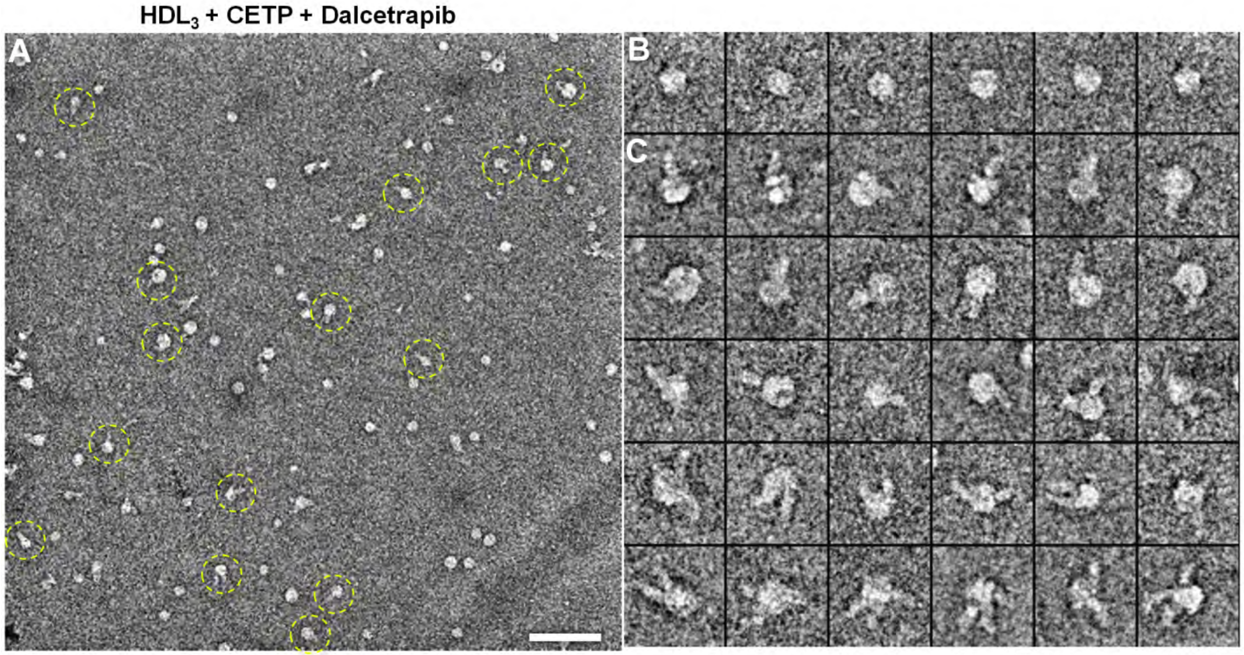

Supplemental Figure 7. **Effects of Dalcetrapib on the structure of CETP bound to HDL, as shown by OpNS EM.** A**)** Survey OpNS EM image of human plasma HDL_3 incubated with CETP and Dalcetrapib at 37°C. The complexes of HDL_3 bound to CETP are marked in yellow dashed circles. B) Six representative particle images of HDL_3 alone, and C) 30 representative images of the complexes of HDL_3 bound to one or more CETPs. Particle window size: 30 nm. Scale bar: 70 nm.

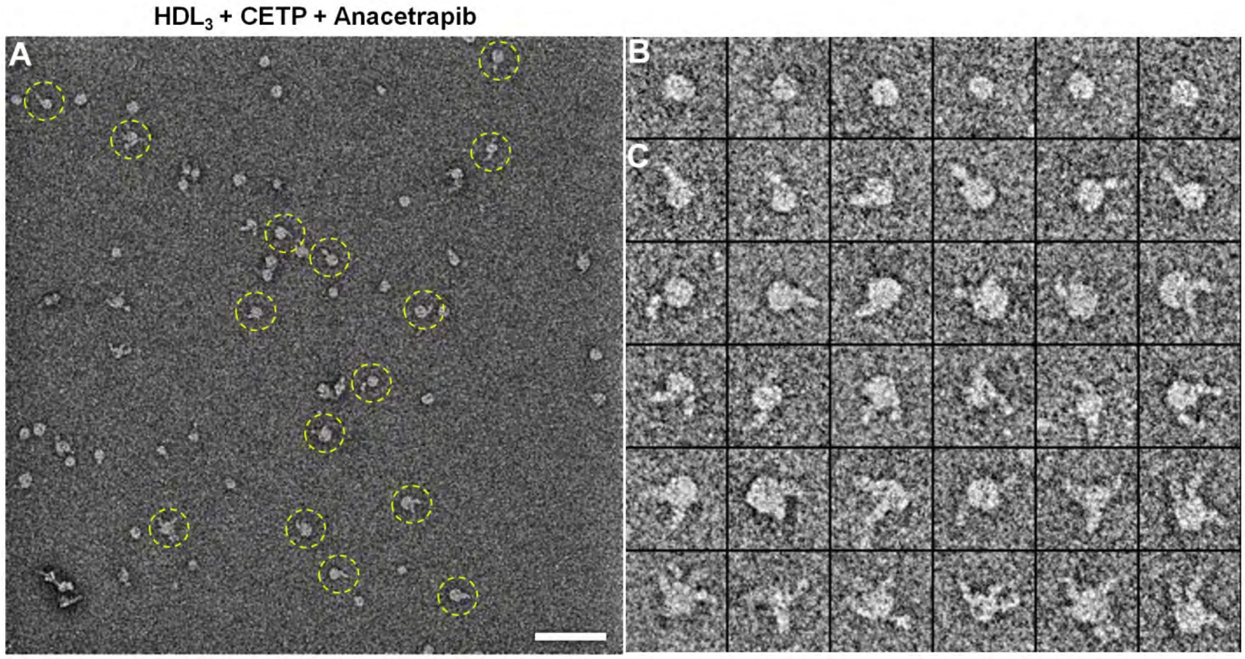

Supplemental Figure 8. **Effects of Anacetrapib on the structure of CETP bound to HDL, as shown by OpNS EM.** A**)** Survey OpNS EM image of human plasma HDL_3 incubated with CETP and Anacetrapib at

37°C. The complexes of HDL_3 bound to CETP are marked in yellow dashed circles. B) Six representative particle images of HDL_3 alone, and C) 30 representative images of the complexes of HDL_3 bound to one or more CETPs. Particle window size: 30 nm. Scale bar: 70 nm.

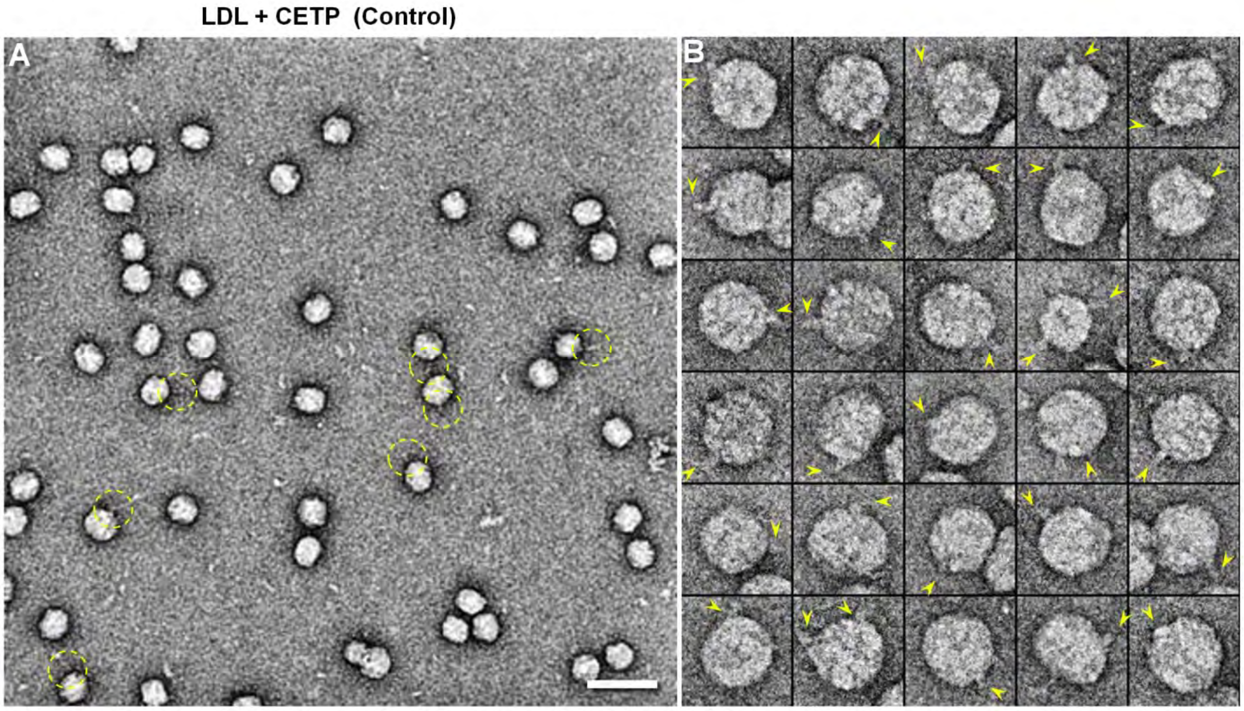

Supplemental Figure 9**. Structure of CETP bound to human plasma LDL, as shown by OpNS EM.** A**)** Survey OpNS EM image of human plasma LDL incubated with CETP at 37°C. The complexes of LDL bound to CETP are marked in yellow dashed circles. B) Thirty representative images of the complexes of LDL bound to one or more CETPs (CETPs are indicated by yellow arrowheads). Particle window size: 45 nm. Scale bar: 63 nm.

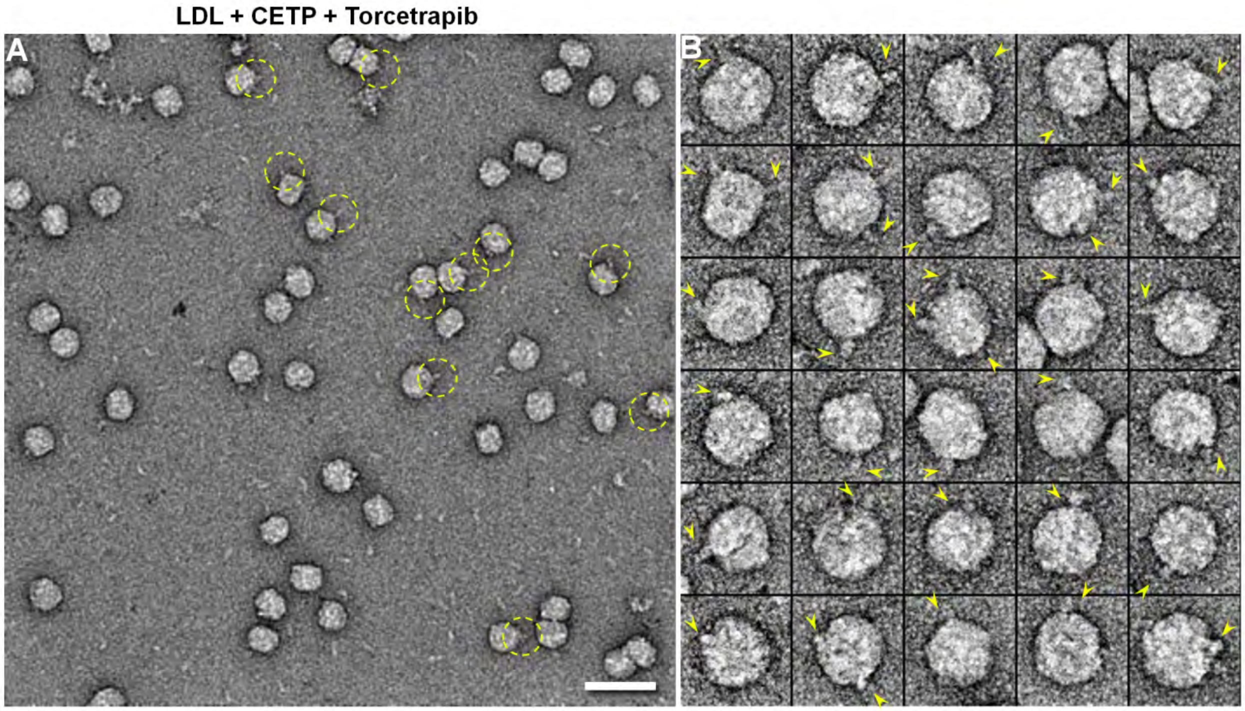

Supplemental Figure 10**. Effects of Torcetrapib on the structure of CETP bound to human plasma LDL, as shown by OpNS EM.** A**)** Survey OpNS EM image of human plasma LDL incubated with CETP and Torcetrapib at 37°C. The complexes of LDL bound to CETP are marked in yellow dashed circles. B) Thirty representative images of the complexes of LDL bound to one or more CETPs (CETPs are indicated by yellow arrows). Particle window size: 45 nm. Scale bar: 63 nm.

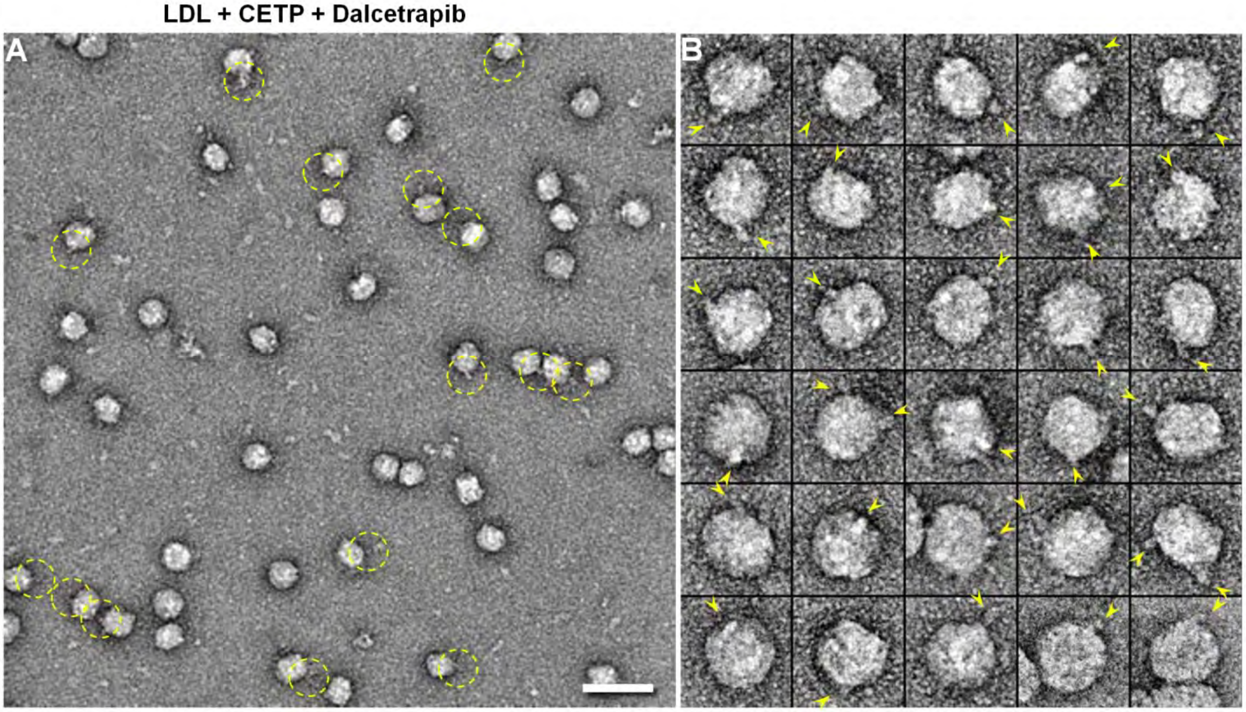

Supplemental Figure 11**. Effects of Dalcetrapib on the structure of CETP bound to human plasma LDL, as shown by OpNS EM.** A**)** Survey OpNS EM image of human plasma LDL incubated with CETP and

Dalcetrapib at 37°C. The complexes of LDL bound to CETP are marked in yellow dashed circles. B) Thirty representative images of the complexes of LDL bound to one or more CETPs (CETPs are indicated by yellow arrows). Particle window size: 45 nm. Scale bar: 63 nm.

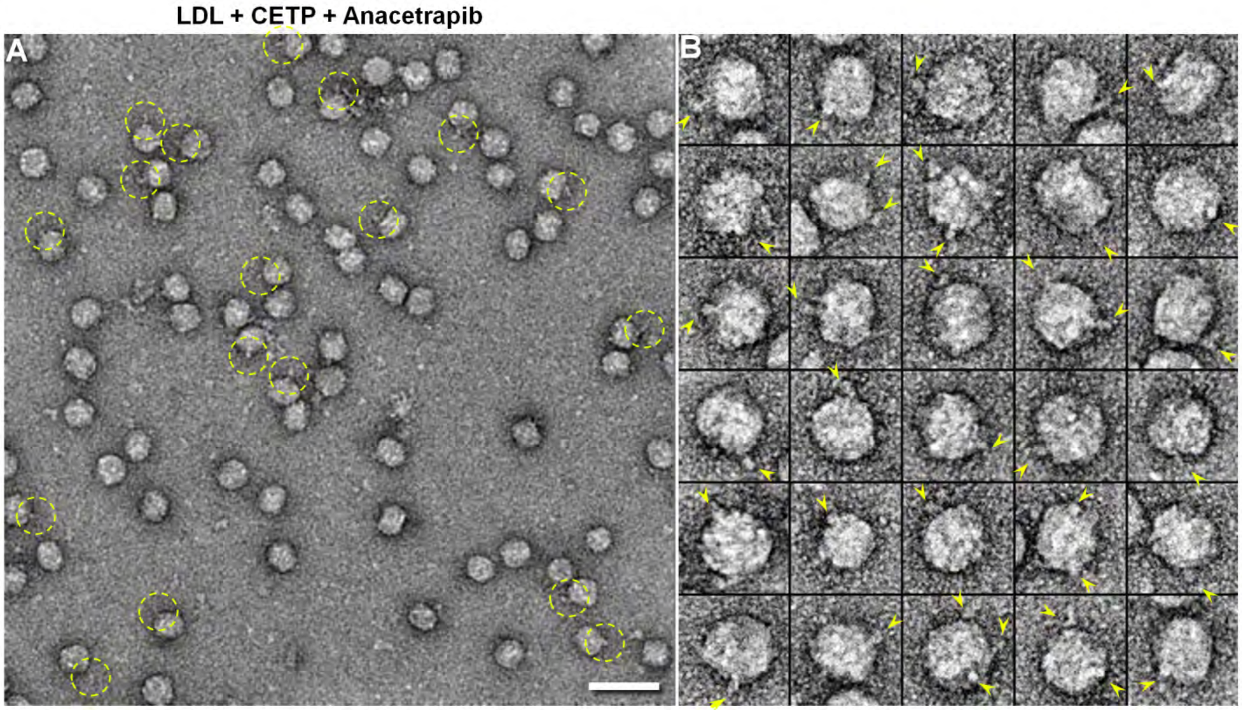

Supplemental Figure 12**. Effects of Anacetrapib on the structure of CETP bound to human plasma LDL, as shown by OpNS EM.** A**)** Survey OpNS EM image of human plasma LDL incubated with CETP and Anacetrapib at 37°C. The complexes of LDL bound to CETP are marked in yellow dashed circles. B) Thirty representative images of the complexes of LDL bound to one or more CETPs (CETPs are indicated by yellow arrows). Particle window size: 45 nm. Scale bar: 63 nm.

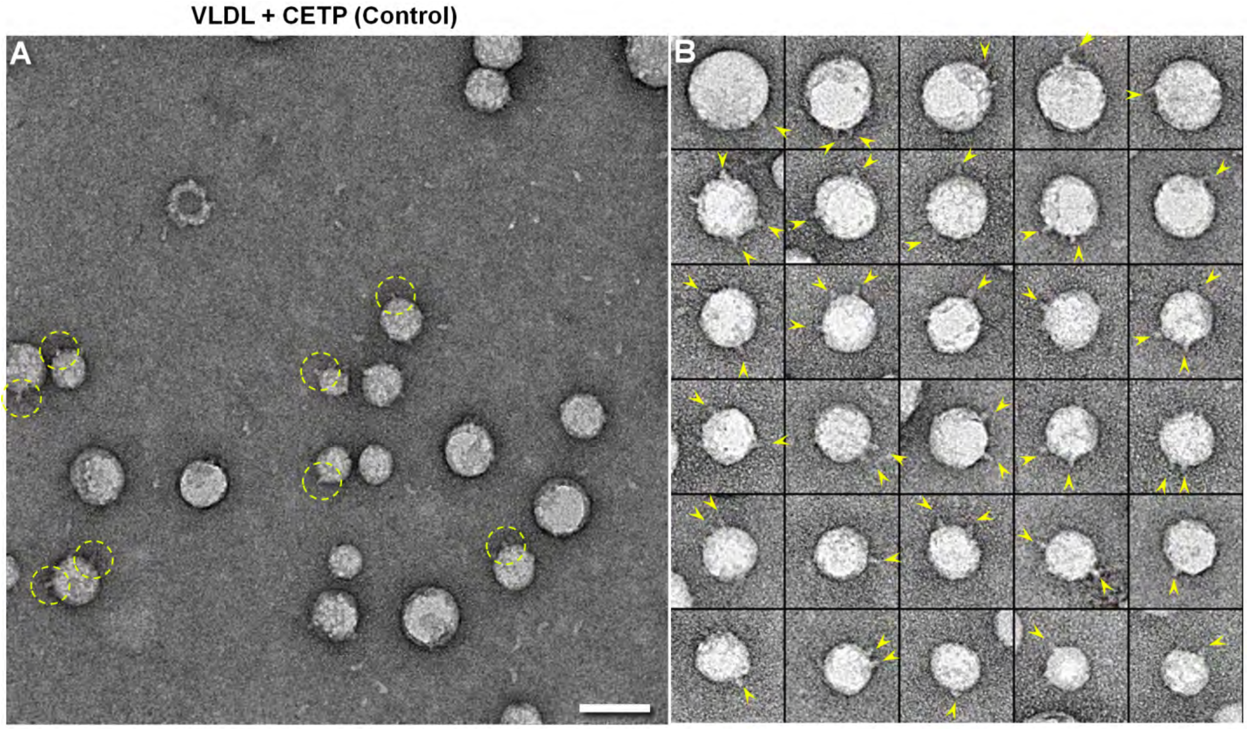

Supplemental Figure 13**. Structure of CETP bound to human plasma VLDL, as shown by OpNS EM.** A**)** Survey OpNS EM image of human plasma VLDL incubated with CETP at 37°C. The complexes of VLDL bound to CETP are marked in yellow dashed circles. B) Thirty representative images of the complexes of VLDL bound to one or more CETPs (CETPs are indicated by yellow arrows). Particle window size: 60 nm. Scale bar: 63 nm.

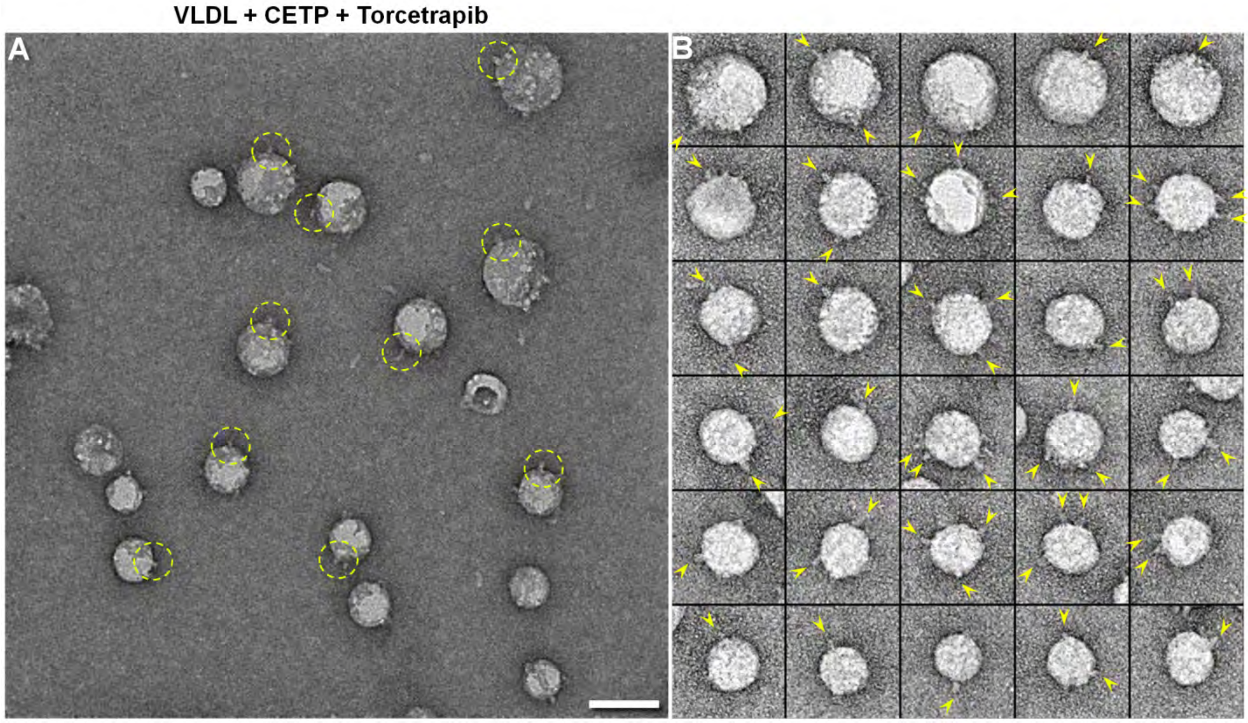

Supplemental Figure 14**. Effects of Torcetrapib on the structure of CETP bound to human plasma VLDL,**

as shown by OpNS EM. A**)** Survey OpNS EM image of human plasma VLDL incubated with CETP and Torcetrapib at 37°C. The complexes of VLDL bound to CETP are marked in yellow dashed circles. B) Thirty representative images of the complexes of VLDL bound to one or more CETPs (CETPs are indicated by yellow arrows). Particle window size: 60 nm. Scale bar: 63 nm.

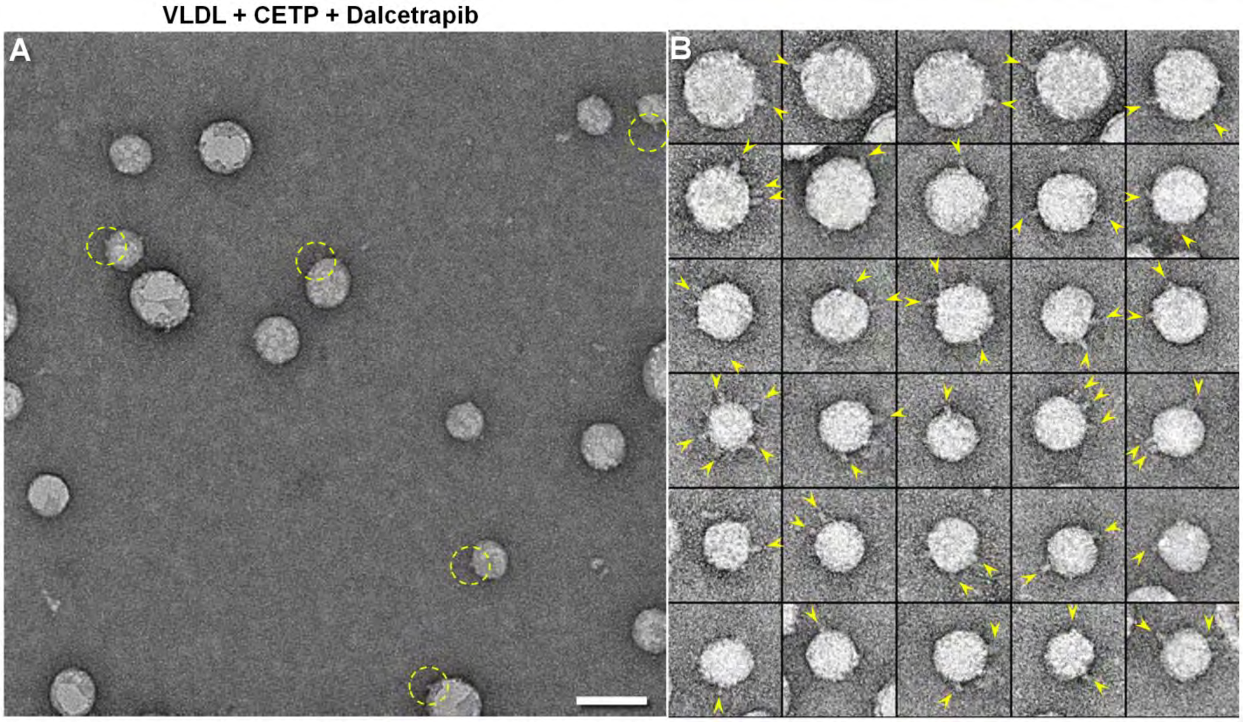

Supplemental Figure 15**. Effects of Dalcetrapib on the structure of CETP bound to human plasma VLDL, as shown by OpNS EM.** A**)** Survey OpNS EM image of human plasma VLDL incubated with CETP and Dalcetrapib at 37°C. The complexes of VLDL bound to CETP are marked in yellow dashed circles. B) Thirty representative images of the complexes of VLDL bound to one or more CETPs (CETPs are indicated by yellow arrows). Particle window size: 60 nm. Scale bar: 63 nm.

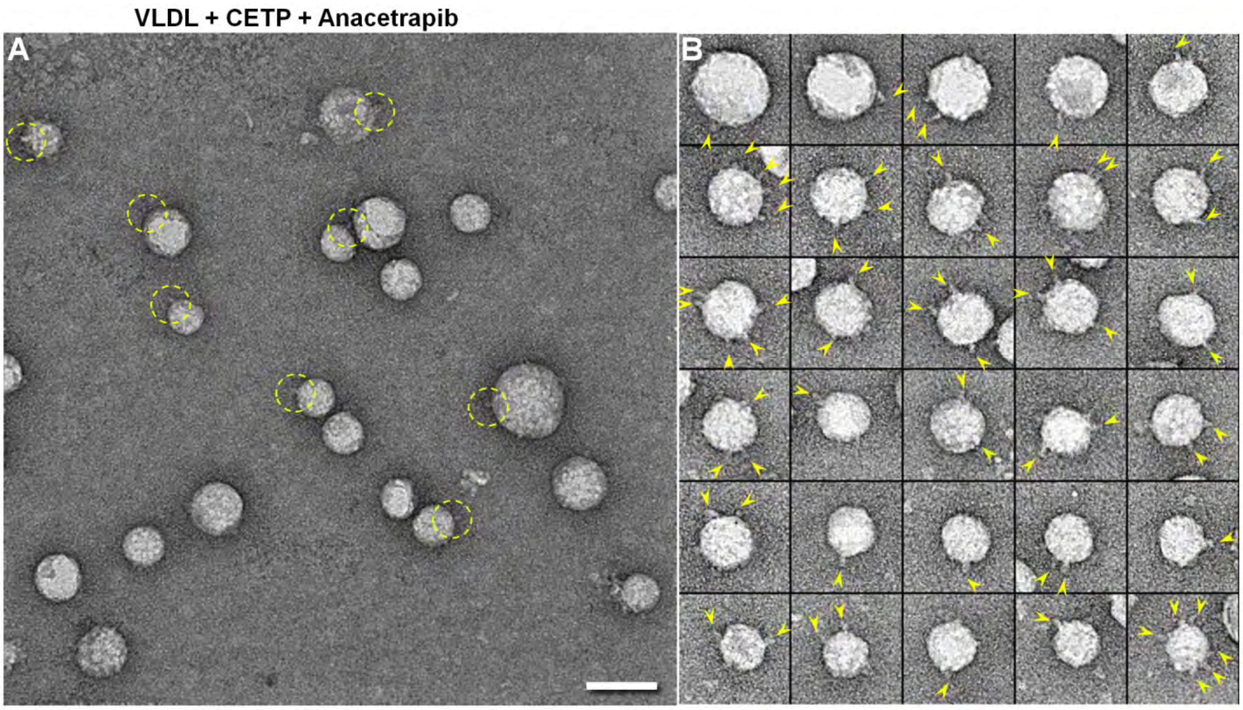

Supplemental Figure 16**. The effects of Anacetrapib on the structure of CETP bound to human plasma VLDL, as shown by OpNS EM.** A**)** Survey OpNS EM image of human plasma VLDL incubated with CETP and Anacetrapib at 37°C. The complexes of VLDL bound to CETP are marked in yellow dashed circles. B) Thirty representative images of the complexes of VLDL bound to one or more CETPs (CETPs are indicated by yellow arrows). Particle window size: 60 nm. Scale bar: 63 nm.

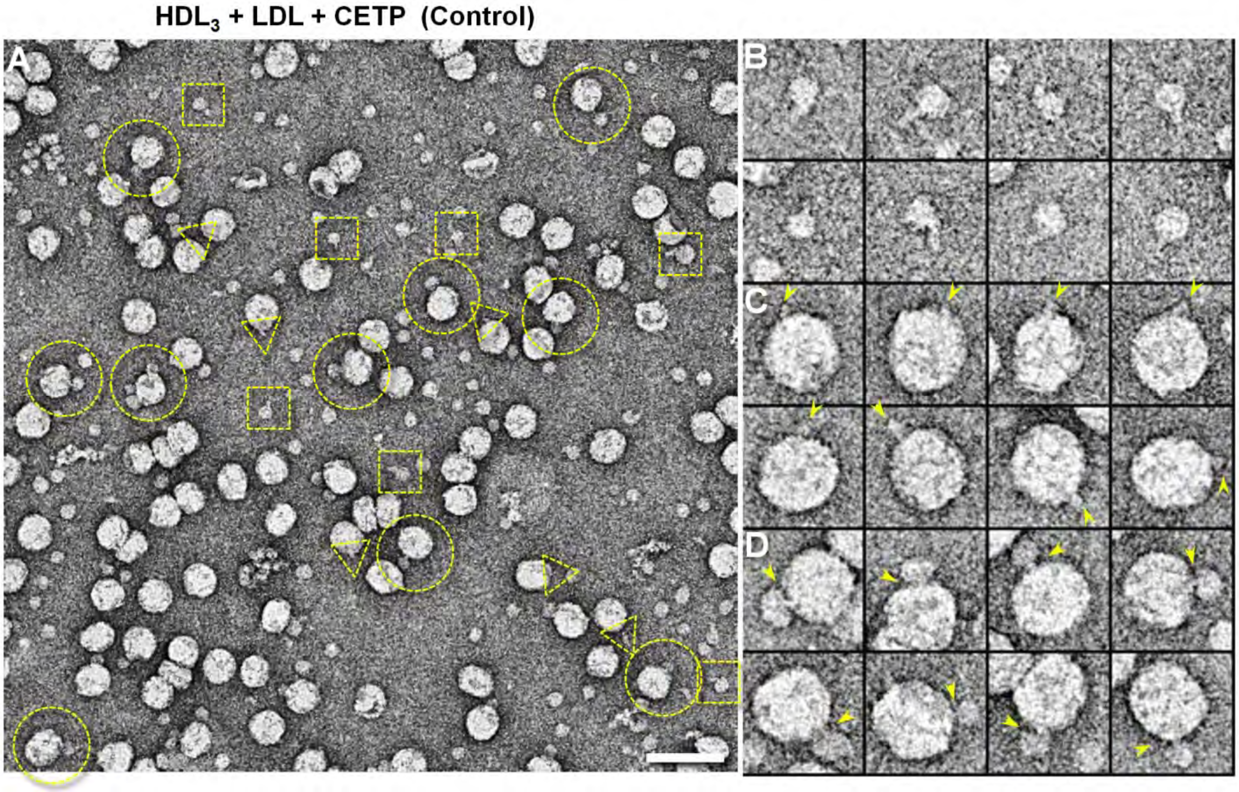

Supplemental Figure 17**. Structure of CETP bound to human plasma HDL_3 and LDL, as shown by OpNS EM.** A**)** Survey OpNS EM image of CETP incubated with human plasma HDL_3 and LDL simultaneously at 37°C. The ternary complexes of CETP bridging HDL_3 and LDL are marked in yellow circles, CETP bound to HDL_3 is marked in yellow square boxes, and CETP bound to LDL is marked in yellow triangles. B) Eight representative images of binary complexes of CETP bound to HDL_3. C) Eight representative images of binary complexes of CETP bound to LDL (CETPs are indicated by yellow arrows). D) Eight representative images of ternary complexes of CETP bound to HDL_3 and LDL simultaneously. Particle window size: 48 nm. Scale bar: 57 nm.

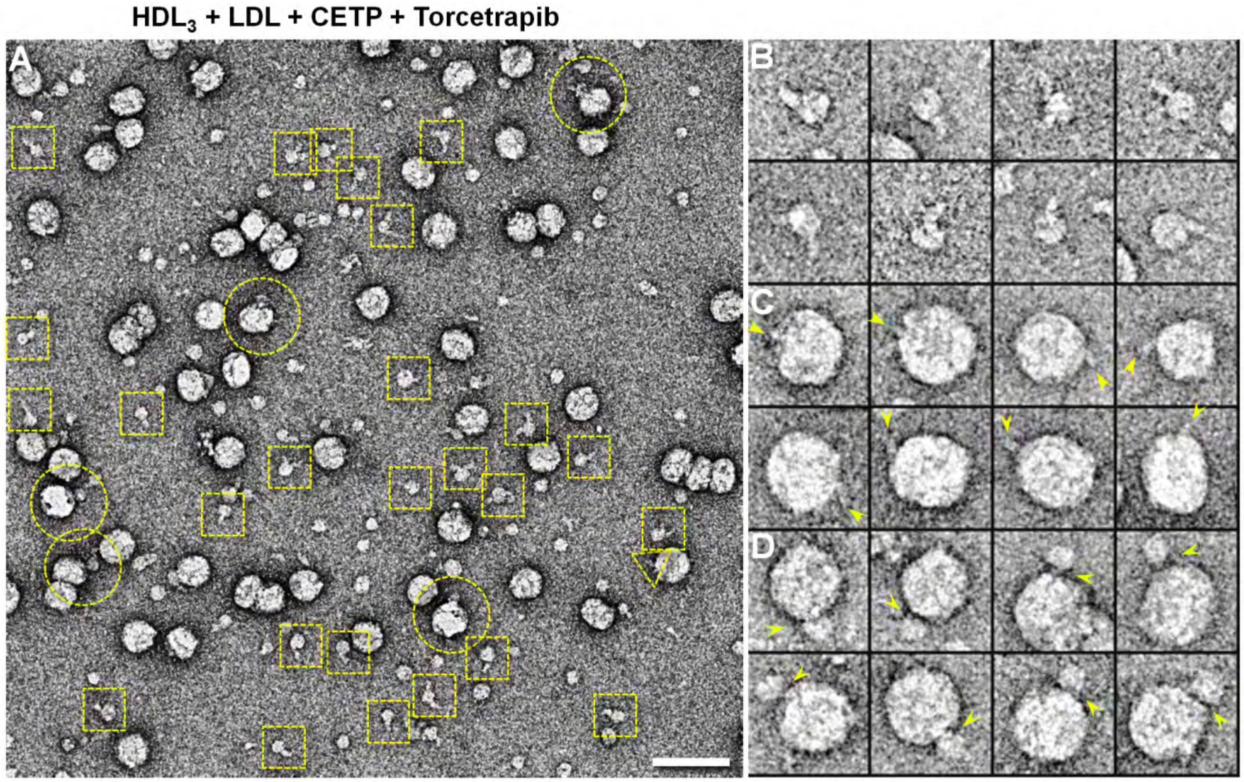

Supplemental Figure 18**. Effects of Torcetrapib on the structure of CETP bound to human plasma HDL_3 and LDL, as shown by OpNS EM.** A**)** Survey OpNS EM image of CETP incubated with Torcetrapib, human plasma HDL_3 and LDL simultaneously at 37°C. The ternary complexes of CETP bridging HDL_3 and LDL are marked in yellow circles, CETP bound to HDL_3 is marked in yellow square boxes, and CETP bound to LDL is marked in yellow triangles. B) Eight representative images of binary complexes of CETP bound to HDL_3. C) Eight representative images of binary complexes of CETP bound to LDL (CETPs are indicated by yellow arrows). D) Eight representative images of ternary complexes of CETP bound to HDL_3 and LDL simultaneously. Particle window size: 48 nm. Scale bar: 57 nm.

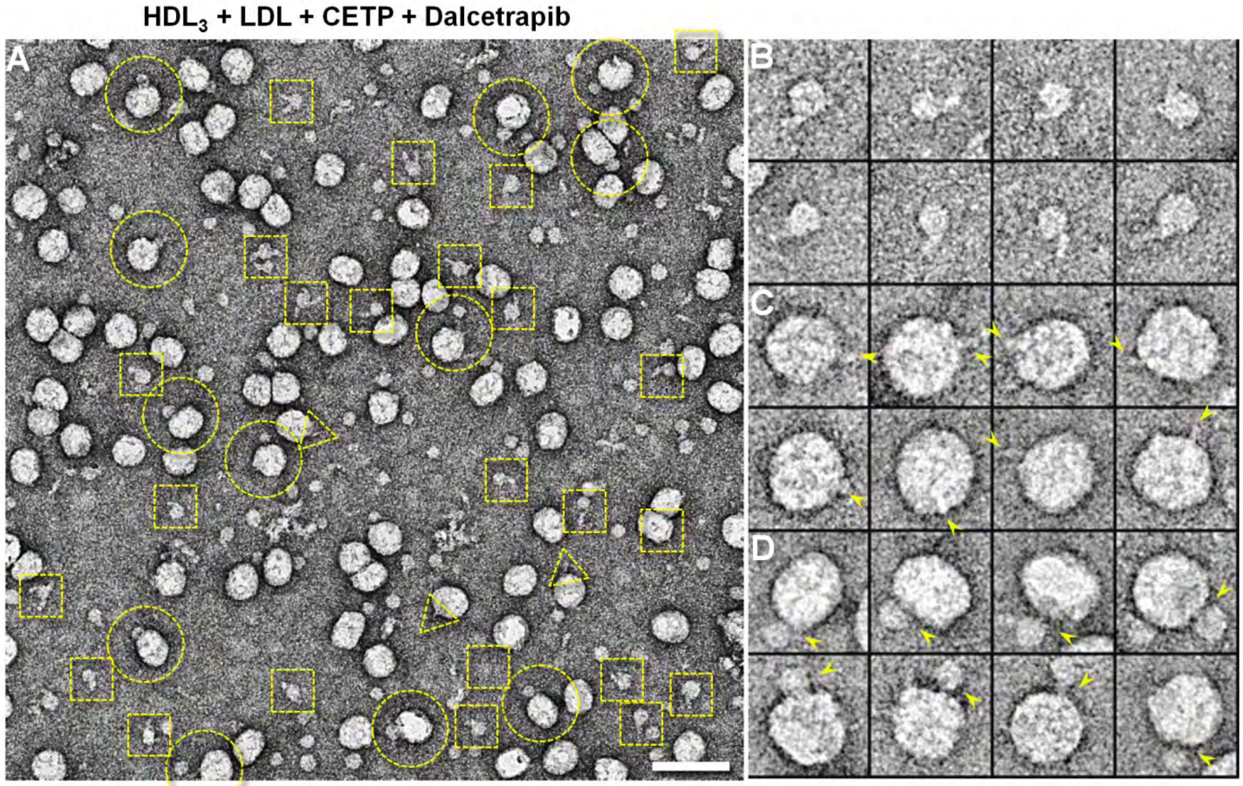

Supplemental Figure 19**. Effects of Dalcetrapib on the structure of CETP bound to human plasma HDL_3 and LDL, as shown by OpNS EM.** A**)** Survey OpNS EM image of CETP incubated with Dalcetrapib, human plasma HDL_3 and LDL simultaneously at 37°C. The ternary complexes of CETP bridging HDL_3 and LDL are marked in yellow circles, CETP bound to HDL_3 is marked in yellow square boxes, and CETP bound to LDL is marked in yellow triangles. B) Eight representative images of binary complexes of CETP bound to HDL_3. C) Eight representative images of binary complexes of CETP bound to LDL (CETPs are indicated by yellow arrows). D) Eight representative images of ternary complexes of CETP bound to HDL_3 and LDL simultaneously. Particle window size: 48 nm. Scale bar: 57 nm.

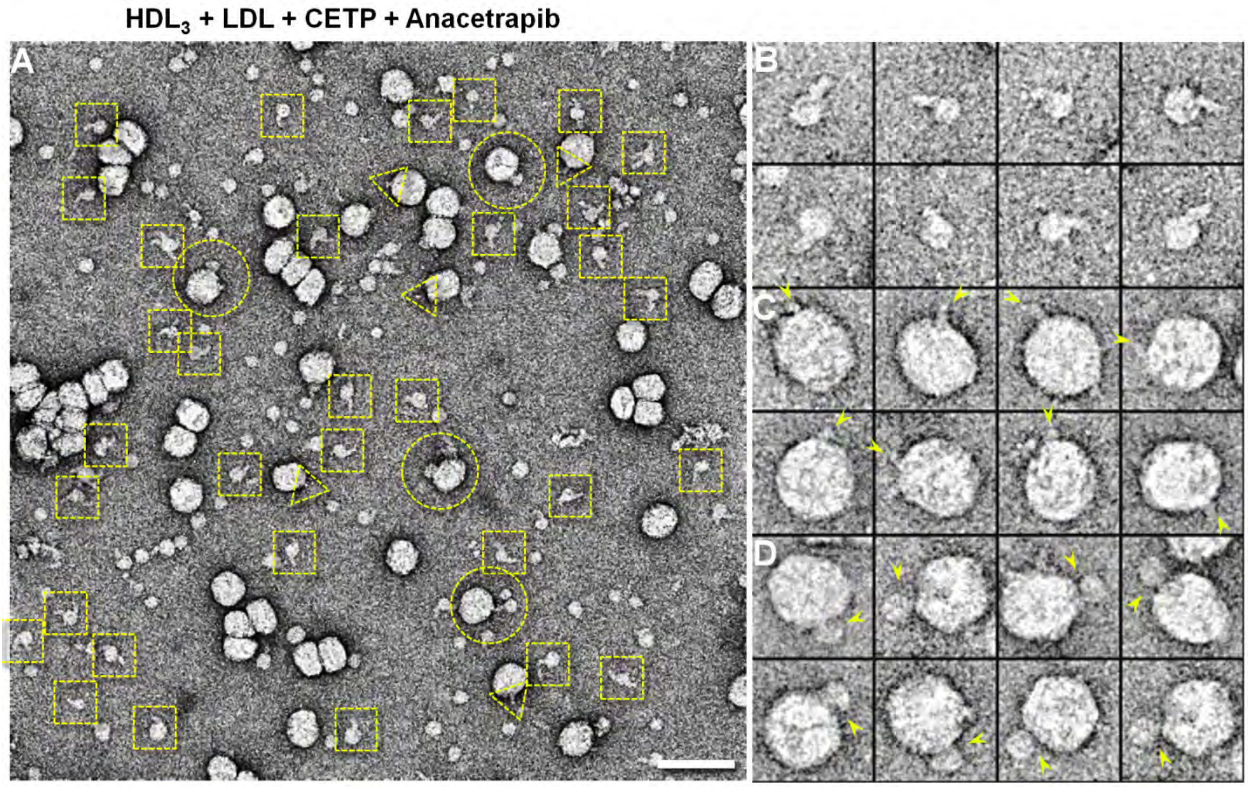

Supplemental Figure 20**. Effects of Anacetrapib on the structure of CETP bound to human plasma HDL_3 and LDL, as shown by OpNS EM.** A**)** Survey OpNS EM image of CETP simultaneously incubated with Anacetrapib, human plasma HDL_3 and LDL at 37°C. The ternary complexes of CETP bridging HDL_3 and LDL are marked in yellow circles, CETP bound to HDL_3 is marked in yellow square boxes, and CETP bound to LDL is marked in yellow triangles. B) Eight representative images of binary complexes of CETP bound to HDL_3. C) Eight representative images of binary complexes of CETP bound to LDL (CETPs are indicated by yellow arrows). D) Eight representative images of ternary complexes of CETP bound to HDL_3 and LDL simultaneously. Particle window size: 48 nm. Scale bar: 57 nm.

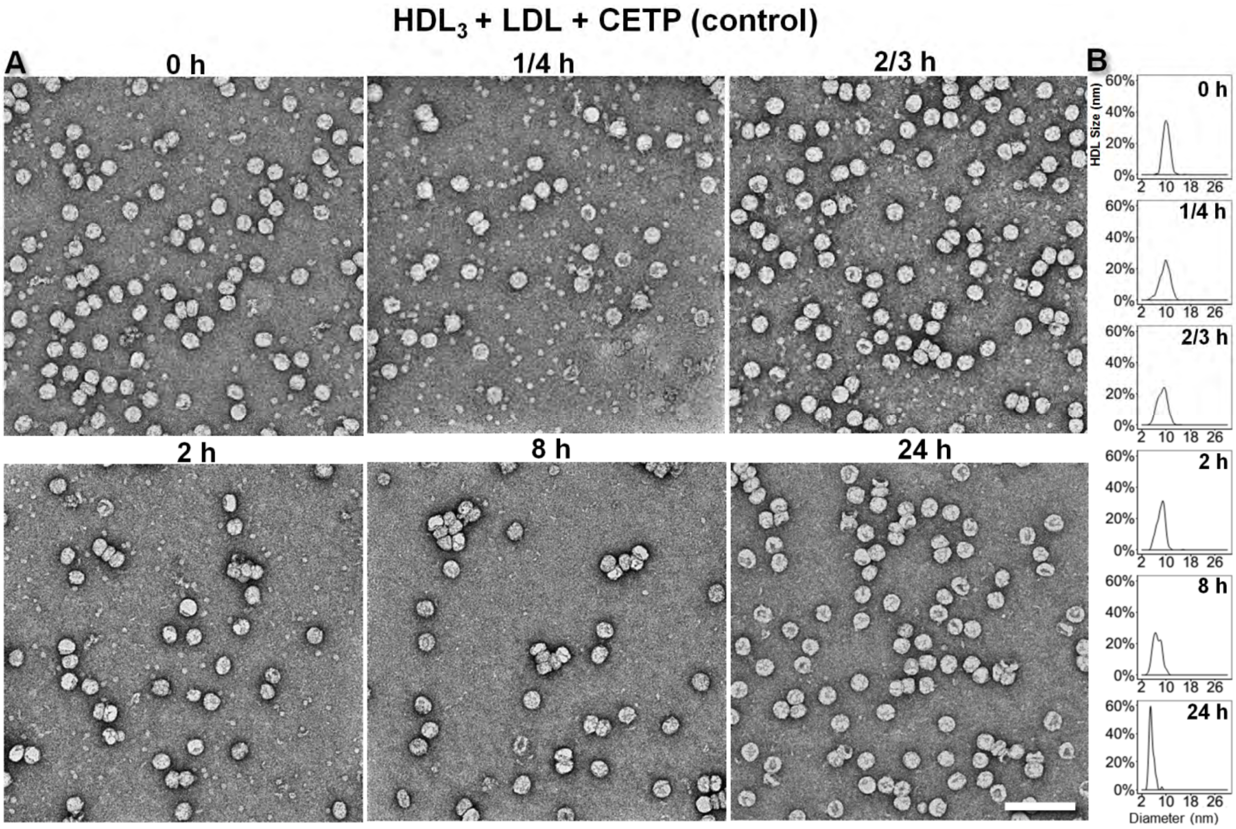

Supplemental Figure 21**. Examination of the CE transfer rate between plasma HDL$_3$ and LDL, as shown by OpNS EM.** A) Samples of CETP incubated with human plasma HDL$_3$ and LDL at 37°C were examined by OpNS EM. Survey views of OpNS EM images of the samples after 6 representative incubation times (0 min, 15 min, 40 min, 2 hours, 8 hours and 24 hours) are presented. B) Histograms of HDL diameters measured from each EM imaged under each incubation time are shown. A total of 300–500 HDL$_3$ particles from each sample were used for the diameter measurement based on the geometric mean of two diameters of each HDL particle, the longest diameter and its perpendicular diameter. Scale bar: 120 nm.

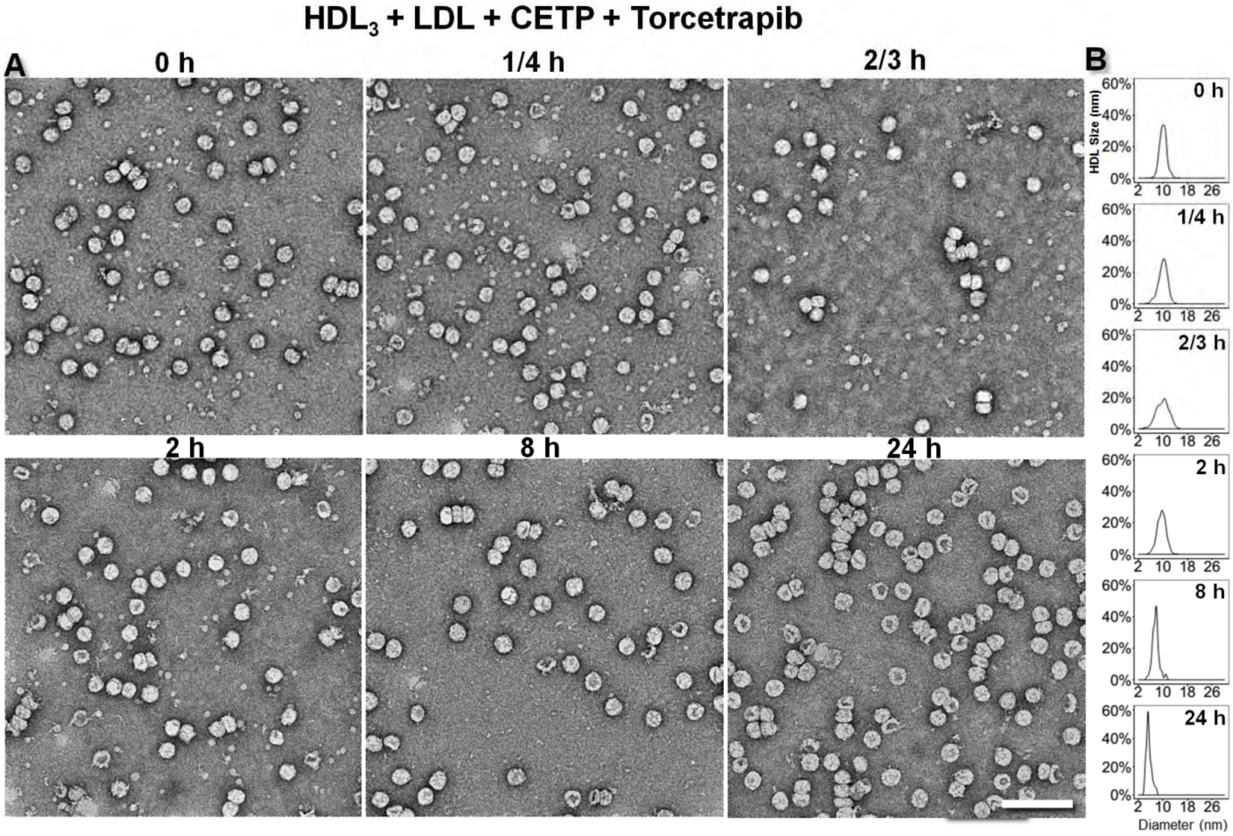

Supplemental Figure 22**. Examination of the effects of Torcetrapib on the CE transfer rate between plasma HDL_3 and LDL, as shown by OpNS EM.** A) Samples of CETP incubated with Torcetrapib, human plasma HDL_3 and LDL at 37°C were examined by OpNS EM. Survey views of OpNS EM images of the samples after 6 representative incubation times (0 min, 15 min, 40 min, 2 hours, 8 hours and 24 hours) are presented. B) Histograms of HDL diameters measured from each EM imaged under each incubation time are shown. A total of 300–500 HDL_3 particles from each sample were used for the diameter measurement based on the geometric mean of two diameters of each HDL particle, the longest diameter and its perpendicular diameter. Scale bar: 120 nm.

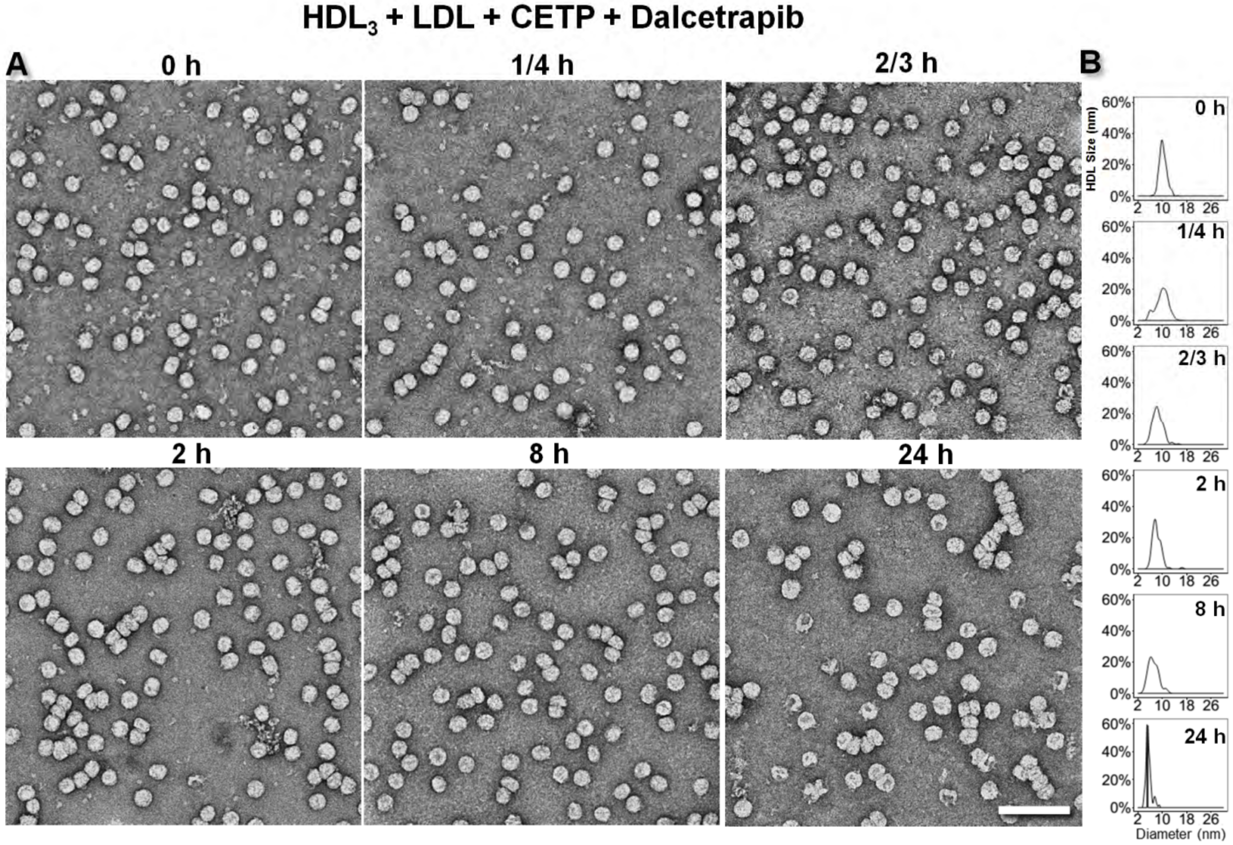

Supplemental Figure 23**. Examination of the effects of Dalcetrapib effects on the CE transfer rate between plasma HDL_3 and LDL, as shown by OpNS EM.** A) Samples of CETP incubated with Dalcetrapib, human plasma HDL_3 and LDL at 37°C were examined by OpNS EM. Survey views of OpNS EM images of the samples after 6 representative incubation times (0 min, 15 min, 40 min, 2 hours, 8 hours and 24 hours) are presented. B) Histograms of HDL diameters measured from each EM imaged under each incubation time are shown. A total of 300–500 HDL_3 particles from each sample were used for the diameter measurement based on the geometric mean of two diameters of each HDL particle, the longest diameter and its perpendicular diameter. Scale bar: 120 nm.

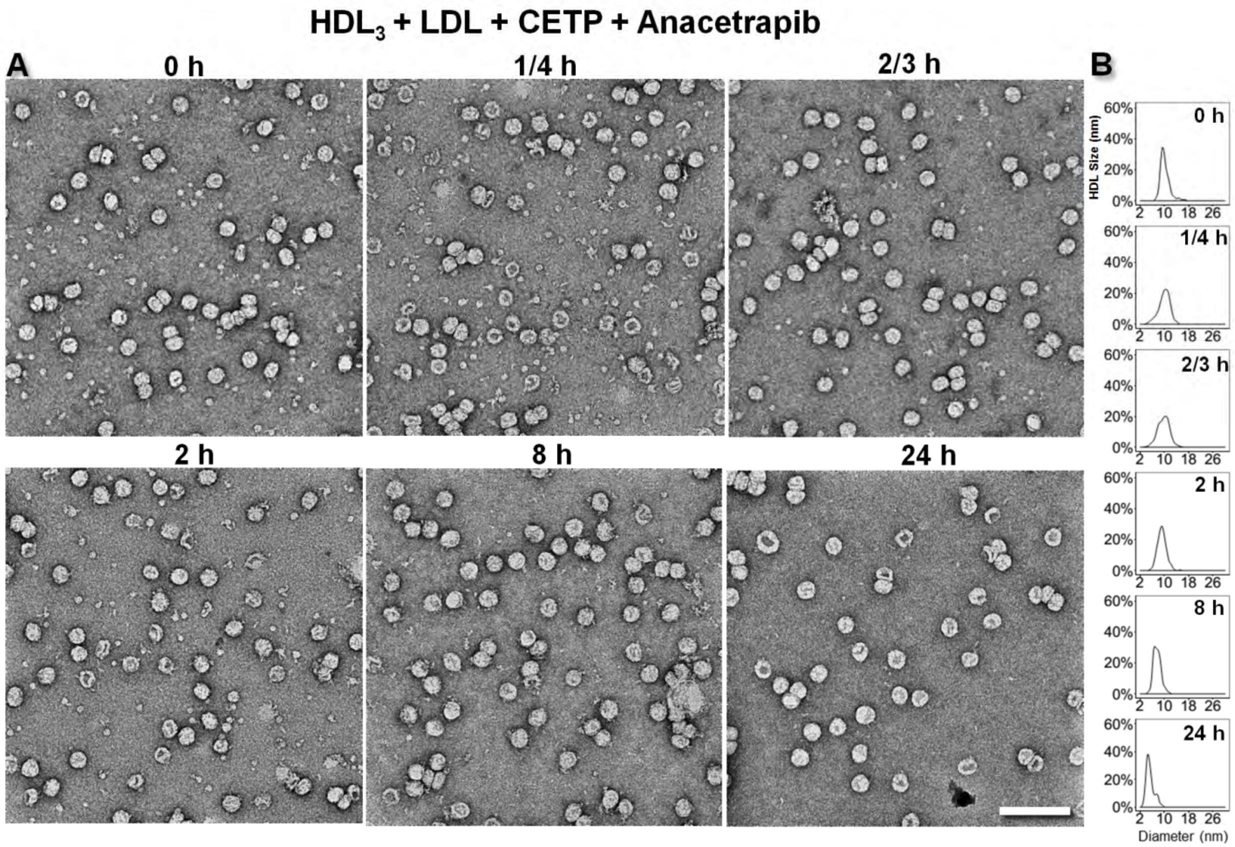

Supplemental Figure 24. **Examination of the effects of Anacetrapib on the CE transfer rate between plasma HDL_3 and LDL, as shown by OpNS EM.** A) Samples of CETP incubated with Anacetrapib, human plasma HDL_3 and LDL at 37°C were examined by OpNS EM. Survey views of OpNS EM images of the samples after 6 representative incubation times (0 min, 15 min, 40 min, 2 hours, 8 hours and 24 hours) are presented. B) Histograms of HDL diameters measured from each EM imaged under each incubation time are shown. A total of 300–500 HDL_3 particles from each sample were used for the diameter measurement based on the geometric mean of two diameters of each HDL particle, the longest diameter and its perpendicular diameter. Scale bar: 120 nm.

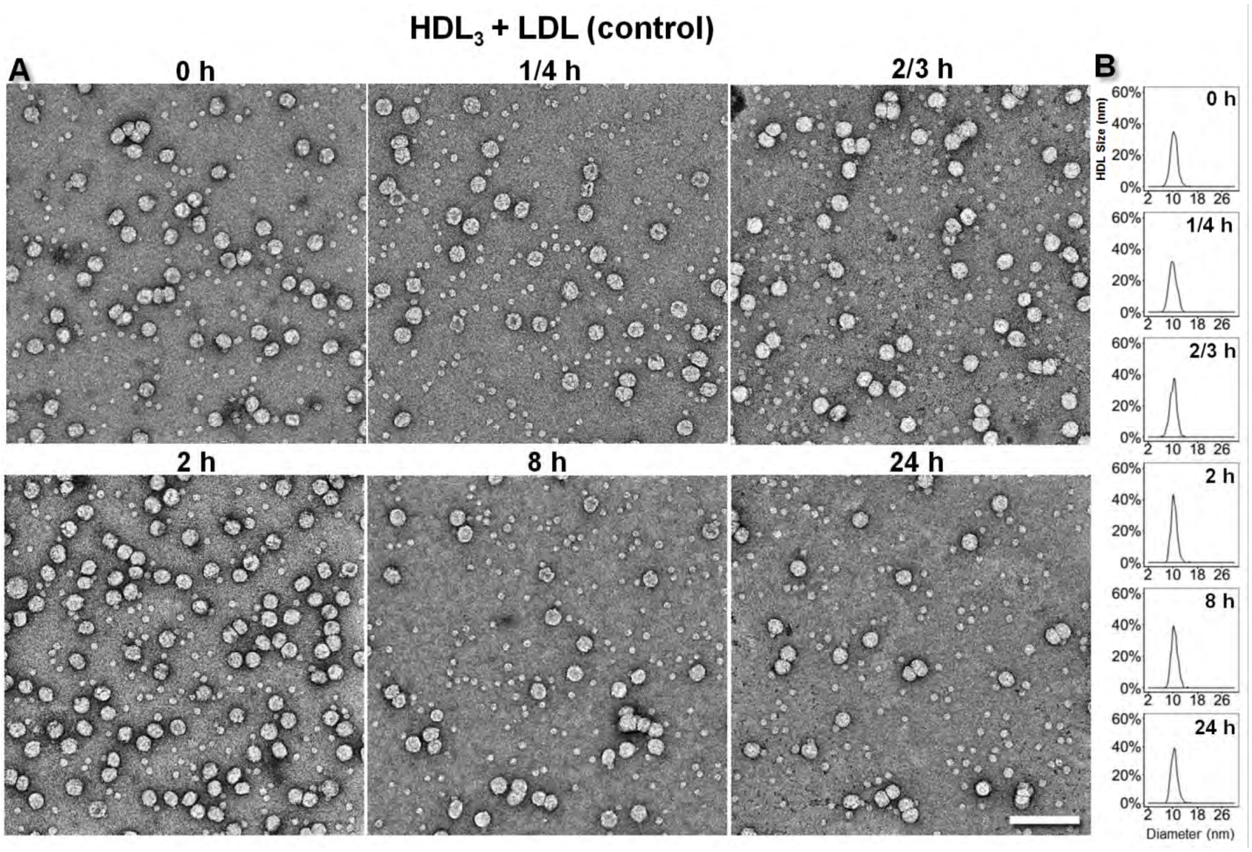

Supplemental Figure 25**. Structure of human plasma HDL_3 incubated with LDL, as shown by OpNS EM.** A**)** As a control, a sample of human plasma HDL_3 was incubated with LDL (without CETP) at 37°C for various lengths of time. Survey views of OpNS EM images of the samples after 6 representative incubation times (0 min, 15 min, 40 min, 2 hours, 8 hours and 24 hours) are presented. B) Histograms of HDL diameters measured from each EM imaged under each incubation time are shown. A total of 300–500 HDL_3 particles from each sample were used for the diameter measurement based on the geometric mean of two diameters of each HDL particle, the longest diameter and its perpendicular diameter. Scale bar: 120 nm.

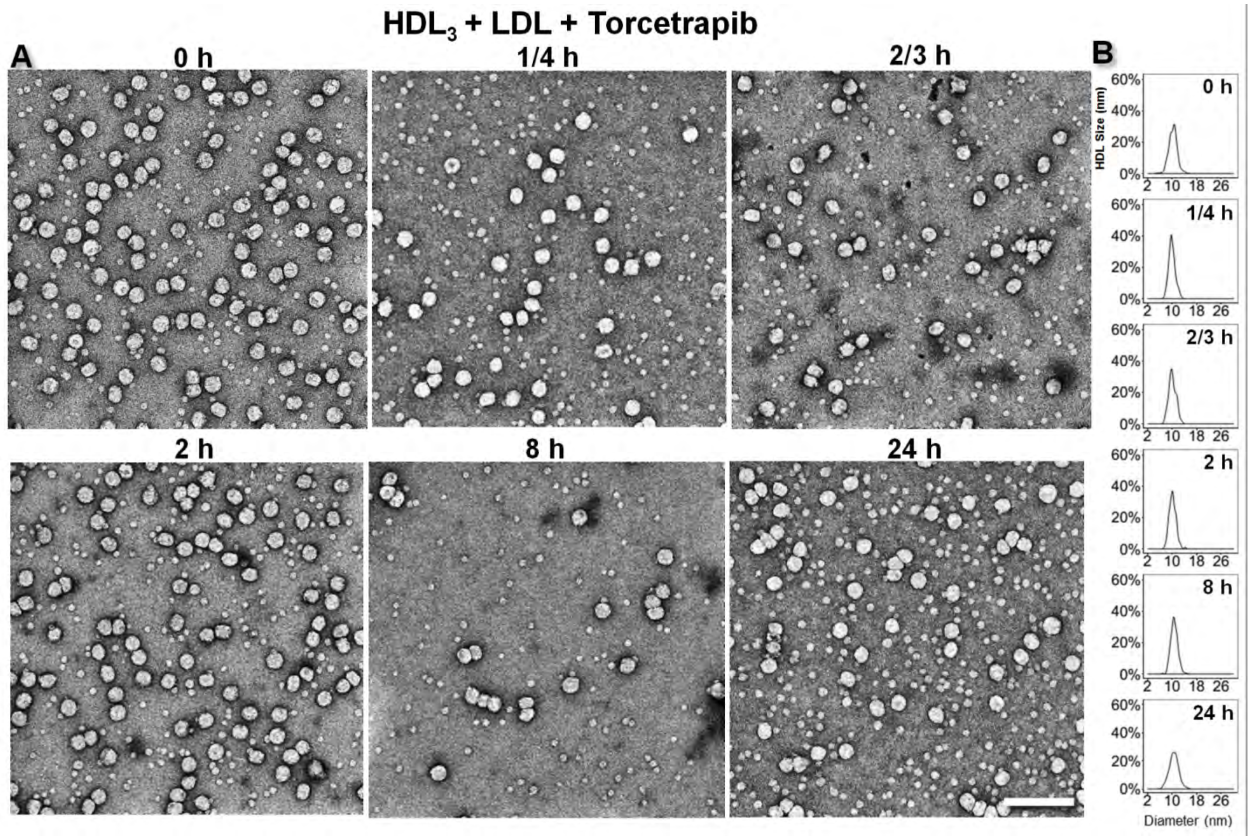

Supplemental Figure 26**. Structure of human plasma HDL_3 incubated with LDL and Torcetrapib, as shown by OpNS EM.** A**)** As a control, a sample of human plasma HDL_3 was incubated with LDL and Torcetrapib (without CETP) at 37°C for various lengths of time. Survey views of OpNS EM images of the samples after 6 representative incubation times (0 min, 15 min, 40 min, 2 hours, 8 hours and 24 hours) are presented. B) Histograms of HDL diameters measured from each EM imaged under each incubation time are shown. A total of 300–500 HDL_3 particles from each sample were used for the diameter measurement based on the geometric mean of two diameters of each HDL particle, the longest diameter and its perpendicular diameter. Scale bar: 120 nm.

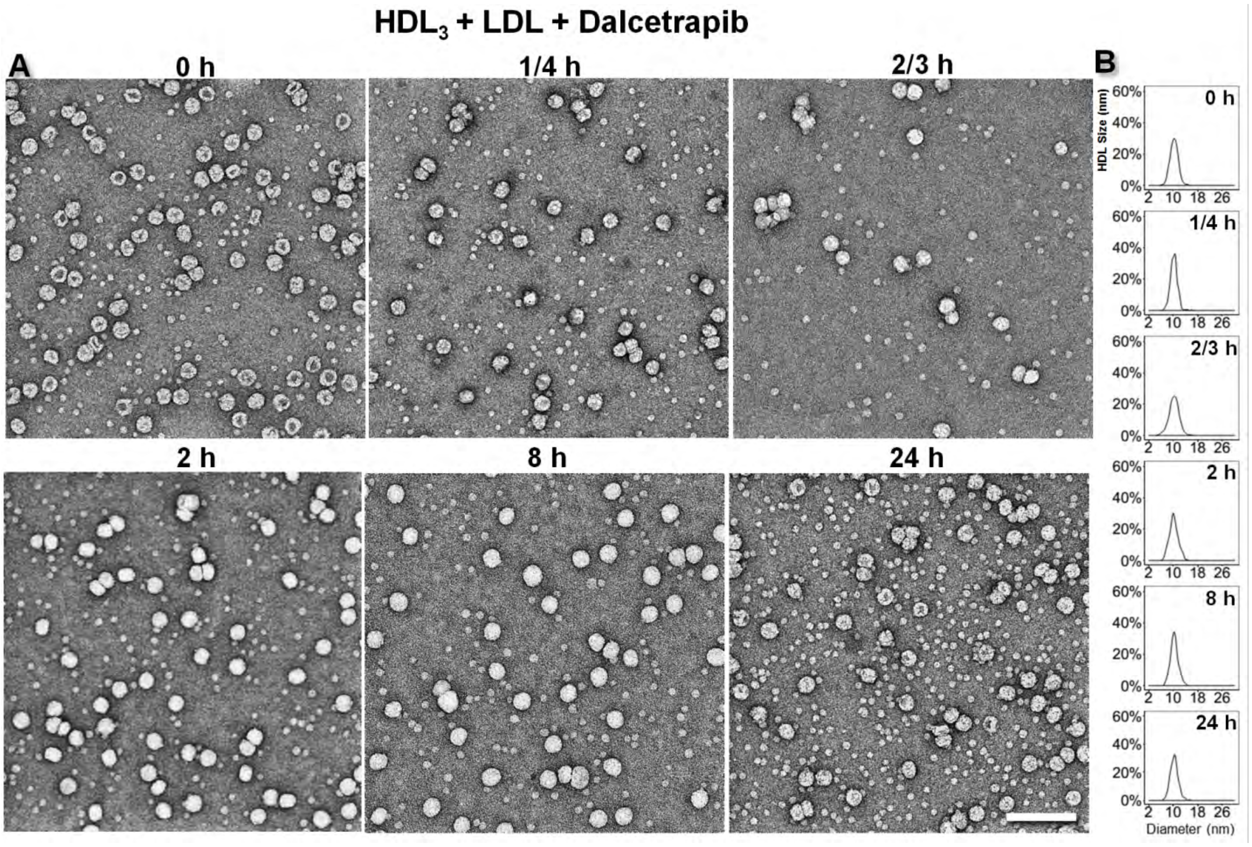

Supplemental Figure 27**. Structure of human plasma HDL_3 incubated with LDL and Dalcetrapib, as shown by OpNS EM.** A**)** As a control, a sample of human plasma HDL_3 was incubated with LDL and Dalcetrapib (without CETP) at 37°C for various lengths of time. Survey views of OpNS EM images of the samples after 6 representative incubation times (0 min, 15 min, 40 min, 2 hours, 8 hours and 24 hours) are presented. B) Histograms of HDL diameters measured from each EM imaged under each incubation time are shown. A total of 300–500 HDL_3 particles from each sample were used for the diameter measurement based on the geometric mean of two diameters of each HDL particle, the longest diameter and its perpendicular diameter. Scale bars: 120 nm.

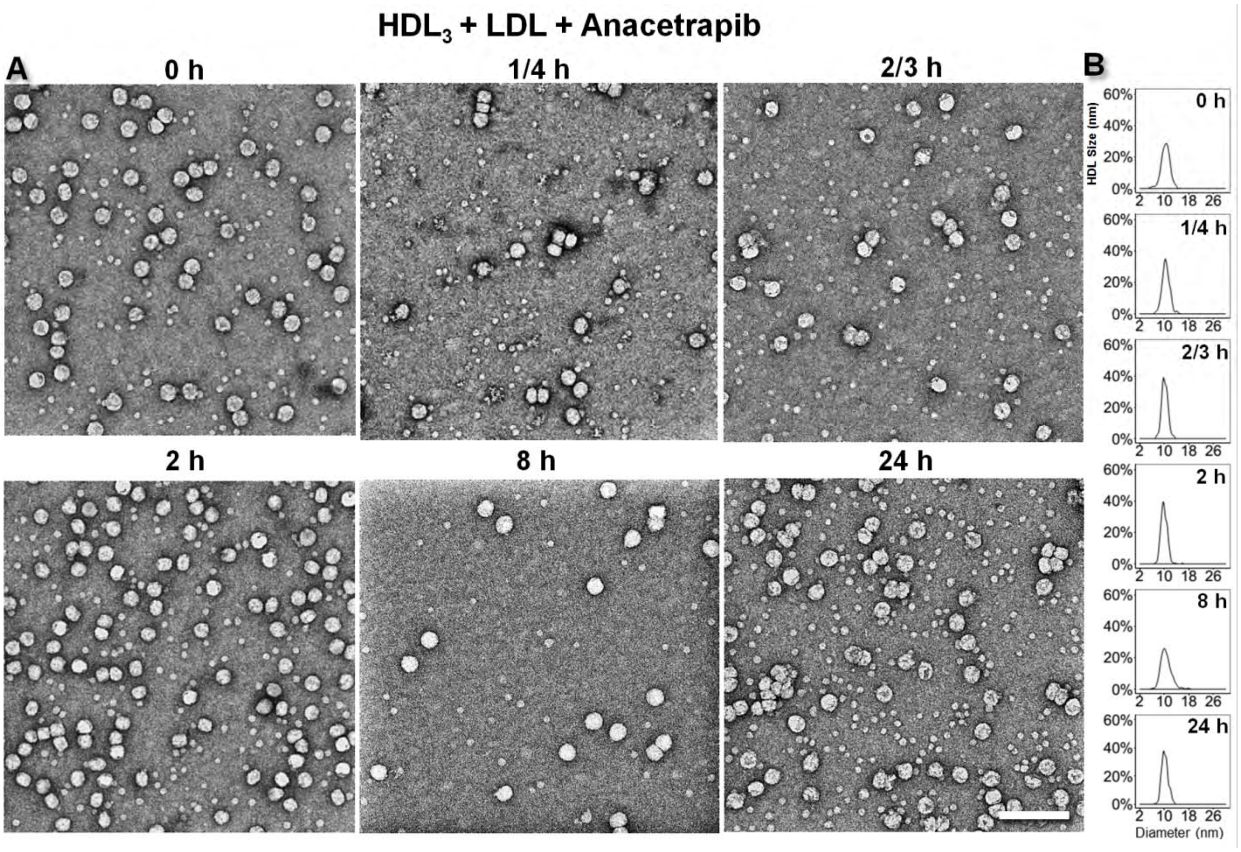

Supplemental Figure 28**. Structure of human plasma HDL_3 incubated with LDL and Anacetrapib, as shown by OpNS EM.** A**)** As a control, a sample of human plasma HDL_3 was incubated with LDL and Anacetrapib (without CETP) at 37°C for various lengths of time. Survey views of OpNS EM images of the samples after 6 representative incubation times (0 min, 15 min, 40 min, 2 hours, 8 hours and 24 hours) are presented. B) Histograms of HDL diameters measured from each EM imaged under each incubation time are shown. A total of 300–500 HDL_3 particles from each sample were used for the diameter measurement based on the geometric mean of two diameters of each HDL particle, the longest diameter and its perpendicular diameter. Scale bar: 120 nm.

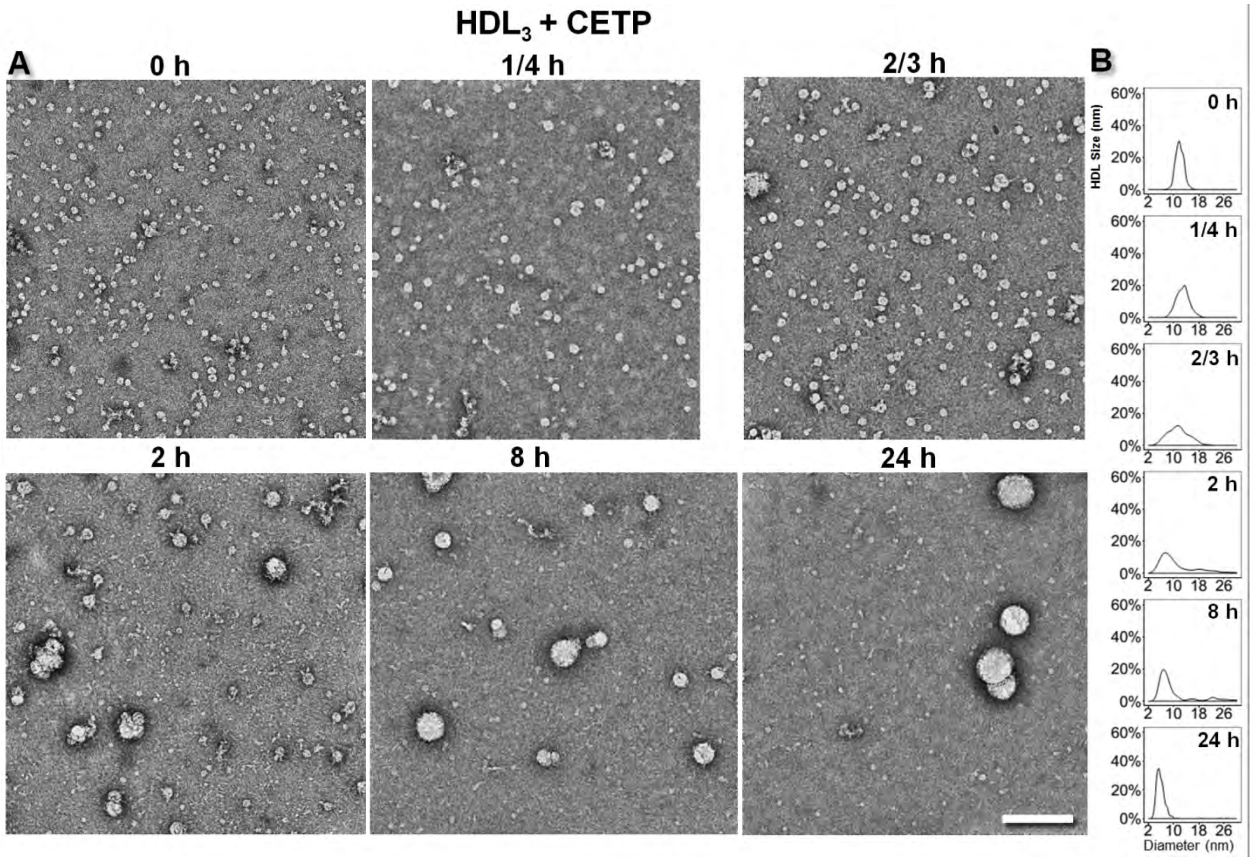

Supplemental Figure 29**. Structure of human plasma HDL_3 incubated with CETP, as shown by OpNS EM.** A**)** As an additional control, a sample of human plasma HDL_3 was incubated with CETP at 37°C for various lengths of time. Survey views of OpNS EM images of the samples after 6 representative incubation times (0 min, 15 min, 40 min, 2 hours, 8 hours and 24 hours) are presented. B) Histograms of HDL diameters measured from each EM imaged under each incubation time are shown. A total of 300–500 HDL_3 particles from each sample were used for the diameter measurement based on the geometric mean of two diameters of each HDL particle, the longest diameter and its perpendicular diameter. Scale bar: 120 nm.

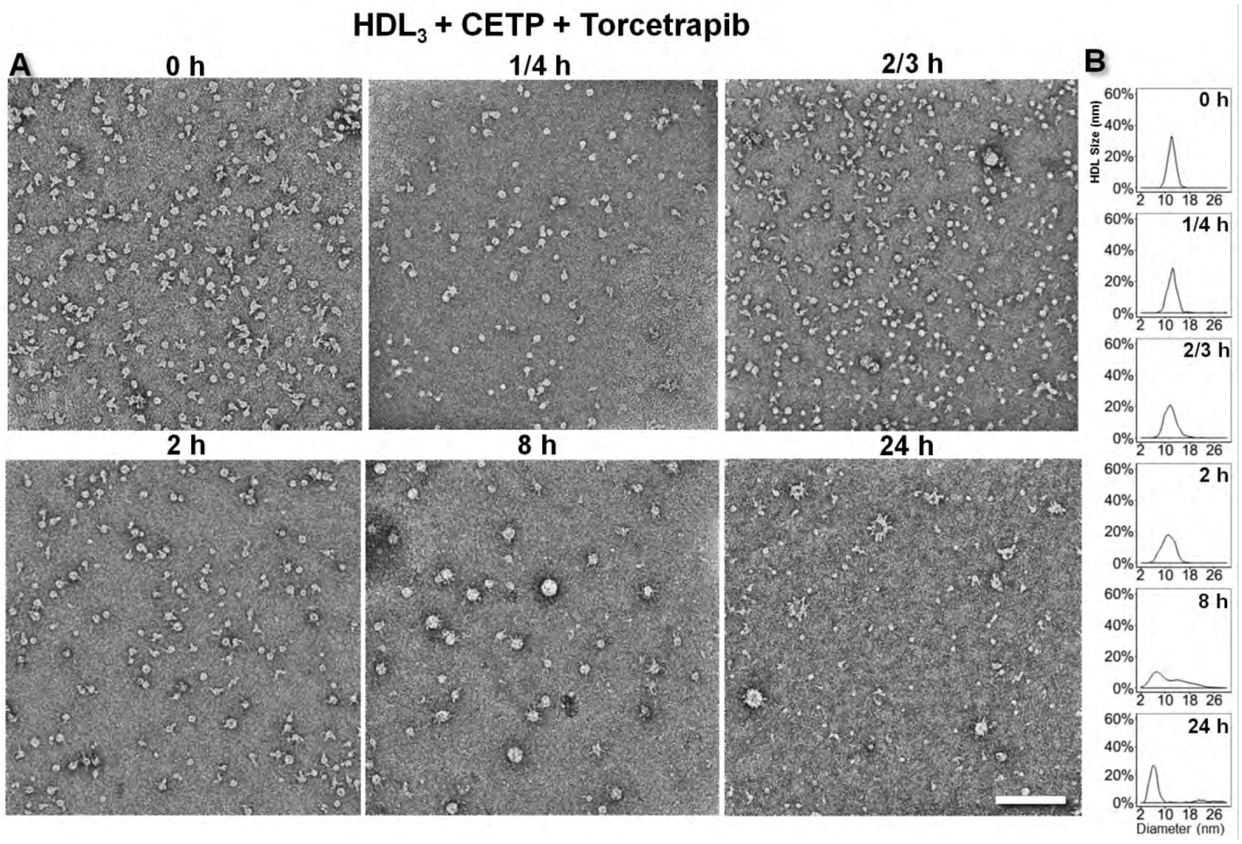

Supplemental Figure 30**. Structure of human plasma HDL$_3$ incubated with CETP and Torcetrapib, as shown by OpNS EM.** A**)** As a control, a sample of human plasma HDL$_3$ was incubated with CETP and Torcetrapib at 37°C for various lengths of time. Survey views of OpNS EM images of the samples after 6 representative incubation times (0 min, 15 min, 40 min, 2 hours, 8 hours and 24 hours) are presented. B) Histograms of HDL diameters measured from each EM imaged under each incubation time are shown. A total of 300–500 HDL$_3$ particles from each sample were used for the diameter measurement based on the geometric mean of two diameters of each HDL particle, the longest diameter and its perpendicular diameter. Scale bar: 120 nm.

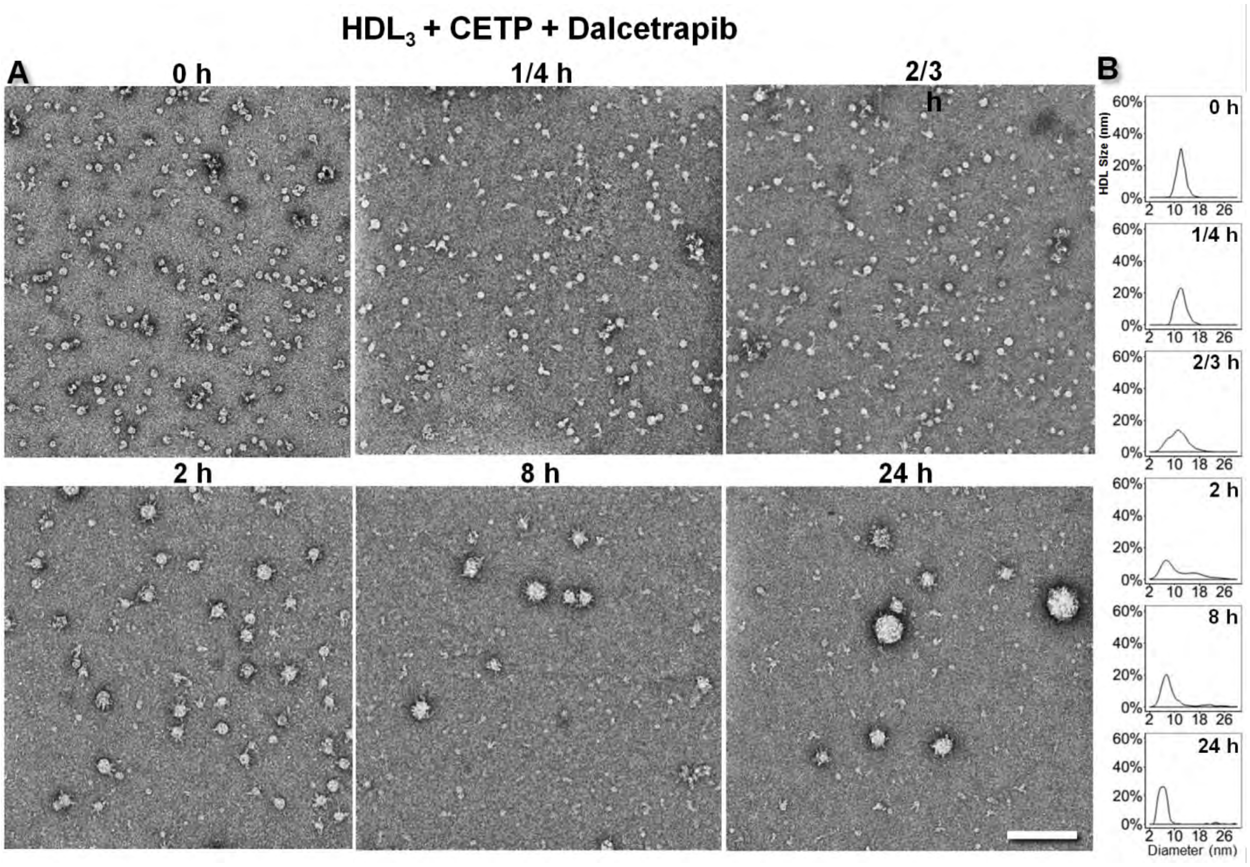

Supplemental Figure 31**. Structure of human plasma HDL_3 incubated with CETP and Dalcetrapib, as shown by OpNS EM.** A**)** As a control, a sample of human plasma HDL_3 was incubated with CETP and Dalcetrapib at 37°C for various lengths of time. Survey views of OpNS EM images of the samples after 6 representative incubation times (0 min, 15 min, 40 min, 2 hours, 8 hours and 24 hours) are presented. B) Histograms of HDL diameters measured from each EM imaged under each incubation time are shown. A total of 300–500 HDL_3 particles from each sample were used for the diameter measurement based on the geometric mean of two diameters of each HDL particle, the longest diameter and its perpendicular diameter. Scale bar: 120 nm.

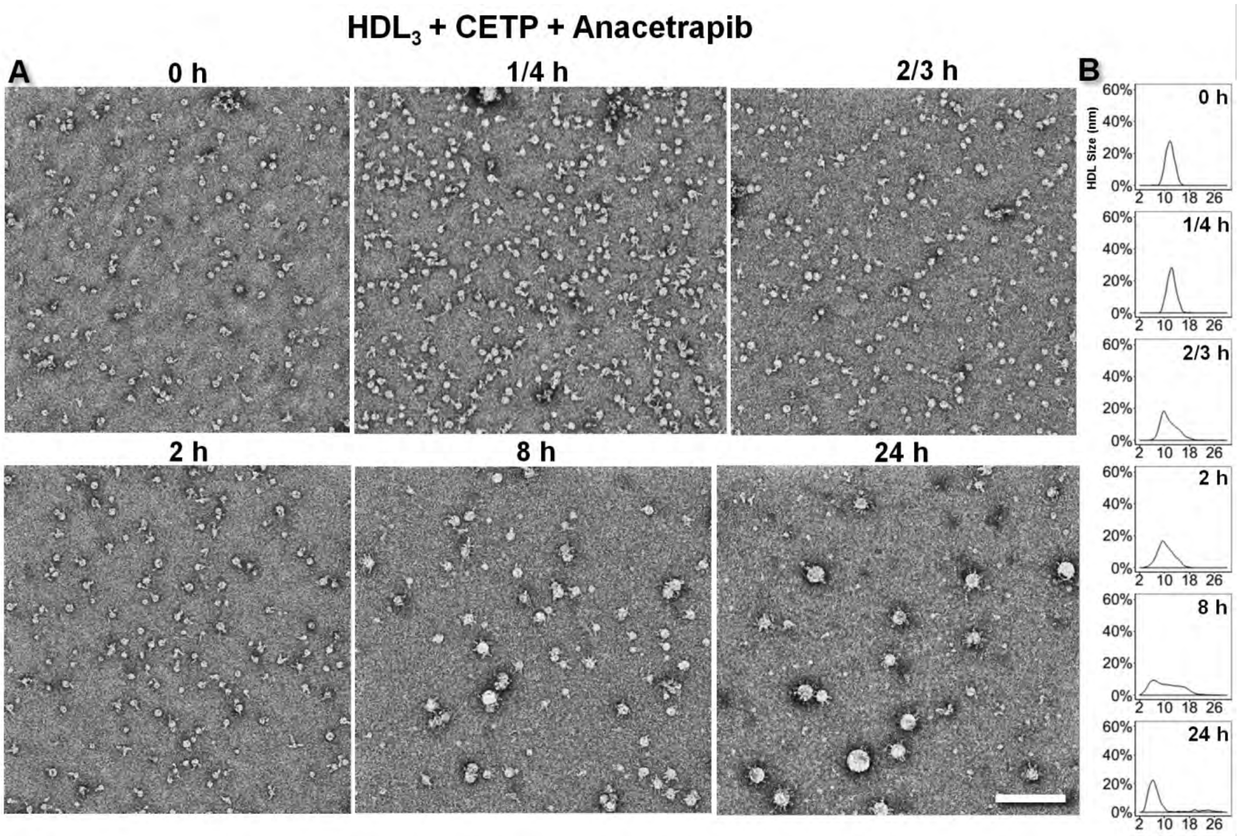

Supplemental Figure 32**. Structure of human plasma HDL_3 incubated with CETP and Anacetrapib, as shown by OpNS EM.** A**)** As a control, a sample of human plasma HDL_3 was incubated with CETP and Anacetrapib at 37°C for various lengths of time. Survey views of OpNS EM images of the samples after 6 representative incubation times (0 min, 15 min, 40 min, 2 hours, 8 hours and 24 hours) are presented. B) Histograms of HDL diameters measured from each EM imaged under each incubation time are shown. A total of 300–500 HDL_3 particles from each sample were used for the diameter measurement based on the geometric mean of two diameters of each HDL particle, the longest diameter and its perpendicular diameter. Scale bar: 120 nm.
